# The pathogenesis of diclofenac induced immunoallergic hepatitis in a canine model of liver injury

**DOI:** 10.18632/oncotarget.21201

**Published:** 2017-09-23

**Authors:** Saravanakumar Selvaraj, Jung-Hwa Oh, Reinhard Spanel, Florian Länger, Hyoung-Yun Han, Eun-Hee Lee, Seokjoo Yoon, Jürgen Borlak

**Affiliations:** ^1^ Centre for Pharmacology and Toxicology, Hannover Medical School, 30625 Hannover, Germany; ^2^ Department of Predictive Toxicology, Korea Institute of Toxicology, 34114 Gajeong-ro, Yuseong, Daejeon, Republic of Korea; ^3^ Institute of Pathology, 41747 Viersen, Germany; ^4^ Institute of Pathology, Hannover Medical School, 30625 Hannover, Germany

**Keywords:** diclofenac, immunogenomics, complement system, classical and alternate pathway, CARPA

## Abstract

Hypersensitivity to non-steroidal anti-inflammatory drugs is a common adverse drug reaction and may result in serious inflammatory reactions of the liver. To investigate mechanism of immunoallergic hepatitis beagle dogs were given 1 or 3 mg/kg/day (HD) oral diclofenac for 28 days. HD diclofenac treatment caused liver function test abnormalities, reduced haematocrit and haemoglobin but induced reticulocyte, WBC, platelet, neutrophil and eosinophil counts. Histopathology evidenced hepatic steatosis and glycogen depletion, apoptosis, acute lobular hepatitis, granulomas and mastocytosis. Whole genome scans revealed 663 significantly regulated genes of which 82, 47 and 25 code for stress, immune response and inflammation. Immunopathology confirmed strong induction of IgM, the complement factors C3&B, SAA, SERPING1 and others of the classical and alternate pathway. Alike, marked expression of CD205 and CD74 in Kupffer cells and lymphocytes facilitate antigen presentation and B-cell differentiation. The highly induced HIF1A and KLF6 protein expression in mast cells and macrophages sustain inflammation. Furthermore, immunogenomics discovered 24, 17, 6 and 11 significantly regulated marker genes to hallmark M1/M2 polarized macrophages, lymphocytic and granulocytic infiltrates; note, the latter was confirmed by CAE staining. Other highly regulated genes included alpha-2-macroglobulin, CRP, hepcidin, IL1R1, S100A8 and CCL20. Diclofenac treatment caused unprecedented induction of myeloperoxidase in macrophages and oxidative stress as shown by SOD1/SOD2 immunohistochemistry. Lastly, bioinformatics defined molecular circuits of inflammation and consisted of 161 regulated genes.

Altogether, the mechanism of diclofenac induced liver hypersensitivity reactions involved oxidative stress, macrophage polarization, mastocytosis, complement activation and an erroneous programming of the innate and adaptive immune system.

## INTRODUCTION

Diclofenac is a nonsteroidal anti-inflammatory drug (NSAID) commonly used to treat mild-to-moderate pain in rheumatoid and osteoarthritis as well as musculoskeletal injuries [1, 2]. Annually > 1000 tons of diclofenac are produced in the form of capsules, tablets, ointments and intravenous solution, thus underlining its extensive use [3, 4]*. This NSAID* exerts anti-inflammatory, analgesic and anti-pyretic effects through different mechanisms [5]. It inhibits cyclooxygenase 1 and 2 at an IC50 of 0.076 and 0.026μM, respectively and therefore modulates arachidonic acid metabolism and its pool size [6]. Diclofenac also inhibits production of leukotrienes through inhibition of lipoxygenases [7] and suppresses prostaglandin synthesis and thromboxane-prostanoid receptor signaling. Its analgesic activity partially resides in an activation of the nitric oxide–cGMP nociceptive pathway and inhibition of NMDA receptor mediated hyperalgesia. Diclofenac also inhibits activity of the neuropeptide substance P and is a partial agonist of the peroxisome proliferator activated receptor gamma (PPARγ) [8].

Repeatedly, the safety of diclofenac was assessed by regulatory authorities [9] and next to cardiovascular complications diclofenac causes liver and kidney injury especially among chronic drug users. According to the NIH LiverTox database serum liver function tests may be elevated in up to 15% of patients [10], and a long-term prospective clinical trial involving 17,289 arthritis patients revealed diclofenac to be commonly associated with aminotransferase elevations [11]. Likewise, a study on the incidence, presentation and outcomes in patients with drug-induced liver injury (DILI) in the general population of Iceland reported diclofenac to rank second among DILI causing agents [12].

Some of the reasons for diclofenac to cause DILI have been summarized by Boelsterli, 2003 and Aithal, 2011 [13, 14] and the role of reactive metabolites was emphasized. Next to direct effects reactive metabolites produce hapten-protein conjugates which are sensed and phagocytozed by antigen presenting cell (APC); when co-expressed with the major histocompatibility complex APCs elicit B-cell (drug antibody) and T-cell responses [15].

In an effort to develop an assay predictive for drug induced hepatitis, lymphocytes from different clinical DILI cases were isolated from heparinized blood [16]. The lymphocytes proliferated *in vitro* when exposed to the parent drug. A similar result was obtained when drug antigens obtained from serum of healthy individuals were added to the lymphocyte cultures to suggest an immune response independent of reactive metabolite [16]. Moreover, stimulation of lymphocytes was amplified when treated with the NSAID indomethacin.

Recently, we reported an identification of molecular circuits of diclofenac induced liver injury in mice [17] and observed induced cytokine and chemokine release by injured cells and activated immune cells like neutrophils, lymphocytes and macrophages. The release of inflammatory mediators supports migration and infiltration of immune competent cells at the site of injury to result in complex pro-and anti-inflammatory reactions in the course of immune-mediated hepatic injury. Specifically, release of pro-inflammatory cytokines and chemokines by macrophages, T cells and T helper (Th) cells such as interferon (IFN)γ, the interleukins (IL)-1, IL-6, IL-17, IL-18, the CXC chemokines and ligands of the chemokine receptors, i.e. CXCL1 and CXCL2, exacerbate diclofenac induced liver and kidney injury [18, 19]. In mice Th-17 mediated inflammation leads to DILI [20–22]; there is also suspicion that genetic polymorphism of immune response genes such as IL-4 and IL-10 sensitize to drug induced hepatotoxicity [15, 23].

Diclofenac induced idiosyncratic liver injury was shown to be immune-mediated; yet the underlying mechanisms remain unclear. Given the differences in the immune system between animals and humans [24] and the idiosyncratic nature of the reaction it is difficult to develop an animal model predictive for clinical DILI. Furthermore, pre-treatment of animals with inflammagens such as TNFα or LPS elicit an acute systemic inflammatory response which is different from drug induced inflammation. Moreover, in clinical DILI the onset of immune-mediated injury is usually delayed. Note, the relevance of animal studies to predict human toxicity was evaluated by an expert panel [25]. A total of 150 drugs encompassing a wide range of therapeutic classes was assessed by comparing animal data with clinically observed adverse drug reactions (ADR). The concordance was 71% when rodent and non-rodent data was considered together; however, dog studies alone were superior and predictive for 63% of clinical cases. The data involved 468 repeated-dose toxicity studies using primarily rats and dogs. Moreover, in the case of concordant/predictive toxicity 94% of animal studies predicted clinical ADR within 4 weeks or less of treatment [25]. Collectively, toxicities seen in dogs were more predictive for human ADR which provided a rational for the use of the canine animal model to investigate mechanism of diclofenac induced immunoallergic hepatitis. Further justification of the canine model is the similar COX1/COX2 expression between dogs and humans [26].

To better comprehend mechanism of diclofenac induced immuneallergic hepatitis we performed an immunogenomic study after repeated treatment for 28 days. Next to histopathology and serum/urinary biochemistry measurements the findings were corroborated by immunohistochemistry. Transcriptomics identified regulated immune, stress and inflammatory response genes, and the subsequently performed network analysis provided insight into mechanisms of diclofenac induced hypersensitivity reactions. Collectively, diclofenac treatment caused an activation of the complement system of the classical and alternate pathway with features of CARPA but without anaphylaxis. The allergic reaction is triggered by an erroneous programing of the innate and adaptive immune system and hallmarked by granulomatous hepatitis and mastocytosis of the liver.

## RESULTS

### Treatment related clinical signs

**Figure 1:**
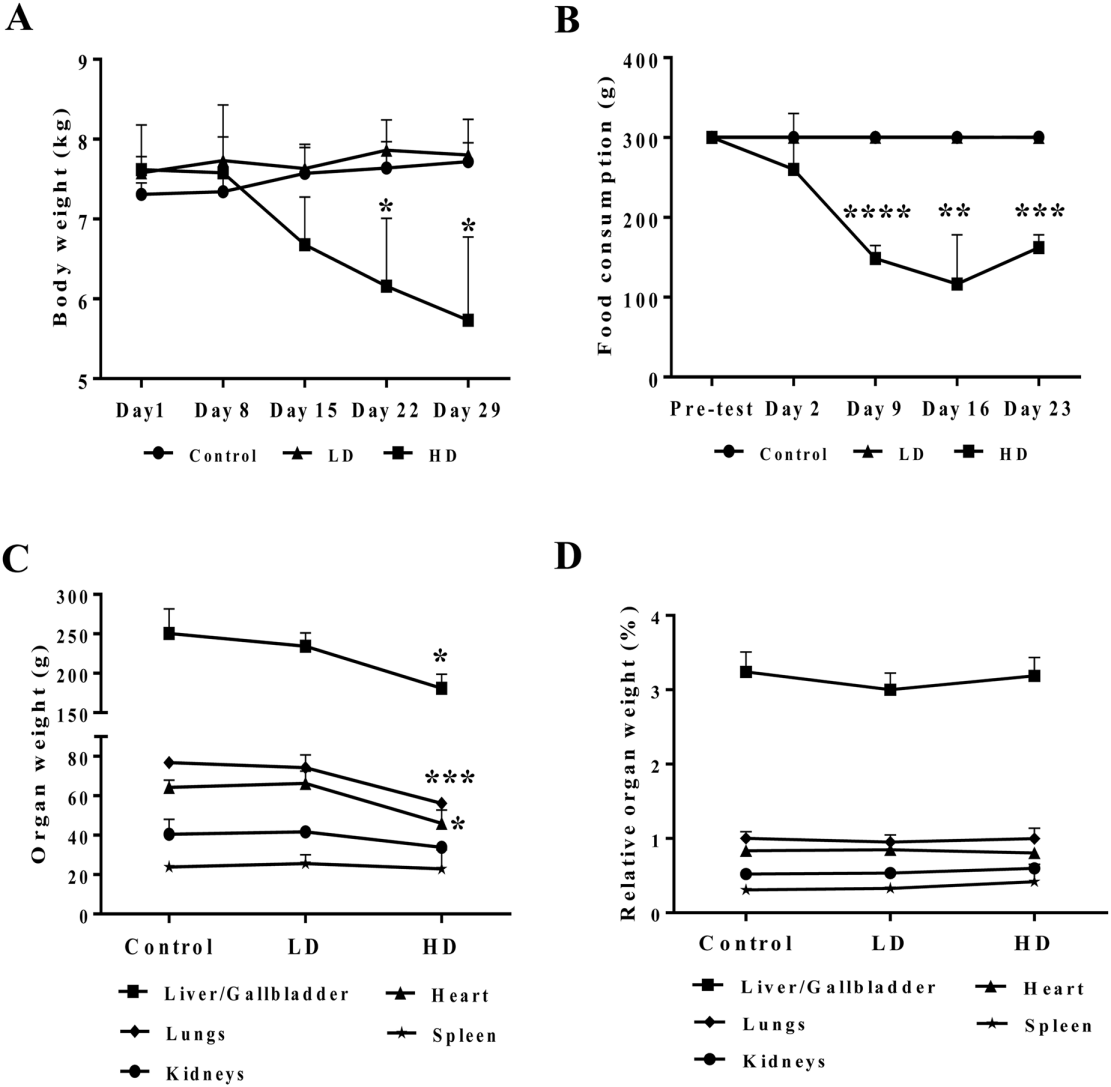
Body weight and food consumption after repeated diclofenac treatment for 28 days **(Panel A)** Body weight. **(Panel B)** Food consumption. **(Panel C)** Selected organ weights. **(Panel D)** Body weight adjusted organ weights. LD = low dose, HD = high dose, ^*^p<0.05, ^**^p<0.01, ^***^p<0.001, ^****^p<0.0001.

Figure 1 depicts the body weight (panel A) and food consumption (panel B) that was monitored over the entire treatment period in addition to individual organ weights determined at the end of the study (panel C). Only at the high dose regimen a significant reduction in body weight, food consumption and absolute organ weights of liver, lung and heart was noted. However, the difference in organ weights disappeared when adjusted for the total body weight (panel D).

### Serum biochemistries

**Figure 2:**
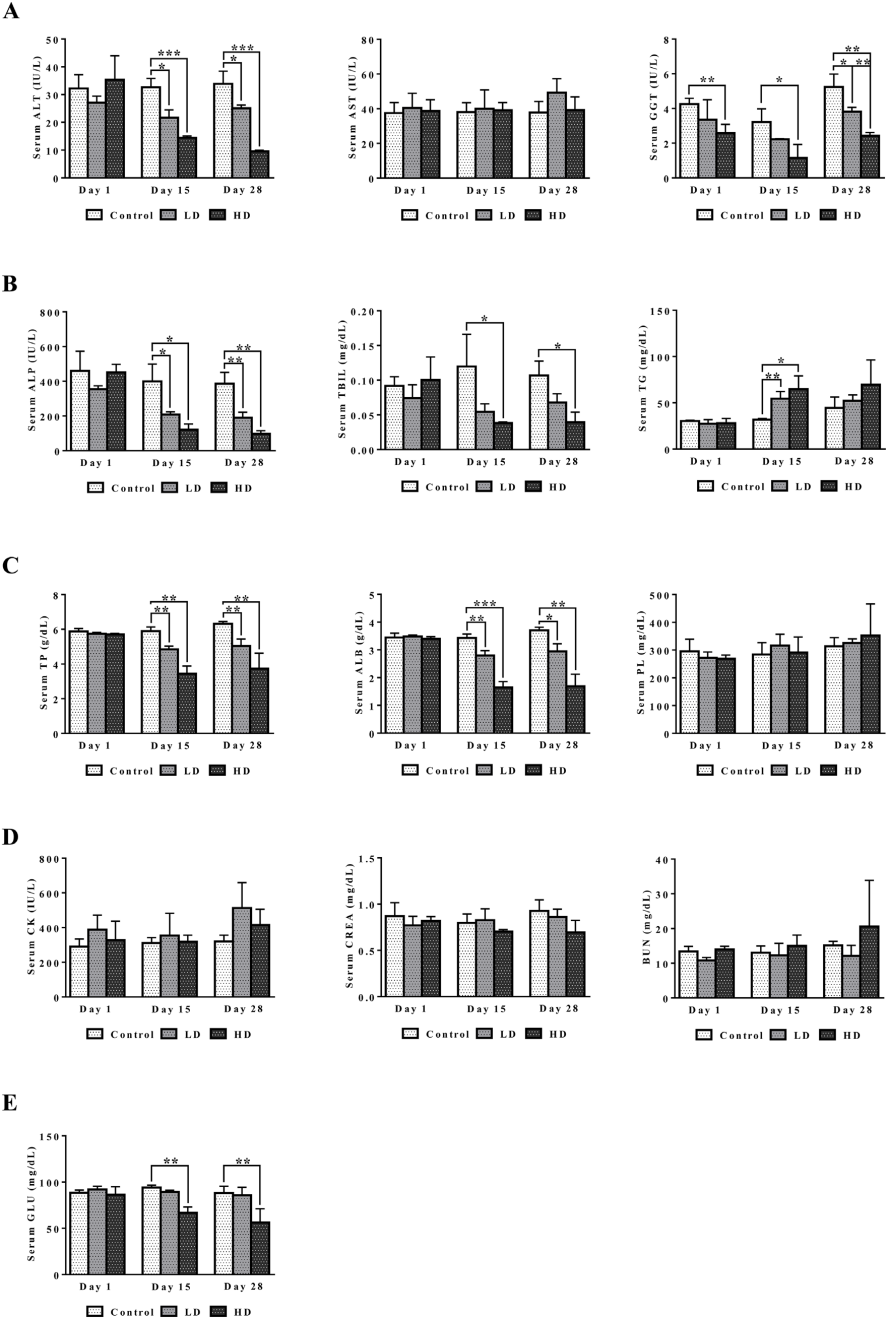
Serum biochemistries after repeated diclofenac treatment for 28 days **(Panel A1-A3)** ALT, AST and γGT. **(Panel B1-B3)** ALP, TBIL, TG. **(Panel C1-C3)** TP, ALB, PL. **(Panel D1-D3)** CK, CREA, BUN. **(Panel E)** Serum glucose. LD = low dose, HD = high dose, ^*^p<0.05, ^**^p<0.01, ^***^p<0.001.

A summary of serum biochemistry findings is given in Figure 2. Treatment related abnormalities include the significant and dose dependent hypoalbuminemia and hypoproteinemia as well as significant reductions in ALT, ALP and γGT. The data are suggestive for an inflammation and wasting syndrome related cause of injury. Total bilirubin and serum glucose was also dose dependently reduced, and the observed hypoglycemia may be linked to altered ion channel activity of insulin secreting beta cells as was demonstrated for several NSAIDs [27]. Conversely, serum triglyceride was significantly (day 15) and serum phospholipid insignificantly increased. Moreover, serum CK and BUN were insignificantly increased. Although the changes may be related to the weight loss and mild kidney injury serum creatinine was basically unchanged or even reduced at high dose regimen.

### Treatment related hematologic disorders

**Figure 3:**
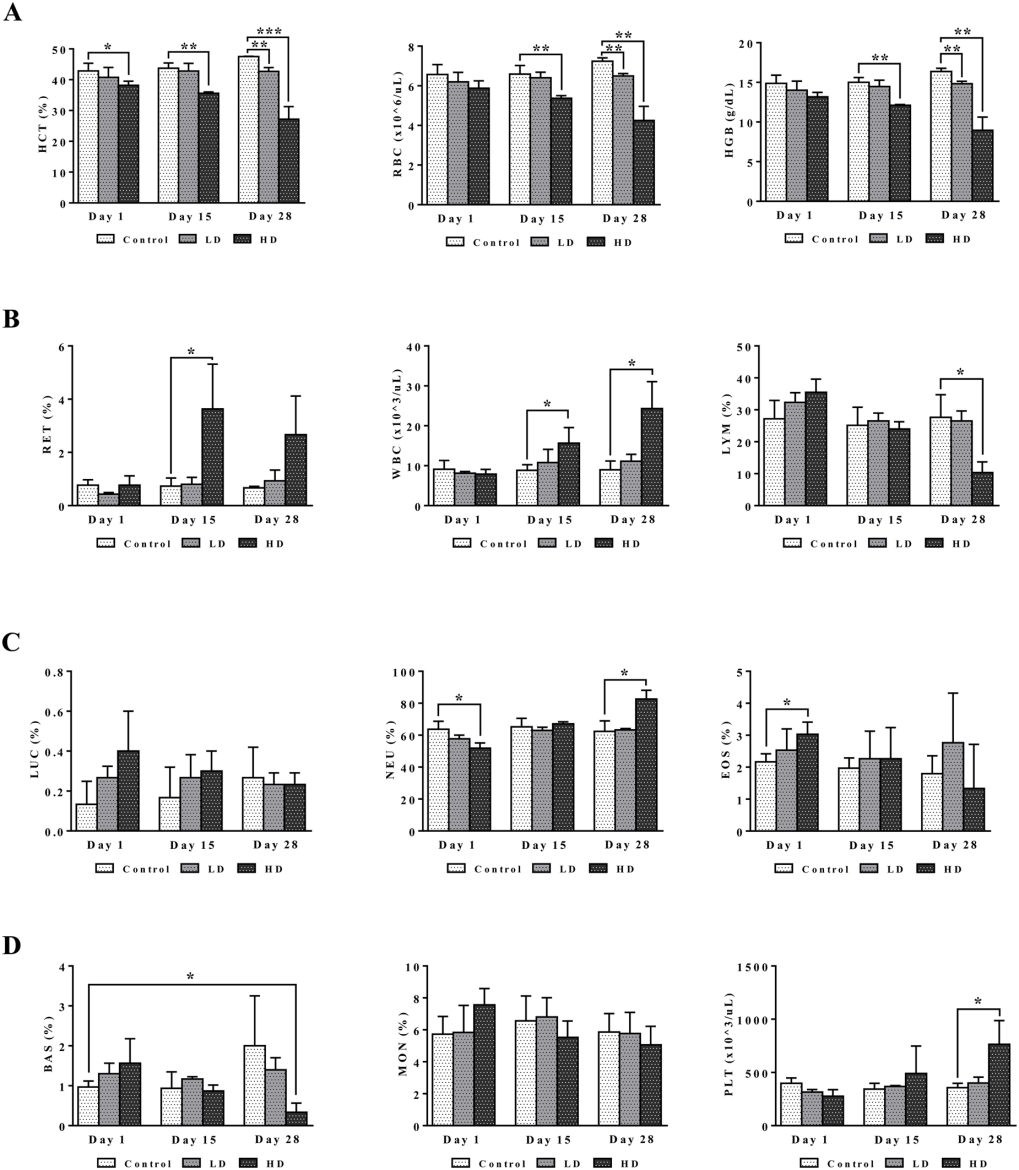
Haematology read-outs after repeated diclofenac treatment for 28 days **(Panel A1-A3)** HCT, RBC, HGB. **(Panel B1-B3)** RET, WBC, LYMPH. **(Panel C1-C3)** LUC (large undifferentiated cells), neutrophils, eosinophils. **(Panel D1-D3)** basophils, monocytes, platelets. LD = low dose, HD = high dose, ^*^p<0.05, ^**^p<0.01, ^***^p<0.001.

Blood smear testing was done on days 1, 15 and 28 of diclofenac treatment. A dose dependent reduction in haematocrit, red blood cell count and haemoglobin was determined to signify bone marrow toxicity particularly at the high dose (HD) for 28 days (Figure 3). The significant increase in the proportion of reticulocytes indicates adaptive responses to the treatment related haemolytic anaemia and to compensate for erythrocytopenia. Furthermore, the dose and time dependent increases in WBC and platelets together with the highly significant induction of serum amyloid A1, IgG, IgM, complement factors, cytokines, chemokines and acute phase reactants (data are given below) provide strong evidence for drug induced inflammation. The significant increases in eosinophiles (day 1) and neutrophiles (day 28) are testimony to a drug induced inflammatory process though basophiles were repressed after repeated HD treatment for 28 days. Lymphocytopenia was also observed and is likely caused by inflammation that is sustained by the interplay of activated granulocytes, monocytes, Kupffer cells, mast cells and other pro-inflammatory mediators as defined by histopathology (see below). Notwithstanding, diclofenac hypersensitivity may also arise from complement activation-related pseudoallergy (CARPA) and is associated with granulocyte and mast cell degranulation with the release of pro-inflammatory molecules, histamines and other cytotoxic molecules as described below.

### Serum and urinary electrolyte disturbances

**Figure 4:**
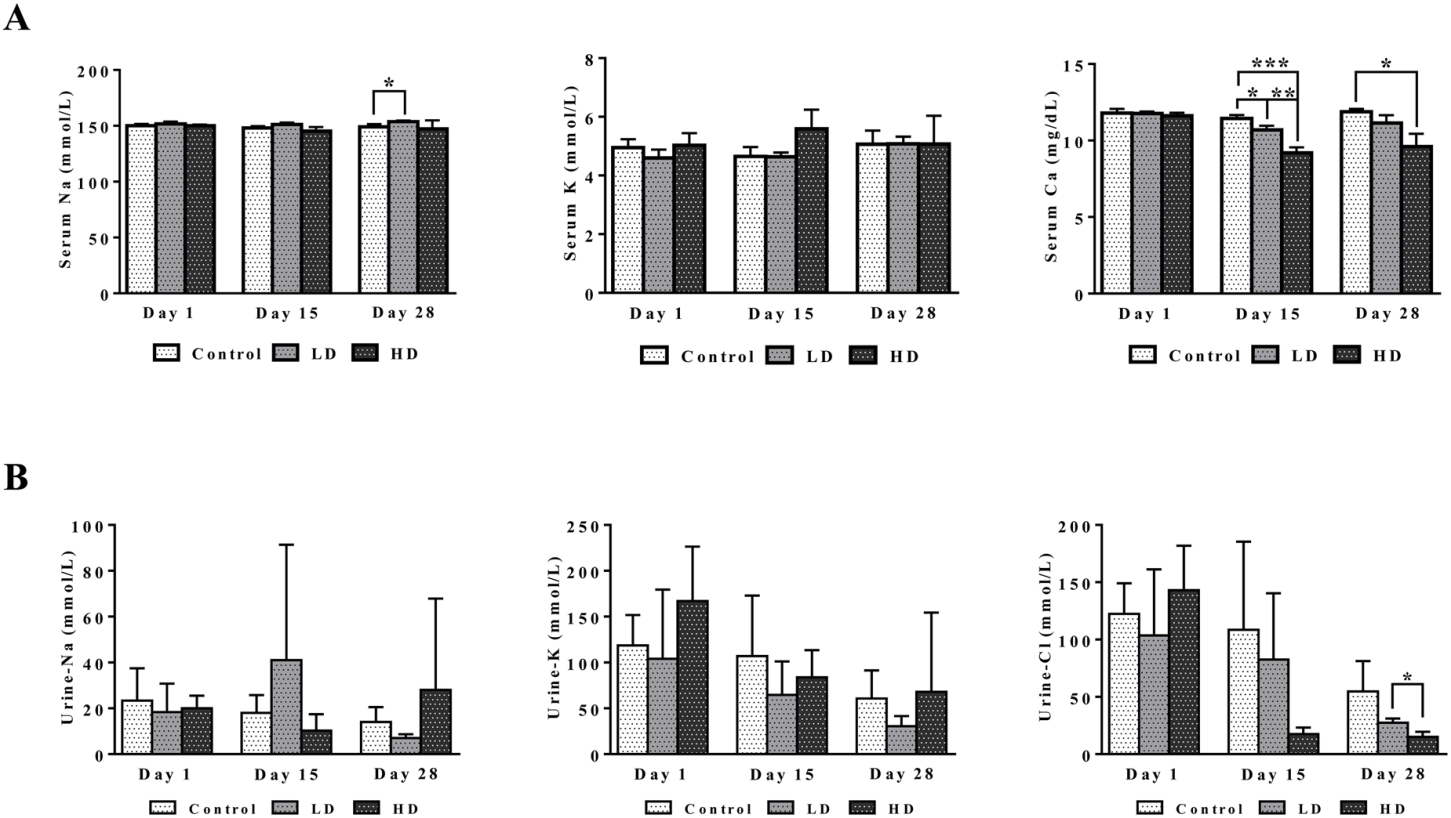
Serum and urine electrolytes after repeated diclofenac treatment for 28 days **(Panel A1-A3)** Serum Na, K and Ca. **(Panel B1-B3)** Urine Na, K and Cl. LD = low dose, HD = high dose, ^*^p<0.05, ^**^p<0.01, ^***^p<0.001.

Figure 4 depicts dose dependent reductions in serum Ca2+ and urine Cl-ions. It is of considerable importance that kidney SLC12A2 was induced by >5-fold while carbonic anhydrase (CA) was repressed by nearly 25- and 2-fold in liver and kidney, respectively (data given below). Both proteins influence calcium homeostasis. Note, thiazide diuretics are given to patients with nephrolithiasis to inhibit activity of the SLC12A2 cation chloride-coupled co-transporter while inhibitors of carbonic anhydrase may increase calcium excretion [28]. Collectively, the observed induction of SLC12A2, the repression of CA and the 3-fold induction of the associated bicarbonate anion exchanger SLC26A4 (Pendrin) provide a molecular rationale for the observed electrolyte imbalances. Furthermore, CA functions as a radical scavenger and was shown to protect cells from hydrogen peroxide induced apoptosis [29, 30]. Given the significant induction of peroxidase in response to diclofenac treatment (see also Figure 11, MPO staining of Kupffer cells) and the significant regulation of CA in liver and kidney we consider oxidative damage as a likely cause for the observed electrolyte disturbances.

**Figure 5:**
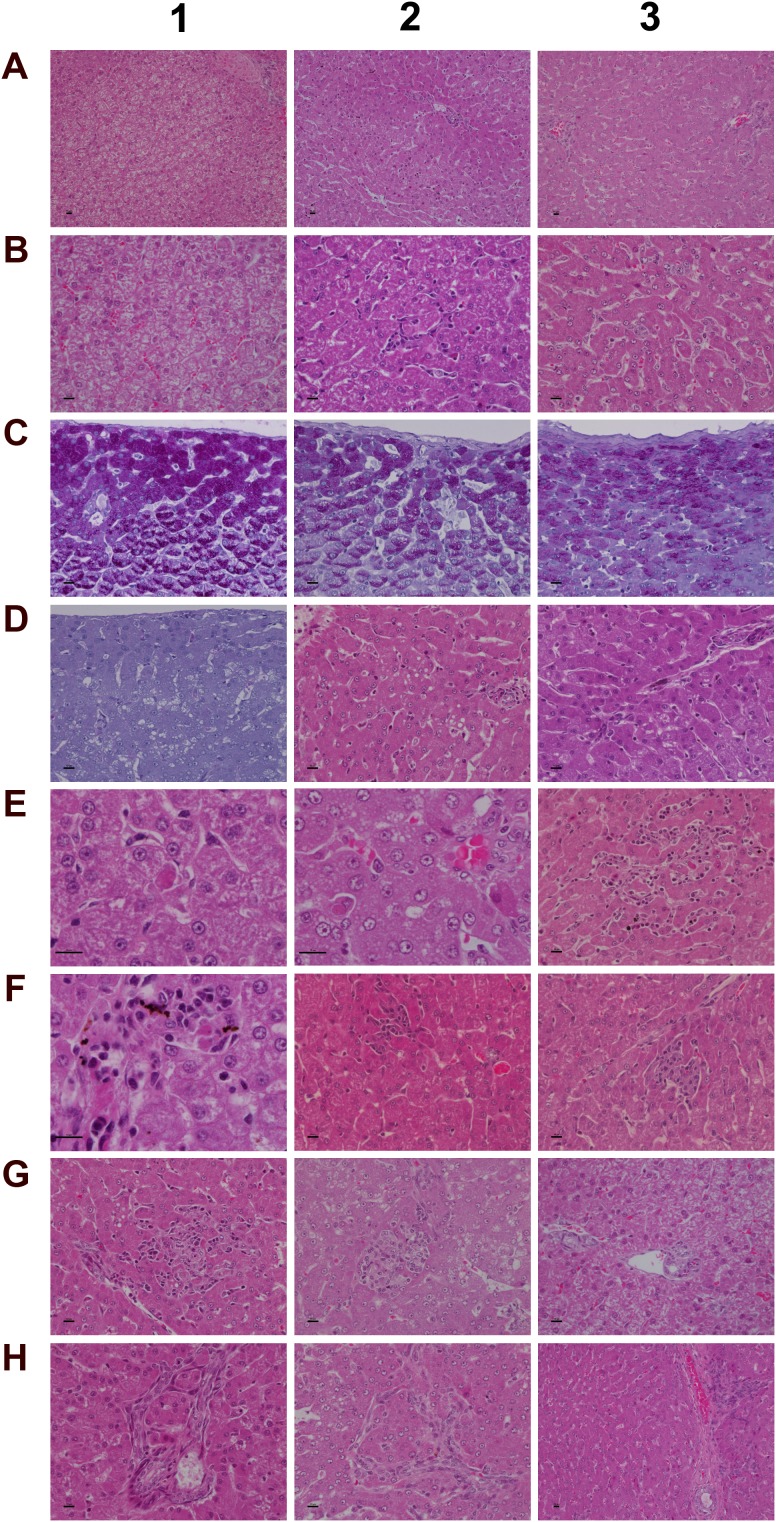
Histology of the liver in control and diclofenac treated animals after daily dosing for 28 days **(Panel A1)** H&E staining of a control animal with monolayered trabecular organized hepatocytes and no sign of morphological alteration. **(Panel A2)** H&E staining of a low dose treated animal with enlarged and partially bilayered trabeculae; hepatocytes present a basophilic cytoplasm. Regenerative periportal hepatocytes are smaller in size. **(Panel A3)** H&E staining of a high dose treated animal; marked hepatic lobular regeneration with predominatly bilayered trabeculae. **(Panel B1)** Higher magnification of a liver section of a control animal. The nuclei of hepatocytes are round with small nucleoli. The cytoplasm is cloudy and rich in glycogen. **(Panel B2)** Higher magnification of a low dose treated animal. The nuclei appear moderately activated; focal sinusoidal inflammatory infiltrates are seen. **(Panel B3)** Higher magnification of a high dose treated animal with activated vesicular nuclei and prominent nucleoli. Zones of regeneration are organized as bilayered trabeculae with smaller sized hepatocytes and a slight basophilic cytoplasm. **(Panel C1)** PAS staining of a control liver section. Hepatocytes are rich in glycogen; the glycogen is preserved at the best beneath the capsule and results in marked staining. **(Panel C2)** PAS staining of a low dose treated animal. A patchier staining is observed with some hepatocytes presenting slight to moderate glycogen depletion. **(Panel C3)** PAS staining of a high dose treated animal. A patchier staining is observed with some hepatocytes presenting slight to moderate glycogen depletion. **(Panel D1)** PAS staining of a high dose treated animal with marked and almost complete glycogen depletion. Note the pronounced periportal hepatic steatosis induced by diclofenac treatment. **(Panel D2)** H&E staining of a high dose treated animal with a patchier periportal micro- and macrovesicular hepatic steaosis. **(Panel D3)** H&E staining of a low dose treated animal with focal mainly microvesicular hepatic steaosis. **(Panel E1)** Higher magnification of H&E staining of a low dose treated animal. In the reticle (center of the frame) a fresh apoptotic hepatocyte and its sinusoidal phagocytosis by a Kupffer cell is seen. **(Panel E2)** Higher magnification of H&E staining of a high dose treated animal. Shown in the lower right quadrant is an initial hepatocyte apoptosis within a steatotic liver section. Note the sinusiodal phagocytized apoptotic hepatocyte by a macrophage in the lower left quadrant. **(Panel E3)** H&E staining of a low dose treated animal with focal mixed inflammatory cellular infiltrates and groups of apoptotic hepatocytes. **(Panel F1-G2)** Sequential consolidation of focal interstitial inflammatory infiltrates with final granuloma formation of low (F1-F3) and high dose (G1-G2) treated animals. Panel F1 depicts at higher magnification a rounded focal sinusoidal/interstitial cellular infiltrate that is intermingled with remnants of apoptotic cells of a low dose treated animal. Early consolidation still exhibits a sinusoidal distribution of the infiltrate (F2), ending in a rounded granuloma (F3). Panel G1 depicts a rather acute interstitial infiltrate, and Panel G2 documents the consolidated granuloma in liver sections of high dose treated animals with marked steatosis. **(Panel G3)** H&E staining of a liver section of a control animal focussing on a normal portal field. **(Panel H1 and H2)** H&E staining of a low (H1) and high (H2) dose treated animal. Depicted are tangential sections of a portal field with marked arteriolocapillary and cholangiolar proliferations. **(Panel H3)** H&E staining of a high dose treated animal. Shown is an intact core of a transversely sectioned intact portal field. The proliferations occupy part of the limiting plate as well as periportal regions and likely trigger septal alterations as a result of increased hepatocyte regeneration. The bar represents 10μm.

**Figure 6:**
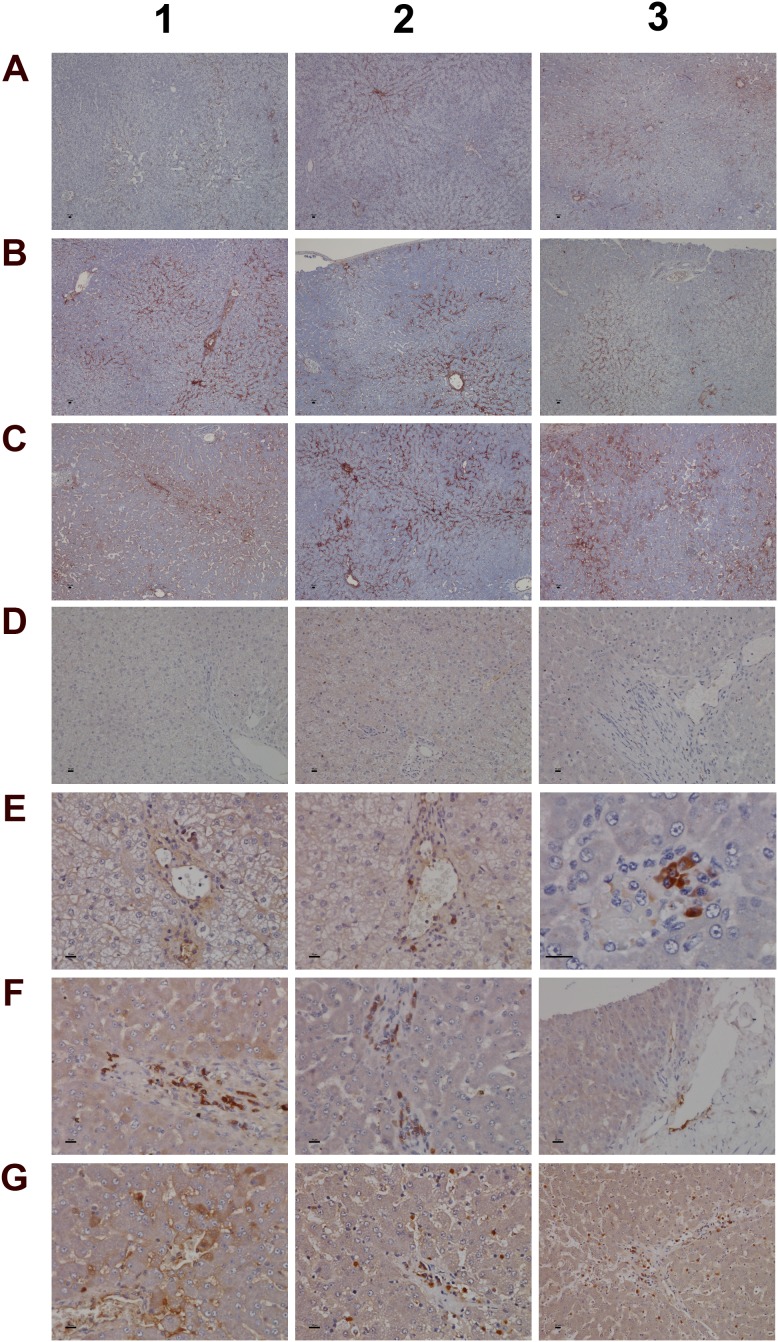
Immunohistochemistry staining of IgM and complement factor B in liver sections of control and diclofenac treated animals after daily dosing for 28 days **(Panel A1-A3)** Shown are control animals with minimal to slight sinusoidal expression of IgM. **(Panel B1-B3)** Low dose treated animals with increased hepatic lobular expression of IgM. **(Panel C1-C3)** High dose treated animals with marked expression of IgM in regions of liver injury and regeneration. Some hepatocytes are positive for IgM (C3). **(Panel D1-D3)** None of the control animals express complement factor B. **(Panel E1-E3)** Low dose treated animals display induced expression of complement factor B in macrophages at a rim of a central vein (E1 and E2). Panel E3 is a high power field magnification depicting a cluster of macrophages and plasma cells with marked complement factor B expression. **(Panel F1- F3)** High dose treated animals with induced expression of complement factor B in macrophages and plasma cells. Activated complement factor B positive plasma cells and macrophages migrate from blood vessels to harmed hepatocytes. **(Panel G1)** Induced expression of factor B in harmed hepatocytes of a high dose treated animal. **(Panel G2** and **G3)** Migration of factor B positive macrophages and mast cells into sinusoidal walls and sheaths of central veins. The bar represents 10μm.

**Figure 7:**
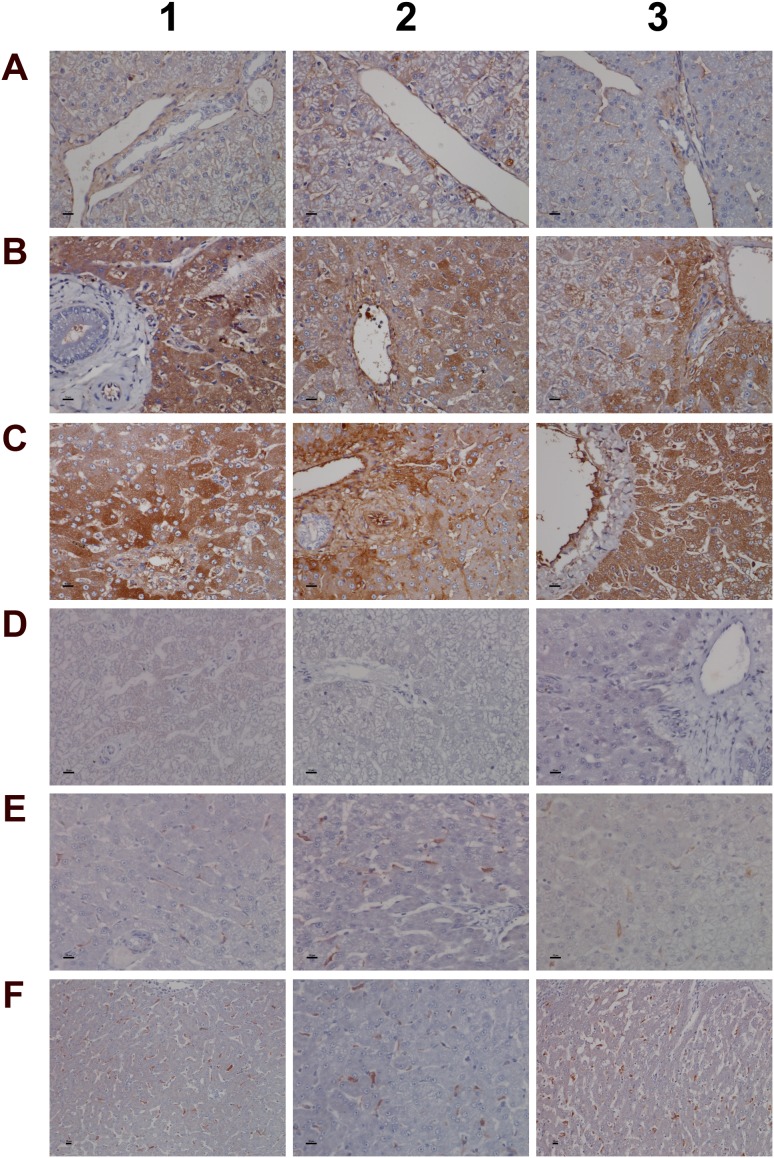
Immunohistochemistry staining of convertase C3 and the C1 inhibitor of the classical pathway in liver sections of control and diclofenac treated animals after daily dosing for 28 days **(Panel A1-A3)** Shown are control animals with no expression of convertase C3. **(Panel B1-B3)** Moderate to marked portal field convertase C3 expression in low dose treated animals. **(Panel C1-C3)** Marked convertase C3 expression in high dose treated animals. **(Panel D1-D3)** Shown are control animals with no expression of the C1 inhibitor protein. **(Panel E1-E3)** Low dose treatment of animals induced moderate expression of the C1 inhibitor in some but not all Kupffer cells. Note the sinusoidal enlarged macrophages. **(Panel F1-F3)** Marked proliferation of C1 inhibitor positive Kupffer cells in diclofenac induced inflammation. The bar represents 10μm.

**Figure 8:**
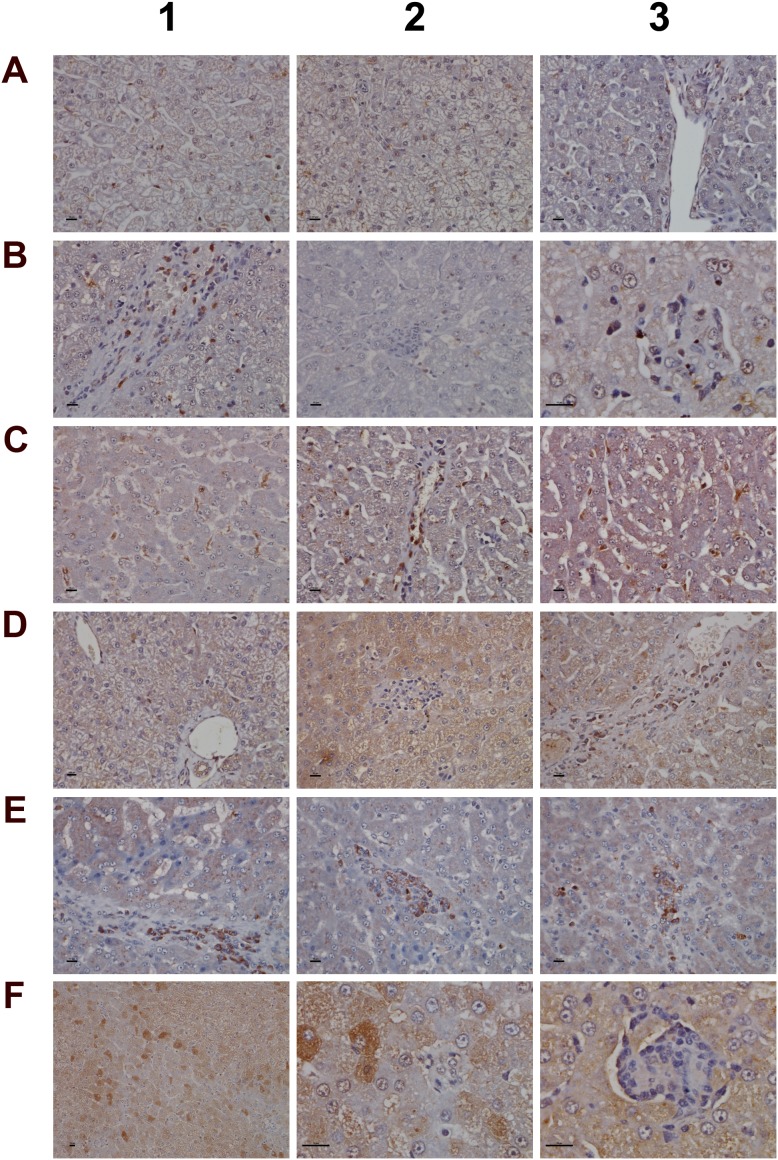
Immunohistochemistry staining of CD205 and CD74 in liver sections of control and diclofenac treated animals after daily dosing for 28 days **(Panel A1-A3)** Control animals do not express the CD205 protein. **(Panel B1)** Sinusoidal CD205 positive macrophages migrate into a vascular sheet of a low dose treated animal. **(Panel B2)** Slight expression of CD205 in Kupffer cells of low dose treated animals. Shown in the centre of the frame is an acute granulocytic/lymphocytic infiltrate close to a central vein. The inflammatory cell infiltrate is CD205 negative. **(Panel B3)** High power field magnification of a granuloma with CD205 positive resident Kupffer cells and migrated macrophages in a low dose treated animal. **(Panel C1)** Enlarged sinusoidal macrophages with marked CD205 expression of a high dose treated animal. **(Panel C2)** CD205 positive macrophages throughout the lobule and their accumulation in the area of the central vein (together with mast cells) of a high dose treated animal. **(Panel C3)** Activated and marked (lobular) CD205 positive Kupffer cell infiltrates and marked microvesicular steatosis of a high dose treated animal. **(Panel D1)** Shown are the portal triad and a central vein of a control animal with no expression of CD74. **(Panel D2)** Shown is a granuloma of a low dose treated animal. The macrophages do not express CD74. **(Panel D3)** CD74 positive cells gathered in the sheath of a central vein of a low dose treated animal. **(Panel E1-E3)** Clusters of CD74 positive cells in sheets of central veins (E1, E2) and within liver tissue (E3) of high dose treated animals. **(Panel F1** and **F2)** Hepatocytes with marked expression of CD74 of a low and high power field magnification. **(Panel F3)** High power field magnification of a granuloma with CD74 positive cells at the rim of the granuloma. The bar represents 10μm.

**Figure 9:**
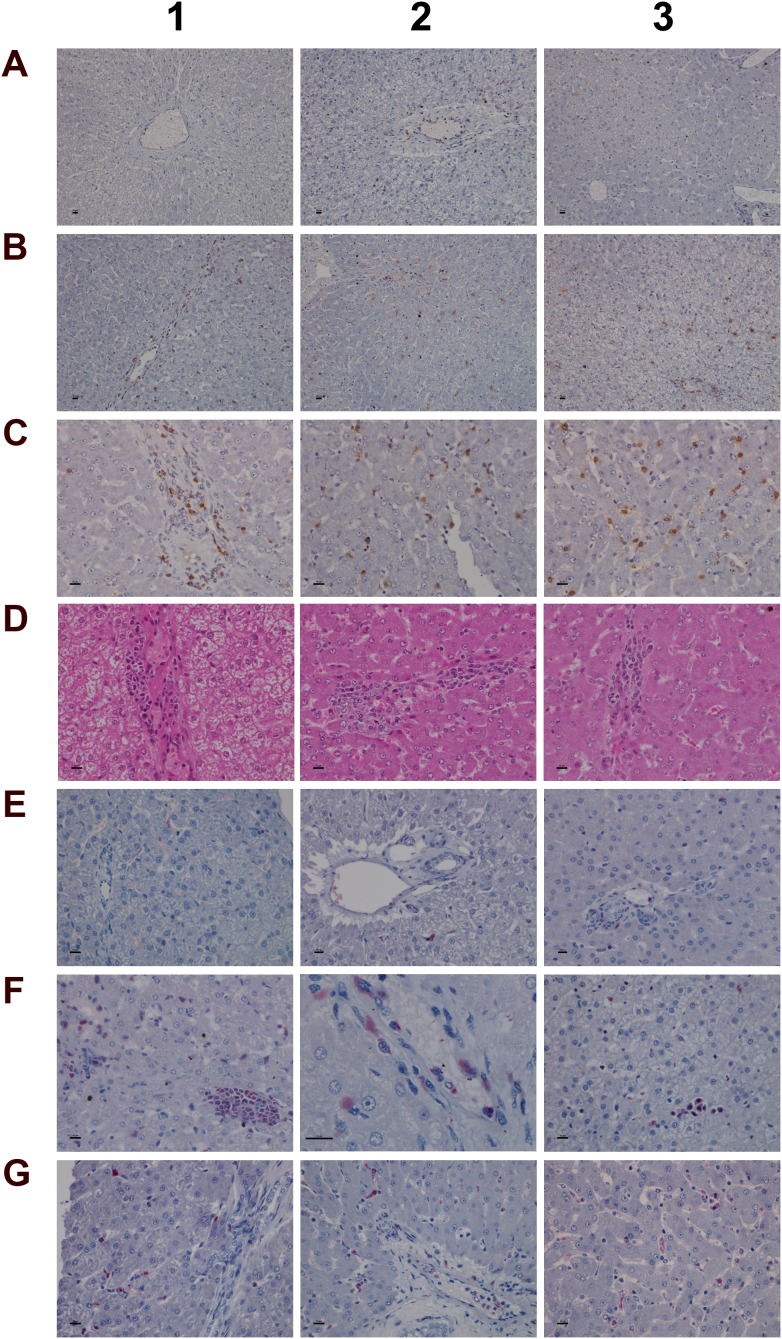
Immunohistochemistry staining of HIF1A and CAE in liver sections of control and diclofenac treated animals after daily dosing for 28 days **(Panel A1-A3)** Minimal HIF1A protein expression of resident mast cells. Like sentinel cells they occupy in smaller number the portal fields and the sinusoidal lining. **(Panel B1-B3)** Increased HIF1A expression in response to low dose treatment. The number of mast cells increased; note their localization in the intermediate lobular and centrolobular regions and around the central vein. **(Panel C1-C3)** Marked HIF1A expression of mast cell infiltrates and Kupffer cells in high dose treated animals; increased mast cell infiltrates which are now concentrated in and around the central vein. **(Panel D1-D3)** H&E staining depicting focal acute inflammatory infiltrates of central veins in low dose treated animals (D1), shifting to chronic inflammatory infiltrates in high dose treated animals (D2, D3) in association with the perivenously increased mast cells. **(Panel E1-E3)** CAE staining of control animals. Shown is the normal occurrence of periportal and portal field localized mast cells. **(Panel F1)** Centrolobular clusters of mast cells (left side) and perivenous granulocytic infiltrate of low dose treated animal. **(Panel F2)** Accumulated mast cells and a granulocyte in a central vein of a high dose treated animal. **(Panel F3)** A group of immature macrophages / small granuloma. **(Panel G1)** Periportal localized activated Kupffer cells with occasionally mast cell infiltrates in a low dose treated animal. **(Panel G2)** Migration of granulocytes, Kupffer and mast cells into hepatic parenchyma of a high dose treated animal. **(Panel G3)** Mixed type granulocyte and mast cell infiltrates of a high dose treated animal. Shown in the upper right quadrant is the proliferation and mitosis of presumably resident macrophages. The bar represents 10μm.

**Figure 10:**
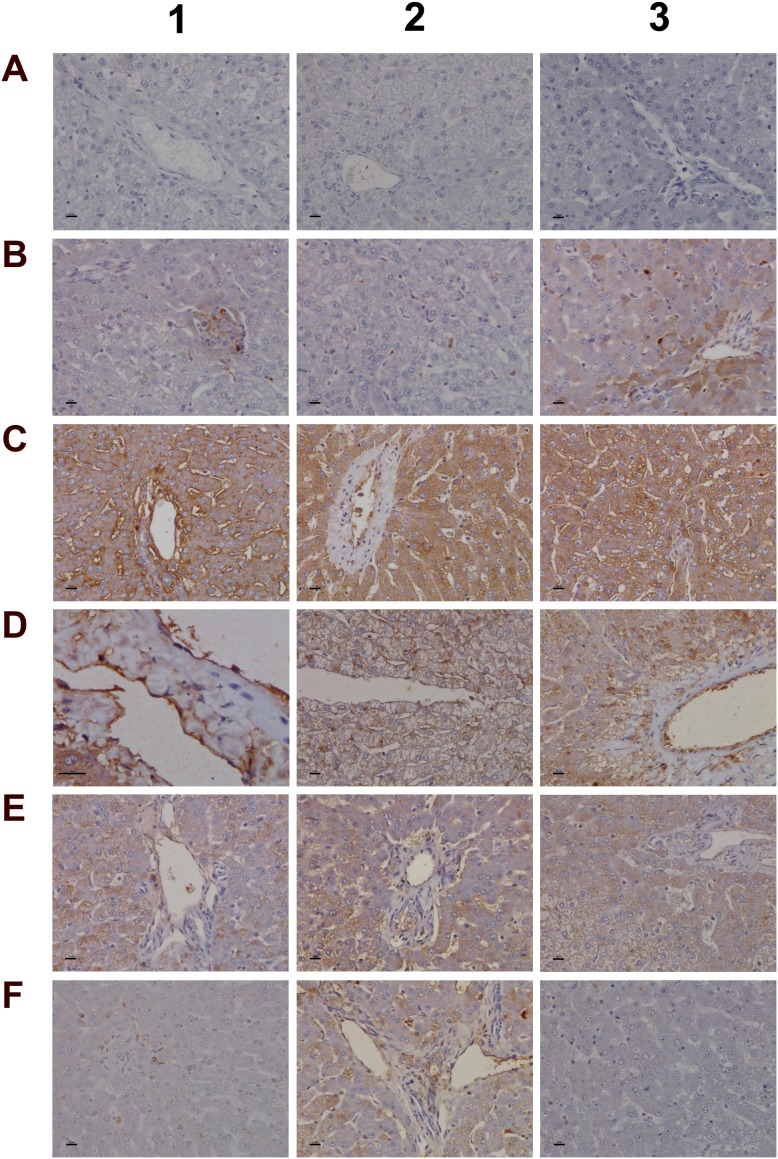
Immunohistochemistry staining of SAA1 and VCAM-1 in liver sections of control and diclofenac treated animals after daily dosing for 28 days **(Panel A1-A3)** Control animals do not express the SAA1 acute-phase protein. **(Panel B1)** Expression of SAA1 in a granuloma of aggregated macrophages of a low dose treated animal. **(Panel B2)** Expression of SAA1 in a subpopulation of macrophages of a low dose treated animal. **(Panel B3)** Moderate expression of SAA1 by harmed hepatocytes of a low dose treated animal. **(Panel C1-C3)** Marked hepatic and sinusoidal endothelial expression of SAA1in high dose treated animals. **(Panel D1)** High power field endothelium expression of VCAM-1 in a control animal. **(Panel D2 and D3)** Sinusoidal and endothelial expression of VCAM-1 of a central vein in control animals. **(Panel E1-E3)** Reduced sinusoidal and endothelial expression of VCAM-1 in low dose treated animals. **(Panel F1 and F2)** A subpopulation of VCAM-1 positive macrophages retained VCAM-1 expression. **(Panel F3)** Complete loss of sinusoidal and endothelial VCAM-1 expression in a marked steatotic liver of a high dose treated animal. The bar represents 10μm.

**Figure 11:**
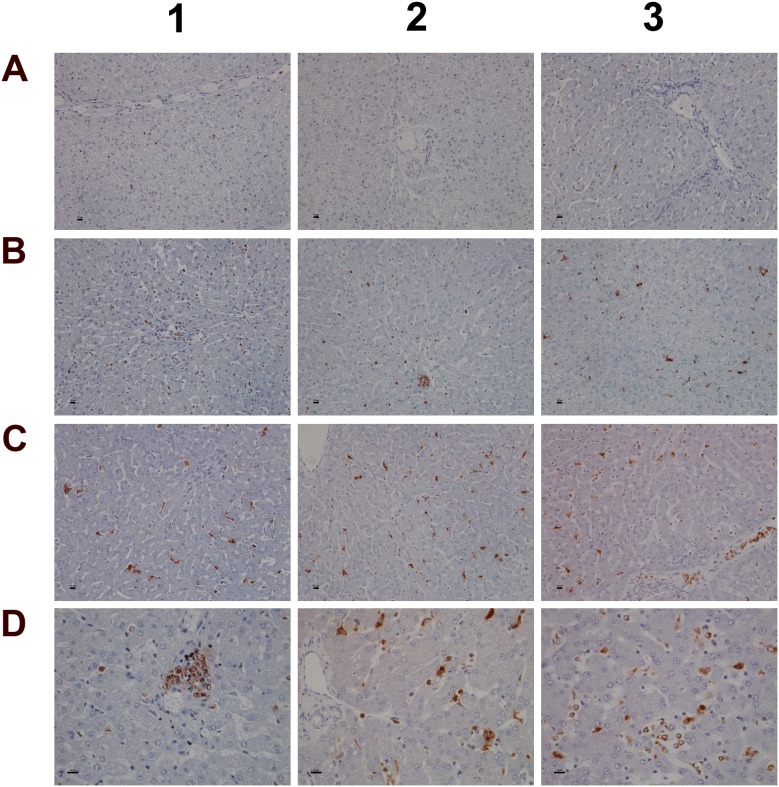
Immunohistochemistry staining of myeloperoxidase in liver sections of control and diclofenac treated animals after daily dosing for 28 days **(Panel A1-A3)** Control animals do not express MPO. **(Panel B1-B3)** Low dose treatment induced MPO expression in resident Kupffer cells, macrophages and granulocytes. Panel B2 depicts a granuloma with marked MPO expression. **(Panel C1 and C2)** Marked proliferation and activation of hepatic lobular MPO positive macrophages of high dose treated animals. **(Panel C3)** Migration of polarized macrophages and granulocytes from a portal field to regions of liver injury. **(Panel D1)** Marked MPO expression in a granuloma of a high dose treated animal. **(Panel D2)** Periportal mixed inflammatory cell infiltrate with marked MPO expression. Note the enlarged macrophages and the phagocytized neutrophils. **(Panel D3)** Hepatic lobular mixed inflammatory cell infiltrate consisting primarily of activated/polarized macrophages, newly infiltrating (immature) macrophages and granulocytes with marked MPO expression. The bar represents 10μm.

### Histopathology of the liver

Diclofenac treatment induced a range of lesions, namely drug induced steatosis and glycogen depletion to hallmark cellular stress and mitochondrial dysfunction, eosinophilic reactions, apoptosis, acute lobular hepatitis with sinusoidal and interstitial inflammatory cell infiltrates (Kupffer cells, immature macrophages, lymphocytes and granulocytes) as well as hepatocellular injury resulting in treatment related granulomas (Figure 5). Usually, the granulomas were demarcated by an edge of lymphocytes. The strong induction of IgG, IgM and acute phase reactants (see below) as well as induced Fc-receptor signaling are part of a coordinate response to trigger phagocytosis and inflammation and involve the classical and alternative pathway of the complement system. Furthermore, the marked mastocytosis indicate drug hypersensitivity reactions. With HD treated animals sinusoidal dilatation was observed that may result from the action of histamine, leukotrienes and prostaglandins and other mediators released from mast cells to support inflammatory cell infiltration.

Depicted in panel A1 is a control animal with normal parenchyma throughout the liver lobe. A predominantly monolayered trabecular structure and fluffy cytoplasm of hepatocytes can be seen. With LD treated animals (panel A2) and particularly in the periportal region (Zone 1) moderately increased liver regeneration was observed as defined by a denser basophilic cytoplasm of hepatocytes and a bilayered trabecular structure. With high dose treated animals marked regeneration was seen as evidenced by the extensive bilayered trabecula structure and basophilic cytoplasm of hepatocytes throughout the entire liver lobule. Panels B1 to B3 depict higher magnifications of control, LD and HD treated animals; with controls a fluffy cytoplasm with normal nuclei is seen (B1). LD treated animals presented moderately enlarged nuclei and nucleoli; regions of liver regeneration are particularly obvious in the periportal zone (lower right quadrant). Shown in B2 is the rim of a focal sinusoidal inflammatory infiltrate. With high dose treated animals (B3) marked liver regeneration was observed as denoted by bilayered hepatocyte trabeculi, dense cytoplasm, enlarged vesicular and prominent nucleoli. The sinusoids appeared widened with activated and enlarged Kupffer cells. There are also small granulomas (near the centre and the upper frame) with similar activated nuclei but swollen cytoplasm. The PAS staining of control, LD and HD treated animals is shown in panels C1-3. Dose dependent glycogen depletion was observed. Panel D1 is another HD treated animal with near complete depletion of glycogen stores and Supplementary Table 1 informs on glycogen synthesis and glucose metabolism regulated genes. The vacuolated hepatocytes hallmark drug induced microvesicular steatosis especially in the periportal and intermediate region, and the histology of panel D2 is another example of a high dose treated animal with a patchier pattern of hepatic microvesicular steatosis. D3 depicts the H&E stain of an LD treated animal and exemplifies the rare occurrence of focal microvesicular steatosis (below the portal field) within a periportal region of liver regeneration.

Diclofenac treatment induced programmed cell death; depicted in panel E1 and E2 are examples of fresh apoptotic cellular degenerations. A phagocytized apoptotic body within an enlarged Kupffer cell of a low dose treated animal is shown in panel E1. Apoptotic cells are mainly observed in the periportal/intermediate region (Zone 1/2). The HD treatment caused increased apoptotic activity. Disseminated single cell apoptosis was seen over the entire liver lobule, and panel E2 informs on early stage apoptotic cells that can be visualized by their eosinophilic cytoplasm and the notable pyknosis. Also documented is a phagocytized and partially degraded apoptotic cell within a Kupffer cell.

A transverse section of interstitial and sinusoidal inflammatory infiltrates is shown in panel E3. The mixed population of immature/migrating macrophages, Kupffer cells, granulocytes and lymphocytes caused arrosive damage, i.e. inflammation induced apoptosis as evidenced by groups of apoptotic cells. Occasionally mast cells are mixed within inflammatory infiltrates.

Shown in Figure 5 (panel F1 to G2) is the sequential consolidation of focal interstitial inflammatory infiltrates with final granuloma formation. At higher magnification a rounded focal interstitial/sinusoidal cellular infiltrate is observed that is intermingled with remnants of apoptotic cells (F1); panel F2 highlights initial granuloma formation in the liver of a LD treated animal; panel F3 exemplifies the consolidation of a granuloma. HD treatment caused diffuse alterations (panel G1) with a heterogeneous collection of predominantly polarized macrophages and mononuclear cell infiltrates as well as mixed granulocytes in a florid interstitial lesion. Depicted in panel G2 is the morphology of an infrequent but large granuloma in a steatotic liver of an HD animal. For comparison the portal field (PF) of a control animal is shown in panel G3. Tangential sections of PF highlight marked arteriolo-capillary and cholangiolar proliferation that appeared to be dose dependent (panel H1 (LD) & panel H2 (HD)). Shown in panel H3 is a transversely sectioned intact PF without inflammatory infiltrates of a HD treated animal. The proliferations occupied parts of the limiting plate and the periportal regions to likely initiate septal alterations as a result of increased hepatocyte regeneration.

To further examine architectural changes the Elastica van Gieson and the Gomori silver stain was employed. No evidence was obtained for early signs of fibrosis or enhanced deposition of extra cellular matrix into the sinusoids (data not shown).

### Histopathology of the kidney

Diclofenac treatment induced minimal to very slight renal mineralization in all LD and HD treated animals and minimal tubular regeneration in 2 out of 3 LD treated dogs. One animal each of the LD and HD group presented minimal focal nephropathy, and 2 animals of the HD group were scored with minimal tubular dilatation. No inflammatory cell infiltrates were observed in any of the treatment groups. Given the very minor changes no images are shown.

### Immunohistochemistry studies

A range of immunohistochemistry studies were performed. Figure 6 depicts IHC staining of IgM and complement factor B in control (A1-A3; D1-D3); low (B1-B3; E1-E3) and high dose (C1-C3; F1-F3, G1-G3) treated animals. A significant dose-dependent increase in IgM was observed, and next to its pronounced sinusoidal expression enhanced staining of IgM was particularly obvious in regions of injury. Some hepatocytes (panel C3) were positive for IgM to potentially indicate the synthesis of this immunoglobulin by harmed cells as had been observed by others [31, 32]. There is a clear evidence for IgM to “dress-up” harmed cells for complement mediated phagocytosis [33, 34]. We therefore hypothesize binding of IgM to danger-associated molecular patterns (DAMPs) expressed on the plasma membrane of harmed hepatocytes to endorse sterile inflammation, opsonization and Fcγ receptor signaling in support of phagocytosis. Moreover, induced expression of complement factor B is of critical importance. This protein is an essential component of the alternate complement pathway and a downstream effector of TLR signaling [35]. Together with C3b the catalytic subunit of factor B functions as the C3 convertase. As with the majority of proteins of the complement system factor B is primarily synthesized in hepatocytes [36]. Unlike controls (panel D1-D3) induced expression of factor B is observed in migrating monocytes, plasma cells (E3, F1) and macrophages (F1-F3) of low and high dose treated animals. Panel G1 exemplifies induced expression of factor B in harmed hepatocytes to possibly support mastocyte infiltration (panel G2) as was reported for the skin [37]. Infiltrates of factor B positive mast cells as well as migrating monocytes and macrophages are also depicted in panel G3 to once again highlight lobular inflammation. Frequently mast cells are associated with vascular endothelium (F2, F3). They are known to engage in complex interactions through binding to integrins and vascular adhesion molecules to support differentiation of mast cell precursors in the circulation and to instruct the endothelium in the orchestration of an inflammatory response [38].

We next evaluated the expression of convertase C3 and the C1 inhibitor of the classical pathway. As shown in Figure 7 and unlike controls (panel A1-A3) with minimal and primarily sinusoidal expression an unprecedented induction of the C3 protein was seen particular with harmed hepatocytes to support liver regeneration. Note, previous studies demonstrated the critical importance of C3 in liver regeneration after toxic injury with CCl4 [39]. Moreover, animals lacking C3 display impaired liver regeneration and develop acute liver failure after partial hepatectomy [40]. The expression of the C1 inhibitor was basically absent in control animals (panel D1-D3) but was dose dependently induced in low (E1-E3) and high dose (F1-F3) treated animals. Remarkably, expression of the C1 inhibitor is confined to subpopulations of macrophages with low and high expression of this protein to suggest differences in the activation states/polarization of macrophages and their sensor and effector functions in diclofenac induced inflammation. It is tempting to speculate that induction of the C1 inhibitor protein is an adaptive response to alleviate sinusoidal dilatation induced by inflammatory reactions [41].

To further investigate the nature of inflammatory cell infiltrates the expression of CD205 (DEC205) and of CD74 was investigated (Figure 8). Note, CD205 is a well characterized endocytic receptor that is highly expressed on dendritic cells and in different leukocyte populations [42]. Recently, it was shown that CD205 is also strongly expressed in Kupffer and sinusoidal endothelial cells [43] and functions as a recognition receptor for dying cells [44]. Diclofenac treatment caused a dose dependent induction of the CD205 protein in macrophages and monocytes/leukocytes. With control animals (panel A1-A3) expression of the protein was minimal or absent while the low (B1-B3) and high dose treatment (C1-C3) caused strong induction of the protein. Panel B2 also exemplifies CD205 negative inflammatory cell infiltrates (granulocytes, lymphocytes and migrating monocytes) close to a central vein. Shown in panel B1, C2 and C3 are enriched CD205 positive cell infiltrates in a vascular sheath, a central vein and sinusoids to sustain lobular inflammation. A distinct expression pattern is observed with some cells (monocytes/leucocytes) displaying strong CD205 plasma membrane expression while Kupffer cells tend to show strong intracellular expression. CD205 positive cells are enriched in granulomas (panel B3) and similar observations were made by others [45] with recent research suggesting CD205 expressing Kupffer cells to release interleukins to promote NKT cell activation [46].

Owing to its unique role in the folding and transport of MHC-molecules and its function as a receptor for the macrophage migratory inhibitory factor, i.e. a key factor to induce macrophage activation [47], expression of CD74 was investigated. The CD74 coding gene was strongly induced (>5-fold) in diclofenac treated animals (data given below) and the protein is expressed in lymphocytes, APCs, monocytes and macrophages [48]. Depicted in Figure 8D1 is a control animal with no expression of the protein. Diclofenac treatment induced expression of the protein and migration of CD74 positive cells (panel D3 and E1) to possibly prime cytotoxic responses. Shown in panel E2 and E3 are clusters of CD74 positive cells to facilitate antigen presentation and inflammation; panel E1-E2 and D3 exemplify mixed cell infiltrates in a central vein enriched with plasma cells with high CD74 expression. Frequently, CD74 positive mast cells associate with vascular endothelium and some hepatocytes express CD74 as well (panel F1 & F2). A similar staining of CD74 was reported in liver sections of patients with severe acute autoimmune hepatitis [49] though regenerative hepatocytes are negative for CD74 as shown in panel E1 and E2. Furthermore, at the rim of granulomas CD74 positive cells were observed (panel F3).

Given the important relationship between hypoxia and inflammation [50] the regulation of hypoxia inducible factor (HIF1A) was investigated (Figure 9). Unlike controls (panel A1-A3) a dose dependent approximately 4-fold increase in HIF1A transcript (data given below) and a highly significant HIF1A protein expression in low (panel B1-B3) and high dose (panel C1-C3) treated animals was observed. HIF1A was strongly expressed in mixed cell infiltrates, i.e. mast cells, macrophages and plasma cells in a vascular sheath (C1), and shown in panels C2 & C3 are sinusoidal mast cells with strongly induced HIF1A expression. Independent studies already confirmed induced HIF1A expression in activated mast cells [51]. With control animals typically a few mast cell are seen in the portal and periportal regions (A2 and A3). Their HIF1A protein expression is either minimal or absent. Diclofenac treatment caused marked mast cell infiltration to signify mastocytosis of the liver; a shift in localisation towards the lobular and the central vein is observed (panel B1-B3). Note the adhesion/margination of mast cells on an endothelial cell sheet of the central vein and adjacent to it an acute granulocytic infiltrate (B3).

Among its different functions HIF1A regulates expression of histidine decarboxylase (HDC) which catalyzes the formation of histamine from histidine. Though diclofenac treatment did not influence HDC gene expression (Supplementary Table 2) the transcript expression of the principal histamine inactivating enzymes, i.e. histamine N-methyltransferase (HMT) and amine oxidase (AOC) was significantly repressed to imply impaired histamine degradation. Furthermore, H&E (panel D) and CAE (panel E-G) staining revealed the treatment related mastocytosis and granulocytic infiltration. Depicted in panel D1-D3 are inflammatory infiltrates of the central vein associated with the treatment related mastocytosis. Panel D1 shows a sub-acute mixed inflammatory cell infiltrate (granulocytic, lymphocytic and immature macrophages) of a low dose treated animal whereas D2 to D3 exemplify the shift towards predominant plasmacytic cell infiltrates of high dose treated animals to imply a coordinate response to diclofenac induced injury with activated plasma cells differentiating into antibody producing lymphocytes.

The CAE staining of control animals is given in panel E1 to E3. A few mast cells reside in the portal/periportal region. With low and high dose treated animals (panel F1-F3) mast cell cluster and perivenous granulocytic infiltrates are observed. Depicted in panel F2 and F3 is the margination of mast cells on endothelium and the granulocyte in a central vein of a high dose treated animal as well as a group of immature macrophages. Mainly periportal localized activated Kupffer cells and occasionally mast cell infiltrates were observed in a low dose treated animal (G1) whereas high dose diclofenac treatment induced marked migration of granulocytes, Kupffer and mast cells into hepatic parenchyma (G2). Note the proliferation and mitosis of presumably resident macrophages in the upper right quadrant of panel G3. Collectively, the mixed granulocyte and mast cell infiltrates reinforce the notion of mast cell activation and granuloma formation in response to diclofenac treatment.

Among the acute phase reactants the expression of serum amyloid A (SAA) was investigated (Figure 10). This protein is typically induced by cytokines released from innate immune cells such as macrophages and monocytes. Diclofenac treatment prompted a clear dose related induction of SAA transcript (>60-fold, Table 1) and protein. Although control animals do not express SAA (panel 10A1-A3) strong expression of the protein was seen particularly in high dose treated animals (panel C1–C3). Panel 10B1 depicts a granuloma observed in a liver section of a low dose treated animal with expression of SAA in part by granuloma cells. Furthermore, hepatic lobular SAA expression by some hepatocytes and macrophages but not all macrophages is observed (panel B2-B3). Importantly, SAA influences macrophage differentiation into distinct subtypes [52], and the significant regulation of marker genes associated with M1 and M2 polarized macrophages in response to diclofenac treatment is discussed below.

**Table 1:** Commonly regulated hepatic DEGs after low- and high-dose diclofenac treatment

Gene symbol	Gene description	Fold change_LD (average) ± SD	Fold change_HD (average) ± SD
A2M	Alpha-2-macroglobulin	1.79±0.31	5.78±1.41
ABHD2	Abhydrolase domain containing 2	1.74±0.54	2.05±0.47
ACADSB	Acyl-CoA dehydrogenase, short/branched chain	-1.51±0.14	-2.3±1.01
ACSS2	Acyl-CoA synthetase short-chain family member 2	-1.58±0.32	-2.46±0.61
AGXT	Alanine-glyoxylate aminotransferase	-1.55±0.42	-2.25±3.58
ALDH9A1	Aldehyde dehydrogenase 9 family, member A1	-1.72±0.38	-3.73±1.37
C10H12orf23	Transmembrane protein 263	1.78±0.56	4.92±2.88
C11H9orf174	Chromosome 11 open reading frame, human C9orf174	-1.69±0.23	-2.31±0.59
CA3	Carbonic anhydrase III, muscle specific	-3.44±2.01	-24.38±1.86
CD9	CD9 molecule	1.78±0.59	3.2±1.44
CDADC1	Cytidine and dCMP deaminase domain containing 1	-1.64±0.5	2±0.74
CLDN10	Claudin 10	-1.64±0.4	-4.58±1.43
CLDN18	Claudin 18	1.76±0.38	-2.06±1.18
CYLD	Cylindromatosis (turban tumor syndrome)	1.73±0.46	2.12±0.91
CYP26A1	Cytochrome P450, family 26, subfamily A, polypeptide 1	-2.58±0.34	-4.44±0.25
CYP2B6	Cytochrome P450 2B11	-1.7±0.23	-8.63±1.07
DHCR7	7-dehydrocholesterol reductase	-1.96±0.15	-2.08±0.91
EGR1	Early growth response 1	2.56±0.73	6.6±3.01
ELOVL2	ELOVL fatty acid elongase 2	4.01±1.71	9.75±3.04
EPSTI1	Epithelial stromal interaction 1	-1.54±0.22	-2.28±1.09
FABP7	Fatty acid binding protein 7	1.88±0.19	2.34±0.98
FAM159A	Family with sequence similarity 159, member A	1.56±0.26	2.24±0.14
FDPS	Farnesyl diphosphate synthase	-1.5±0.24	-2.68±0.95
GABPA	GA binding protein transcription factor, alpha subunit 60kDa	1.52±0.14	3.94±1.97
GK	Glycerol kinase	1.58±0.36	3.23±1.09
GPT	Glutamic-pyruvate transaminase	-1.5±0.21	-4.99±2.94
GSTA4	Glutathione S-transferase alpha 4	-1.54±0.09	-5.29±1.8
HAMP	Hepcidin antimicrobial peptide	-4.99±1.34	-4.12±1.98
HMGN2	Non-histone chromosomal protein HMG-17	-1.56±0.45	-1.5±1.92
HSD11B1L	Hydroxysteroid (11-beta) dehydrogenase 1-like	-1.57±0.4	-2.03±0.14
IDI1	Isopentenyl-diphosphate delta isomerase 1	-1.59±0.23	-2.08±0.48
IFI35	Interferon-induced protein 35	-1.56±0.16	-3.67±3.48
IL1B	Interleukin 1, beta	1.82±0.32	2.02±0.16
IL33	Interleukin 33	-1.65±0.28	-4.17±1.24
ITIH3	Inter-alpha-trypsin inhibitor heavy chain 3	1.52±0.28	2.13±1.32
LBP	Lipopolysaccharide binding protein	3.21±1.74	32.93±2.07
MFSD2A	Major facilitator superfamily domain containing 2A	2.06±0.41	2.26±0.6
MRPL22	Mitochondrial ribosomal protein L22	1.55±0.15	2.02±0.49
NEIL3	Nei endonuclease VIII-like 3	-2.15±0.44	-3.59±0.85
OAS1	2'-5'-oligoadenylate synthetase 1, 40/46kDa	-1.51±0.18	-2.75±1.37
OXCT1	3-oxoacid CoA transferase 1	-1.83±1.39	-6.24±3.46
PKLR	Pyruvate kinase, liver and RBC	-1.78±0.46	-8.22±1.5
PLIN2	Perilipin 2	1.65±0.26	8.11±4.19
RHPN2	Rhophilin-2	-1.52±0.14	-3.62±2.28
S100A8	S100 calcium binding protein A8	1.57±0.1	9.77±2.02
SAA1	Serum amyloid A1	3.09±1.08	61.77±2.97
SFTPD	Surfactant protein D	1.51±0.21	3.74±1.02
SLC16A4	Solute carrier family 16, member 4	-1.62±0.47	-3.26±1.12
THBS1	Thrombospondin 1	1.74±0.5	2.14±0.58
TMEM178	Transmembrane protein 178A	-1.51±0.16	-2.87±0.81
TMEM43	Transmembrane protein 43	1.65±0.21	3.66±1.56
TXNDC5	Thioredoxin domain containing 5 (endoplasmic reticulum)	1.52±0.32	4.85±2.4
WIPI1	WD repeat domain, phosphoinositide interacting 1	1.67±0.24	6.13±1.06

Dogs were given 1mg/kg or 3 mg/kg once daily for 28 days. Whole genome expression profiling was performed and DEGs were calculated based on the criteria fold change >1.5 and a p-value <0.05. Collectively, 53 genes were regulated in common when low and high dose treatment groups were compared.

Additionally, expression of VCAM-1 was considered. This cell adhesion molecule plays an essential role in leukocyte recruitment and is typically induced in inflammation [53]. Shown in panel D1 is a high power field magnification of VCAM-1 expression of vascular endothelium as well as the sinusoidal (panel D2) and endothelial expression on a central vein (panel D3) of control animals. Diclofenac treatment elicited a dose dependent reduction of VCAM-1 protein expression in low (panel E1-E3) and high dose (F1-F3) treated animals; however hepatic gene expression of VCAM-1 was unchanged. The findings agree with earlier reports obtained in HUVEC cells whereby diclofenac treatment inhibited expression of the endothelial cell adhesion molecule VCAM-1, ICAM-1 and E-selectin [54]. Similar results were also reported for ibuprofen [55] and this NSAID was shown to inhibit leukocyte migration through endothelial cell monolayers [56]. Notwithstanding a distinct subpopulation of macrophages/monocytes retained VCAM-1 expression after diclofenac treatment (panel F1/F2). It is tempting to speculate that VCAM-1 positive macrophages retain hematopoietic stem cell properties and facilitate HSC trafficking to the liver to sustain extramedullary myelopoiesis and to block macrophage maturation as reported for spleen VCAM-1^+^ macrophages [57]. Moreover, silencing of VCAM-1 or M-CSFR in ApoE -/- mice limited inflammation and reduced myeloid cell numbers in atherosclerotic plaques [57] though in the present study M-CSFR was significantly up-regulated at the high dose regimen (as discussed below).

Given that myeloperoxidase (MPO) is a critical effector of inflammation and abundantly expressed in neutrophils, monocytes and macrophages [58] its regulation in diclofenac treated animals was investigated. Moreover, diclofenac can be metabolized to reactive metabolites by MPO [59] which prompted our interest to investigate its expression. As depicted in Figure 11 (panel A1-A3) expression of MPO was basically absent in control animals. Conversely, a clear dose dependent induction of MPO in low (panel B1-B3) and high dose (C1-D3) treated animals is seen. Panel B2 depicts a small granuloma with marked expression of MPO while panel C2 and C3 document hepatic lobular proliferation of polarized macrophages and the migration of activated neutrophils and monocytes from a portal field to regions of liver injury. Furthermore, a granulomatous inflammatory reaction adjacent to a central vein and the periportal inflammation of highly activated and polarized macrophages is shown in panels D1 and D2, respectively. Within the cytoplasm of macrophages prominent phagosomes are noted, and the activity of macrophages may extend to the phagocytosis of neutrophils to support resolution of drug induced inflammation as had been demonstrated in the past [60, 61]. Importantly, the blood smear test evidenced neutrophilic leukocytosis (Figure 3) in diclofenac treated animals and the phagocytosis of neutrophils will limit inflammation. Shown in panel D3 is the marked inflammatory cell infiltrate of neutrophils and macrophages with cytoplasmic and granular positivity for MPO; note the concentric plasma membrane MPO staining of granulocytes.

**Figure 12:**
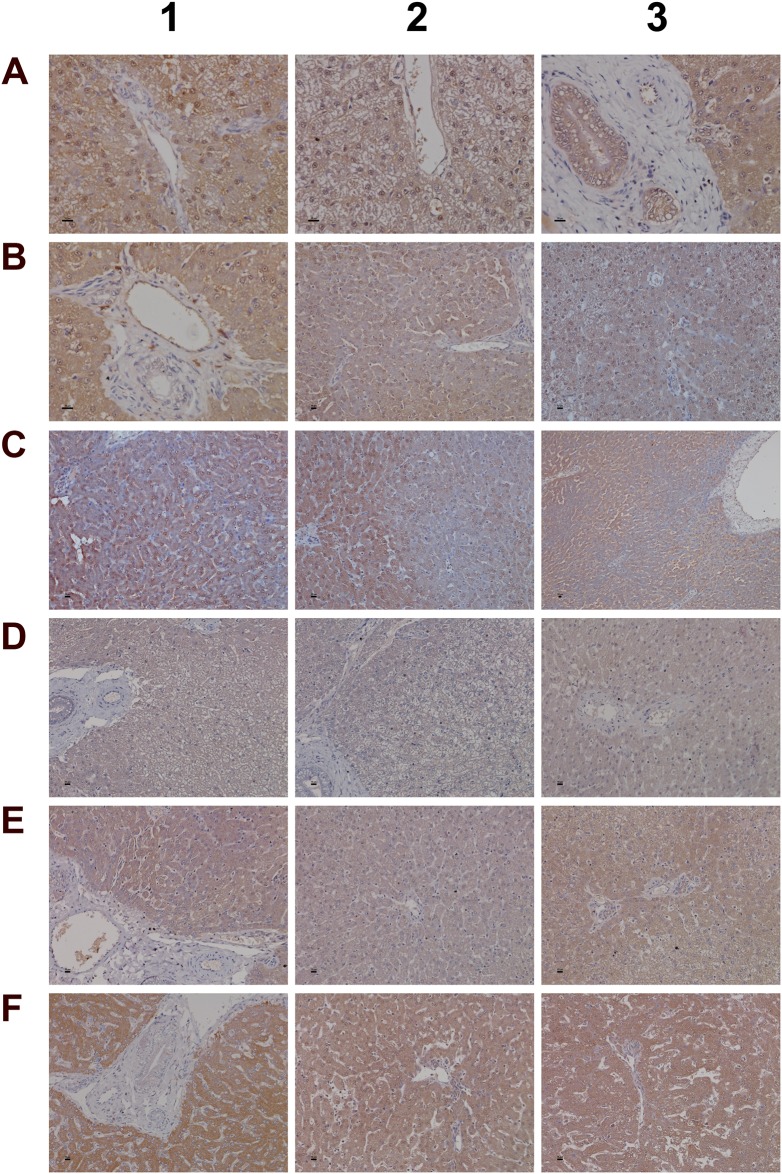
Immunohistochemistry staining of superoxide dismutase SOD1 and SOD2 in liver sections of control and diclofenac treated animals after daily dosing for 28 days **(Panel A1-A3)** Hepatocytes of control animals express abundantly SOD1. Shown in panel A3 is bile duct epithelium also positive for SOD1. **(Panel B1-B3)** A slight to moderate reduced SOD1 expression is observed in low dose treated animals. Some portal macrophages display strong SOD1 expression. Note the patchier periportal (B2) and hepatic lobular expression of SOD1 (B3). **(Panel C1-C3)** A moderate to marked reduction in SOD1 expression is observed with high dose treated animals. **(Panel D1-D3)** Minimal SOD2 expression in hepatocytes of control animals. **(Panel E1-E3)** Portal (E1), lobular (E2) and periportal (E3) hepatic SOD2 expression is increased in low dose treated animals. **(Panel F1-F3)** Marked portal, lobular and periportal hepatic SOD2 expression in high dose treated animals. The bar represents 10μm.

The further explore the link between drug induced inflammation, myeloperoxidase activity and oxidative stress expression of the superoxide dismutase SOD1 and SOD2 was investigated. As shown in Figure 12 and unlike controls (panel A1-A3) a dose dependent reduction in the expression of the cytosolic Cu-Zn SOD1 was observed in low (B1-B3) and high dose (C1-C3) treated animals. Alike, SOD1 transcript expression was significantly reduced to about 40% of control values to evidence impaired superoxide anion radical detoxification induced by diclofenac treatment (data given below). Conversely and when compared to controls (D1-D3) expression of the mitochondrial Mn-SOD2 increased from low (E1-E3) to high dose treated animals (panel F1-F3), and a similar nearly 5-fold induction of the SOD2 transcript was determined. Some of the bile duct epithelia were also positive for SOD1 and SOD2 as shown in liver sections of panels C3 and E3, respectively.

**Figure 13:**
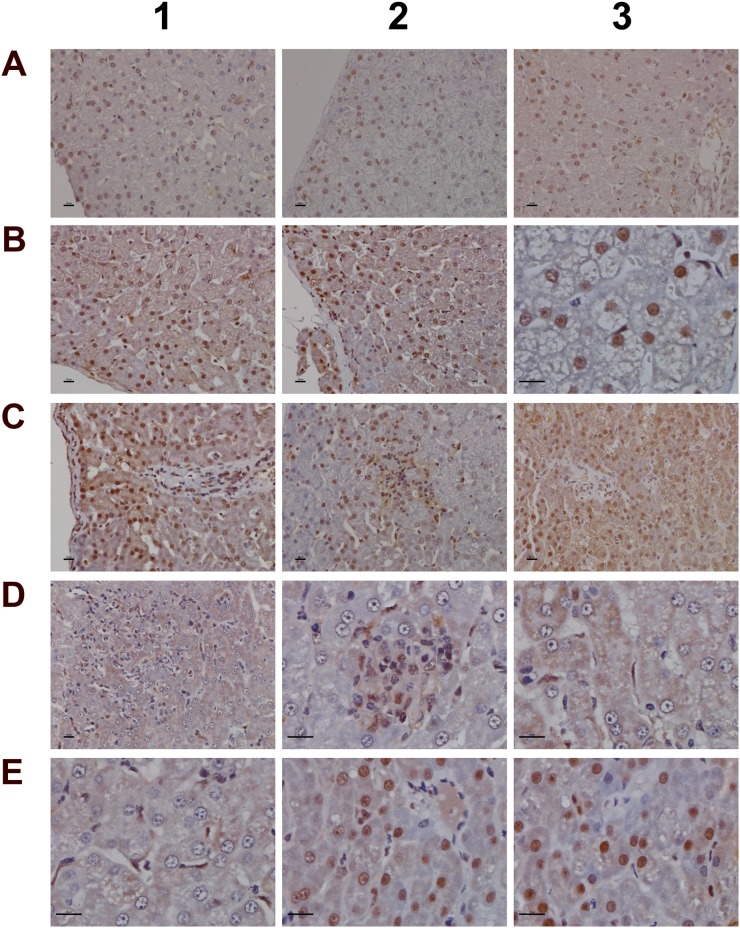
Immunohistochemistry staining of the Krüppel-like factor 6 in liver sections of control and diclofenac treated animals after daily dosing for 28 days **(Panel A1-A3)** Shown are control animals with minimal to slight nuclear KLF6 expression. **(Panel B1-B3)** Increased nuclear KLF6 staining of low dose treated animals. Panel B3 is a high power field magnification with intense nuclear KLF6 expression. **(Panel C1)** High dose treatment induced marked nuclear expression of KLF6; additionally harmed hepatocytes show cytosolic KLF6 expression. Note expression of KLF6 in monocytes, macrophages and lymphocytes in a blood vessel and their likely migration into liver parenchyma. **(Panel C2)** High dose treatment. Initial granuloma formation with mixed inflammatory cell infiltrates; marked KLF6 expression in resident Kupffer cells and monocytes. **(Panel C3)** Marked nuclear and cytosolic expression of KLF6 in a fatty liver of a high dose treated animal. **(Panel D1)** Acute hepatic lobular inflammation with marked mixed inflammatory cell infiltrates in a high dose treated animal. KLF6 positive resident and immature immigrant macrophages first at the periphery of the arrosive lesion. **(Panel D2)** High power field magnification of a granuloma with distinct subpopulation of KLF6 expressing macrophages and infiltrating monocytes in a high dose treated animal. **(Panel D3)** High power field magnification of bilayered trabeculae and steatotic hepatocytes; distinct subpopulations of polarized macrophages with low and high KLF6 expression. **(Panel E1-E3)** High power field magnification of prominent nuclear and moderate cytosolic KLF6 expression of high dose treated animals. Note the distinct macrophage subpopulations expressing KLF6. The bar represents 10μm.

For its importance in regulating inflammatory macrophage polarization [62] and its critical function in the control of gene expression immunohistochemistry of the transcription factor KLF6 was performed. Shown in Figure 13 panel A1-A3 are control animals with minimal to slight nuclear KLF6 expression. A dose dependent increase in the nuclear (B1-B3) and at the high dose (C1-C3) additional cytosolic expression of KLF6 was seen. Panel D1 depicts hepatic lobular inflammation in a high dose treated animal with marked inflammatory cell infiltrates (granulocytes, migrating monocytes, lymphocytes and macrophages). The granulocytes and cytotoxic lymphocytes are KLF6 negative; however, some Kupffer cells and migrating monocytes are KLF6 positive. Shown in panel D2 is a granuloma with a distinct subpopulation of macrophages expressing KLF6. Further examples are given in panel D3 and E1 with irregular, mainly bilayered trabeculae and distinct population of KLF6 positive Kupffer cells. The high power field view of panels E2 and E3 depicts prominent nuclear and some cytosolic expression of KLF6. The panels D1, C2 and D2 imply a sequence of granuloma formation with an acute inflammatory infiltrate followed by an initial granuloma formation and its subsequent consolidation.

### Genomic responses in liver and kidney after diclofenac treatment

Given that diclofenac inhibits cyclooxygenase activity the expression of the coding genes was investigated. We observed significant 5- and 3-fold induction of COX2 but a 50% and 40% reduction in COX1 expression in liver and kidney, respectively after repeated treatment for 28 days (Supplementary Table 3).

**Figure 14:**
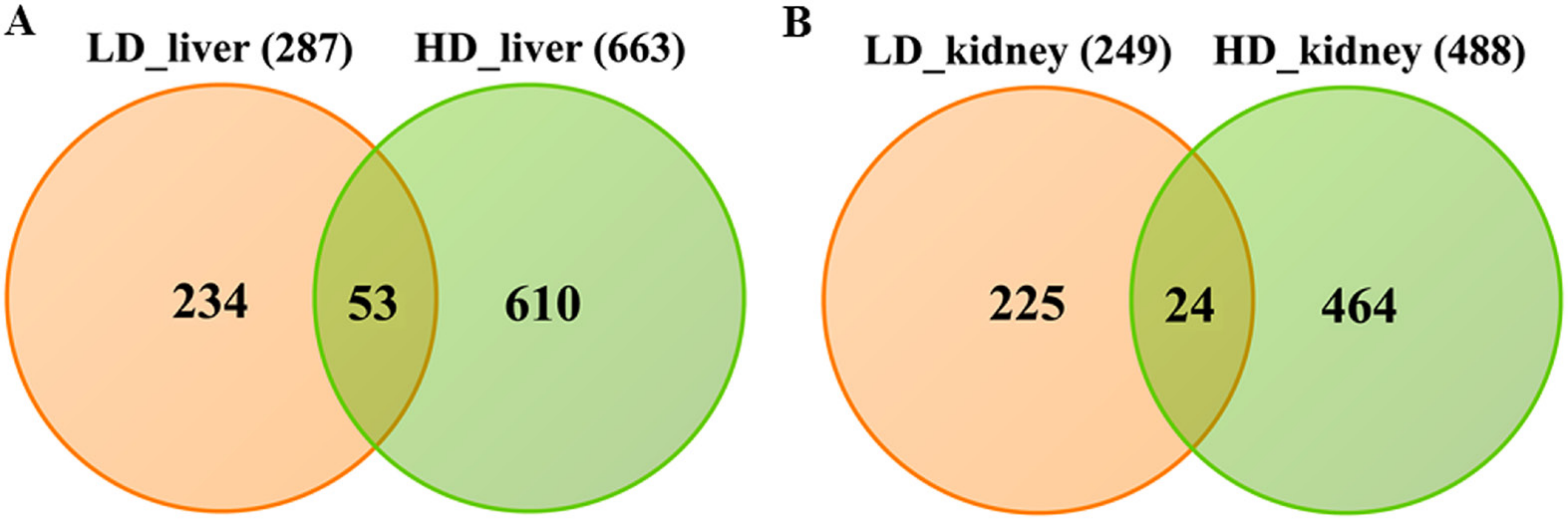
Differentially expressed genes after low and high dose diclofenac treatment **(Panel A)** Venn diagram of hepatic DEGs after low (1 mg/kg/day) and high dose (3 mg/kg/day) treatment for 28 days. A total of 53 genes are regulated in common. **(Panel B)** Venn diagram of kidney DEGs after low and high dose treatment. A total of 249 and 488 genes were significantly regulated in low and high dose treatment, of which 24 were commonly regulated.

**Figure 15:**
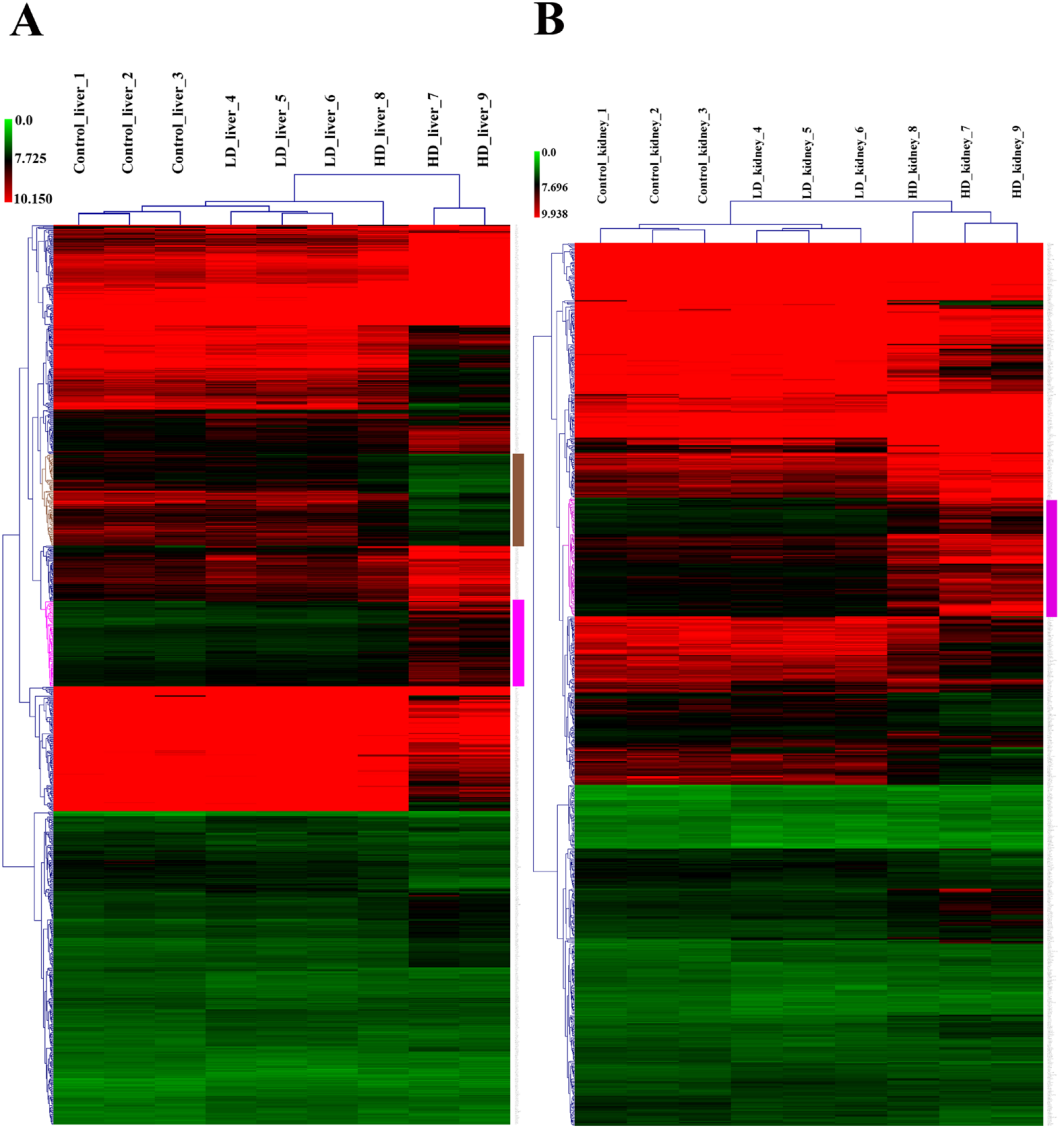
Heatmap of differentially expressed genes in liver and kidney of diclofenac treated dogs Shown is the hierarchical gene clustering of DEGs in liver **(Panel A)** and kidney **(Panel B)**. The heatmap was generated with the MeV software and an average-linkage hierarchical clustering with Euclidean distance was applied. Depicted are the signal intensity values of regulated DEGs. The low and high dose treatment groups were clearly segregated from the controls. The magenta colored dendrogram depicts clusters of DEGs involved in the biological processes stress, immune responses and cell death. Likewise, the brown colored dendrogram shows clustering of metabolic genes which were repressed in expression after diclofenac treatment.

Whole genome gene expression profiling revealed 287 (167 up- and 120 down-) and 663 (328 up- and 335 down-) genes as significantly regulated in the liver of low and high dose treated animals with 53 genes commonly regulated among both treatments (Figure 14A and Table 1). Alike, diclofenac treatment caused 249 (106 up- and 143 down-) and 488 (255 up- and 233 down) DEGs in kidney of low and high dose treated animals of which 24 were in common (Figure 14B and Table 2). By applying the average-linkage hierarchical clustering algorithm with Euclidian distance, heatmaps for DEGs were generated. The dendogram of both liver and kidney regulated genes highlights a distinct expression pattern (control, low dose and high dose) (Figure 15A and 15B) with clusters of up-regulated genes coding for stress and immune response and cytotoxicity (cell death and apoptosis). Conversely, genes coding for lipid, hexose and protein metabolism were frequently repressed in expression in low and high dose treated animals. Tables 1 and 2 provides a summary of dose related gene regulations. As an example the lipopolysaccharide binding protein and serum amyloid A1 were induced by 3-, 33- and 3- and 62-fold, respectively, in liver of low and high dose treated animals. A similar dose related change in gene expression was observed for the kidney though the regulation of genes differed when two organs were compared (Table 2).

**Table 2:** Commonly regulated genes in kidney after low and high-dose diclofenac treatment

Gene Symbol	Gene description	Fold change_LD (average±SD)	Fold change_HD (average±SD)
ACMSD	Aminocarboxymuconate semialdehyde decarboxylase	1.58±0.18	-2.21±1.04
ARMC3	Armadillo repeat containing 3	-1.78±0.73	-2.41±0.96
BCL2A1	BCL2-related protein A1	1.54±0.32	2.36±0.95
CLK1	CDC-like kinase 1	-1.62±0.3	-1.9±0.33
DMGDH	Dimethylglycine dehydrogenase	-1.54±0.76	-2.32±0.68
DNASE1	Deoxyribonuclease I	-1.92±0.81	-3.71±2.34
DPT	Dermatopontin	-1.5±0.43	-2.27±0.58
EGFLAM	EGF-like, fibronectin type III and laminin G domains	-1.96±0.53	-2.76±2.81
ELSPBP1	Epididymal sperm binding protein 1	-1.56±0.11	1.94±0.31
ENPP6	Ectonucleotide pyrophosphatase / phosphodiesterase 6	-2.08±1.59	-2.38±2.43
FGGY	FGGY carbohydrate kinase domain containing	-2.19±0.24	-2.18±0.13
FMO1	Flavin containing monooxygenase 1	2.23±0.76	-2.33±0.54
KLHDC1	Kelch domain containing 1	-1.52±0.18	-1.87±0.21
RBP2	Retinol binding protein 2	1.79±0.14	-8.79±2.36
RPS24	Ribosomal protein S24	1.88±0.25	1.87±0.14
S100A10	S100 calcium binding protein A10	1.6±0.46	2.82±1.44
SDC4	Syndecan 4	1.79±0.14	2.58±0.97
SLC12A2	Solute carrier family 12, member 2	2.1±0.24	5.09±1.05
SLC5A12	Solute carrier family 5, member 12	-1.54±0.06	-2.69±3.29
SLC5A8	Solute carrier family 5, member 8	-1.86±0.22	-2.5±0.99
SNAP25	Synaptosome associated protein 25	1.54±0.15	1.88±0.66
UBE2J1	Ubiquitin Conjugating Enzyme E2 J1	-1.52±0.2	2.29±0.64
ZFAND5	Zinc finger, AN1-type domain 5	1.66±0.08	2.03±0.53
ZFP92	Zinc finger protein 92	1.6±0.18	1.88±0.35

A total of 24 statistically significant DEGs were regulated in common amongst low and high dose diclofenac treatment.

### Functional enrichment analysis

About 92% of DEGs could be mapped to the human genome and were used for enrichment analysis. DEGs were submitted to the GO, KEGG and BioCarta databases to define over-represented ontology terms, and the p-values given in Table 3 and 4 were obtained using a hypergeometric testing strategy. Specifically, the immune, inflammation, acute phase and stress response were highly regulated biological processes and involved 225 DEGs (Supplementary Table 3). Unlike the low dose for which cell cycle was a significantly enriched term the high dose treatments alerted to cell death and involved genes coding for redox stress and glutathione homeostasis (GCL, GGT, GSTs, GPX, SOD1, SOD2, Thioredoxins). Alike, cytokine stimulus, xenobiotic metabolism and defence were significantly enriched terms as was glucose and lipid metabolism, PPAR signaling and fatty acid oxidation (Table 3). Note, histopathology of diclofenac treated animals revealed hepatic steatosis and Table 5 compiles drug induced steatosis regulated genes. For instance the lipid droplet associated PLIN2 and the very long chain fatty acids protein 2 were induced by 8- and 10-fold; in fact >40 genes coding for lipogenesis, lipid transport, lipid droplet growth ER stress and fatty acid oxidation were affected to signify major changes in lipid homeostasis. The data also revealed an approximate 4-fold induction of FGF21, and this growth factor is known to ameliorate hepatic steatosis. Supplementary Figures 1-4 summarize significantly enriched ontology terms by considering molecular, cellular and biological functions of DEGs regulated in liver and kidney.

**Table 3:** Enriched biological processes and pathways in liver after diclofenac treatment

GO ID	Biological process	Low dose		High dose	
		No of genes (% associated genes with term)	P-value	No of genes (% associated genes with term)	P-value
GO:0006955	Immune response	22 (1.41%)	2.95E-04	47 (3.01%)	6.40E-05
GO:0006950	Response to stress	54 (1.51%)	9.16E-05	145 (4.04%)	6.54E-14
GO:0006954	Inflammatory response	8 (1.21%)	0.03136	25 (3.78%)	1.01E-04
GO:0006953	Acute phase response	4 (8.51%)	6.51E-06	8 (17.02%)	3.56E-08
GO:0055114	Oxidation-reduction process	21 (1.92%)	0.00771	86 (7.86%)	2.24E-18
GO:0034097	Response to cytokine stimulus	13 (1.63%)	0.01576	37 (4.64%)	7.94E-08
GO:0001666	Response to hypoxia	9 (3.19%)	0.03945	20 (7.09%)	0.00293
GO:0006952	Defense response	23 (1.58%)	2.21E-04	57 (3.90%)	1.78E-06
GO:0007049	Cell cycle	22 (1.28%)	0.00354	9 (0.52%)	0.00799
GO:0010941	Regulation of cell death			57 (3.77%)	0.00928
GO:0006805	Xenobiotic metabolic process	7 (7.37%)	0.00927	27 (28.42%)	1.30E-15
GO:0006629	Lipid metabolic process	27 (2.02%)	0.00297	86(6.43%)	1.25E-18
GO:0019318	Hexose metabolic process	8 (3.33%)	0.00884	20 (8.33%)	2.03E-06
GO:0006096	Glycolysis	4 (5.33%)	0.00345	5 (6.67%)	0.00902
GO:0035357	PPAR signaling pathway	4 (5.56%)	0.03425	14 (19.44%)	0.000003
GO:0038061	NF-kB signaling	3 (2.73%)	0.04808	7 (6.36%)	0.114357
GO:0006635	Fatty acid beta oxidation	4 (5.63%)	0.00157	9 (12.68%)	9.21E-05
GO:0038093	Fc receptor signaling pathway			7 (2.88%)	0.00851
GO:0000165	MAPK cascade	7 (0.81%)	0.02193	7 (0.81%)	0.676457
hsa04151	PI3K-AKT signaling pathway			15 (4.40%)	0.671795
hsa04152	AMPK signaling pathway			8 (6.45%)	0.164278

Gene ontologies were analyzed with the GeneXplain and ClueGO database; statistically significantly enriched biological processes were considered at a p-value < 0.05. The percentage of genes associated with biological terms and pathways were calculated with the AmiGO 2 software (http://amigo.geneontology.org/amigo/landing) and KEGG repository data entries.

**Table 4:** Enriched biological processes and pathways in kidney after diclofenac treatment

GO ID	Biological process	Low dose		High dose	
		No of genes (% associated genes with term)	P-value	No of genes (% associated genes with term)	P-value
GO:0006955	Immune response			41 (2.63%)	6.66E-07
GO:0006959	Humoral immune response	4 (1.76%)	0.04546	13 (5.73%)	1.50E-07
GO:0006954	Inflammatory response			19 (2.87%)	3.43E-04
GO:0006950	Response to stress			111 (3.10%)	3.12E-12
GO:0055114	Oxidation-reduction process	16 (1.46%)	0.04881	46 (4.20%)	2.12E-07
GO:0001666	Response to hypoxia			16 (5.67%)	8.86E-04
GO:0034097	Response to cytokine stimulus			36 (4.52%))	1.63E-16
GO:0019221	Cytokine-mediated signaling pathway			16 (2.93%)	5.31E-04
GO:0009611	Response to wounding			32 (5.07%)	4.85E-08
GO:0006805	Xenobiotic metabolic process			11 (11.58%)	1.04E-04
GO:0000075	Cell cycle checkpoint	6 (2.70%)	0.03000	12 (5.41%)	0.00852
GO:0051726	Regulation of cell cycle	16 (1.58%)	0.01735	16 (1.58%)	4.96E-04
GO:0010941	Regulation of cell death			54 (3.57%)	2.65E-05
GO:0097190	Apoptotic signalling pathway			16 (2.77%)	5.97E-04
GO:0006952	Defense response			52 (3.56%)	7.89E-08
GO:0006629	Lipid metabolic process	20 (1.49%)	0.03631	67 (5.01%)	5.95E-16
GO:0006635	Fatty acid β-oxidation	3 (4.22%)	0.02734	3 (4.23%)	0.00923
GO:0019318	Hexose metabolic process			15 (6.25%)	5.56E-05
GO:0019538	Protein metabolic process			91 (1.65%)	0.02606
GO:0055085	Transmembrane transport	19 (1.34%)	0.01840	47 (3.31%)	2.18E-08
GO:0006956	Complement activation			12 (10.08%)	4.33E-13
GO:0035357	PPAR signaling pathway	4 (5.56%)	0.01541	13 (18.06%)	3.9000E-07
hsa04910	Insulin signaling pathway			9 (6.43%)	0.01732371
hsa04066	HIF-1 signaling pathway			9 (8.74%)	0.00341
hsa04920	Adipocytokine signaling pathway	4 (5.71%)	0.00990	11 (15.71%)	4.4600E-06
hsa04151	PI3K-AKT signaling pathway			19 (5.57%)	0.00717776

Gene ontologies were analyzed with the GeneXplain and ClueGO database; statistically significantly enriched biological processes were considered at a p-value < 0.05. The percentage of genes associated with biological terms and pathways were calculated with the AmiGO 2 software (http://amigo.geneontology.org/amigo/landing) and KEGG repository data entries.

**Table 5:** Drug induced steatosis regulated genes in liver and kidney after diclofenac treatment

Gene	Gene description	Liver		Kidney	
		LD	HD	LD	HD
		Fold change (average)±SD			
**Lipogenesis**					
ACACB	Acetyl-CoA carboxylase beta	1.65±0.25^*^	-1.37±0.02	-1.02±0.3	-3.82±1.85^*^
ELOVL2	ELOVL fatty acid elongase 2	4.01±1.67^*^	9.75±3.04^*^	1.23±0.1	-2.03±1.09^*^
FADS1	Fatty acid desaturase 1	-1.07±0.05	1.04±0.07	-1.04±0.11	2.68±0.82^*^
FASN	Fatty acid synthase	-1.74±0.42^*^	-1.23±0.2	-1.12±0.07	-4.87±2.22^*^
INSIG1	Insulin induced gene 1	1.27±0.07	1.57±0.2^*^	1.02±0.04	-2.17±0.81^*^
LAMA1	Laminin subunit alpha 1	-1.06±0.15	-1.88±0.22^*^	1.17±0.16	-3.68±2.24^*^
MLXIPL	MLX interacting protein like	-1.24±0.21	-3.39±2.17^*^	1.01±0.01	-1.14±0.18
NCEH1	Neutral cholesterol ester hydrolase 1	-1.01±0.08	-2.44±1.29^*^	-1.06±0.03	-1.09±0.03
UGCG	UDP-glucose ceramide glucosyltransferase	-1.17±0.13	1.29±0.3	-1.08±0.05	3.69±1.48^*^
**Fatty acid oxidation/mitochondrial stress**					
ACSL1	Acyl-CoA synthetase long-chain family member 1	-1.01±0.05	-1.22±0.18	1.03±0.03	-4.23±2.01^*^
ACSL3	Acyl-CoA synthetase long-chain family member 3	-1.12±0.09	2.06±0.06^*^	1.39±0.06	1.63±0.06^*^
ACSL4	Acyl-CoA synthetase long-chain family member 4	-1.62±0.24	2.65±0.96^*^	1.08±0.12	6.25±3.38^*^
ACSL6	Acyl-CoA synthetase long-chain family member 6	1.66±0.21^*^	1.2±0.67	1.23±0.2	-1.95±0.44^*^
CLIC1	Chloride intracellular channel 1	1.1±0.06	1.92±0.59^*^	-1.06±0.04	3.11±0.98^*^
CPT1A	Carnitine palmitoyltransferase 1A	-1.06±0.03	-1.14±0.09	1±0.02	2.22±0.9^*^
**Lipid transport**					
CD36	CD36 Molecule	1±0.03	-2.03±1.77^*^	-1.21±0.07	-1.51±0.30^*^
FABP1	Fatty acid binding protein 1	4.66±2.72^*^	1.64±4.19	-1±0.03	-2.61±1.29
FABP7	Fatty acid binding protein 7	1.88±0.15^*^	2.34±0.98^*^	1.12±0.1	-1.12±0.06
SLC27A2	Solute carrier family 27 member 2	-1.15±0.07	-2.68±2.07^*^	-1.27±0.04	-3.56±2.52^*^
**LD growth/ER stress**					
APOE	Apolipoprotein E	1.17±0.12	3.6±0.6^*^	-1.01±0.12	-1.2±0.06
ERLIN1	ER lipid raft associated 1	1.02±0.02	2.25±0.78^*^	1.12±0.12	1.25±0.2
MGLL	Monoglyceride lipase	1.03±0.06	-3.05±4.32^*^	1.04±0.05	-1.53±0.28
PLIN2	Perilipin 2	1.65±0.26^*^	8.11±4.19^*^	1.41±0.3	2.26±1.21^*^
TIMP1	TIMP metallopeptidase inhibitor 1	1.04±0.06	18.9±13.11	-1.06±0.03	12.05±2.55^*^
**Lipid metabolism marker genes**					
FGF21	Fibroblast growth factor 21	1.64±0.44^*^	3.87±1.56^*^	1.03±0.05	-1.01±0.02
GLUD1	Glutamate dehydrogenase 1	1.08±0.09	-2.3±1^*^	1.02±0.02	-1.21±0.11
GPT	Glutamic-pyruvic transaminase	-1.14±0.11	-2.2±0.59^*^	1.17±0.13	-2.28±0.06^*^
KRT18	Keratin 18	-1.02±0.06	2.52±0.06^*^	1.23±0.08	2.68±0.06^*^
KRT8	Keratin 8	1.13±0.09	3.09±1.18^*^	1.1±0.06	2.54±0.81^*^
**Signalling events**					
ARG2	Arginase 2	1.06±0.05	2.18±0.39^*^	1.04±0.01	3.39±1.44^*^
CXCL14	C-X-C Motif chemokine ligand 14	-1.05±0.06	1.01±0.05	-1.28±0.17	2.01±0.71^*^
HIF1A	Hypoxia Inducible factor 1 alpha subunit	1.18±0.03	3.71±2.86^*^	1±0.01	5.21±2.13^*^
PEBP1	Phosphatidylethanolamine binding protein 1	-1.09±0.07	-2.54±1.43^*^	-1.04±0.03	-1.24±0.08
PRKAA1	Protein kinase AMP-activated catalytic subunit alpha 1	1.08±0.12	3.39±1.35^*^	-1.04±0.02	1.11±0.06
SERPINE2	Serpin family E member 2	-1.08±0.23	3.77±1.85^*^	-1.2±0.12	-1.09±0.36
STAT1	Signal transducer and activator of transcription 1	1.04±0.06	4.06±1.7^*^	-1.04±0.06	-1.18±0.23
STAT3	Signal transducer and activator of transcription 3	-1.15±0.03	2.37±0.69^*^	-1.07±0.04	2.25±0.99^*^
TLR4	Toll like receptor 4	1.04±0.02	2.11±0.74^*^	-1.08±0.14	-1-11±0.23
**Glucose metabolism**					
CES1	Carboxylesterase 1	1.14±0.06	-2.87±1.35^*^	-1.15±0.02	-3.86±2.09^*^
GK	Glycerol kinase	1.58±0.36^*^	3.06±0.67^*^	1.03±0.05	-1.64±0.15
PKLR	Pyruvate kinase, Liver and RBC	-1.78±0.38^*^	-8.22±5.31^*^	1.04±0.07	-1.42±0.48

Based on mechanistically linked and lipid droplet associated gene regulations, a total of 41 DEGs were identified [228, 229].

^*^ Statistically significant.

Diclofenac treatment caused similar but also organ specific genomic responses (Table 6) and a list of commonly regulated genes in liver and kidney was compiled. Given in Table 6 are 106 DEGs that could be categorized based on significantly enriched ontology terms. Among others the transferrin receptor, the early growth response 1, asparaginase synthase, cyclin dependent kinase inhibitor 1 and LY6E were regulated by 6-, 4-,5- and 4-fold in the liver, respectively, to indicate inflammation and cell death responses. With the exception of LY6E, however, the magnitude of regulation in kidney was approximatively half of that seen in liver. Conversely, the induction of gap junction protein ß2 and HIF1α was more pronounced in kidney as was the repression of the sulfotransferase 1B member 1; the latter supports excretion of drugs. Another highly regulated gene is the aldo-keto reductase family 1 member C3 (AKR1C3). This enzyme catalyzes metabolism of prostaglandin D2 to 9α, 11β-PGF2 and is a major prostaglandin released by mast cells to promote inflammation (Figure 9). This prostaglandin is therefore a sensitive marker of mast cell activation [63].

**Table 6:** Commonly regulated genes in liver and kidney after diclofenac treatment

Gene symbol	Gene Description	HD_liver	HD_kidney
		Fold change (average)±SD	
**Inflammatory response**			
APOC3	Apolipoprotein C3	-3.55±1.03	-2.05±1.95
CCL20	C-C motif chemokine ligand 20	7.28±3.13	2.54±1.13
CD163	CD163 molecule	2.3±0.89	2.27±0.2
FCGR1A (IgG)	Fc fragment of IgG receptor Ia	3.54±1.66	2.72±0.94
PTGS2	Prostaglandin-endoperoxide synthase 2	5.34±2.54	2.91±1.37
STAT3	Signal transducer and activator of transcription 3	2.37±0.69	2.25±0.99
TFRC (CD71)	Transferrin receptor	6.33±1.49	2.86±0.57
**Response to oxidative stress**			
AKR1C3	Aldo-keto reductase family 1 member C3	-14.39±9.74	-2.04±1.4
BTG3	BTG anti-proliferation factor 3	4.17±1.94	2.32±0.7
GJB2	Gap junction protein beta 2	3.35±1.06	7.97±5.58
HIF1A	Hypoxia inducible factor 1 alpha subunit	3.71±2.86	5.21±2.61
NQO1	NAD(P)H quinone dehydrogenase 1	-2.77±1.98	-2.37±0.48
SOD1	Superoxide dismutase 1	-2.46±0.97	-1.68±0.15
**Oxidation-reduction process**			
ACSS1	Acyl-CoA synthetase short-chain family member 1	2.5±1.01	2.12±0.88
AKR1E2	Aldo-keto reductase family 1 member E2	-2.7±3.82	-2.6±2.99
ALDH1B1	Aldehyde dehydrogenase 1 family member B1	-5.24±1.26	-2.09±1.19
CRYL1	Crystallin lambda 1	-3.02±0.81	-2.1±1.81
CYP4V2	Cytochrome P450 family 4 subfamily V member 2	-2.78±2.86	-2.43±1.55
DMGDH	Dimethylglycine dehydrogenase	-3.36±1.4	-2.32±0.68
FTH1	Ferritin heavy chain 1	-2.54±2.38	-2.01±0.59
G6PC	Glucose-6-phosphatase catalytic subunit	-2.2±1.08	-2.86±1.15
GPD1	Glycerol-3-phosphate dehydrogenase 1	-5.11±1.9	-1.92±0.38
HSD17B14	Hydroxysteroid 17-beta dehydrogenase 14	-2.61±2.87	-2±1.09
SLC27A2	Solute carrier family 27 member 2	-2.68±2.07	-3.56±2.52
TST	Thiosulfate sulfurtransferase	2.34±0.68	1.88±0.66
**Response to stress**			
ABHD2	Abhydrolase domain containing 2	2.05±0.46	1.92±0.51
UPP1	Uridine phosphorylase 1	7.84±1.94	6.19±0.49
VWF	Von Willebrand factor	4.22±1.71	1.91±0.31
**Xenobiotic metabolic process**			
CES1	Carboxylesterase 1	-2.87±1.35	-3.86±2.09
EPHX1	Epoxide hydrolase 1	-3.73±6.87	-1.91±0.88
GSTM3	Glutathione S-transferase mu 3	-9.55±1.75	2.34±0.25
GSTZ1	Glutathione S-transferase zeta 1	-2.51±1.86	-2.1±0.45
NR1I3 (CAR)	Nuclear receptor subfamily 1 group I member 3	-2.04±0.97	-1.94±0.74
SULT1A1	Sulfotransferase family 1A member 1	-2.69±1.83	-2.33±0.41
SULT1B1	Sulfotransferase family 1B member 1	-2.35±0.75	-6.53±1.94
**Cytokine-mediated signaling pathway**			
EGR1	Early growth response 1	6.6±3	2.36±1.13
IL13RA1	Interleukin 13 receptor subunit alpha 1	2.87±1.18	2.73±0.81
KRT18	Keratin 18	3.01±1.33	2.68±0.69
KRT8	Keratin 8	3.3±1.6	2.62±0.99
PLP2	Proteolipid protein 2	2.07±0.65	2.42±0.35
**Response to cytokine**			
ARG2	Arginase 2	2.18±0.48	3.39±1.44
HNMT	Histamine N-methyltransferase	-2.09±1.79	-1.98±0.8
**Complement activation**			
C5	Complement C5	2.71±1.03	2.2±0.53
CFI	Complement factor I	3.05±0.86	2.15±0.61
**Defense response**			
CD46	CD46 molecule	2.49±1.19	2.54±0.98
CXADR	Coxsackie virus and adenovirus receptor	2.24±0.59	2.3±0.25
HERC6	HECT and RLD domain containing E3 ubiquitin protein ligase family member 6	-2.36±0.67	2.06±0.24
LY6E	Lymphocyte antigen 6 complex, locus E	-4.34±2.11	-3.63±1.32
PRKCSH	Protein kinase C substrate 80K-H	2.59±0.99	3.52±1.12
**Cell cycle regulation**			
CENPM	Centromere protein M	-2.32±1.56	-2.91±1.38
CENPP	Centromere protein P	-2.49±2.03	-2.37±0.96
**Regulation of cell death and apoptosis**			
ASNS	Asparagine synthetase (glutamine-hydrolyzing)	4.63±1.48	2.27±0.48
CDKN1A	Cyclin dependent kinase inhibitor 1A	5.1±1.17	2.64±0.36
CLDN7	Claudin 7	2.26±0.36	2.75±0.94
CSTB	Cystatin B	4.1±1.89	3.43±0.8
CYLD	CYLD lysine 63 deubiquitinase	2.12±0.9	2.01±0.41
DNAJC3	DnaJ heat shock protein family (Hsp40) member C3	4.49±2.5	2.03±0.72
DNASE1	Deoxyribonuclease 1	-2.89±1.91	-3.71±2.34
GABRB3	Gamma-aminobutyric acid type A receptor beta3 subunit	-2.04±1.52	-1.96±0.52
LITAF	Lipopolysaccharide induced TNF factor	2.04±0.52	2.02±0.03
MCL1	BCL2 family apoptosis regulator	2.75±1.24	2.03±0.53
PDXK	Pyridoxal (pyridoxine, vitamin B6) kinase	2.08±0.77	2.43±0.96
PLK2	Polo like kinase 2	3.23±1.58	-2.17±1.34
RGN	Regucalcin	-3.24±2.28	-2.4±0.5
SDF2L1	Stromal cell derived factor 2 like 1	7.33±4.14	2.18±0.61
**Lipid metabolic process**			
ABCB4	ATP binding cassette subfamily B member 4	2.15±0.73	3.08±1.61
ACSM3	Acyl-CoA synthetase medium-chain family member 3	-2.72±1.44	-2.24±1.26
CRLS1	Cardiolipin synthase 1	-2.38±1.52	-2.38±1.06
ELOVL2	ELOVL fatty acid elongase 2	9.75±3.04	-2.03±1.09
IDI1	Isopentenyl-diphosphate delta isomerase 1	-2.08±0.49	-4.2±0.23
LRAT	Lecithin retinol acyltransferase	-2.7±3.2	-7.21±2.82
MOGAT3	Monoacylglycerol O-acyltransferase 3	-2.7±2.17	-2.69±1.79
NANS	N-acetylneuraminate synthase	-3.89±2.28	2.16±0.74
OXCT1	3-oxoacid CoA-transferase 1	-6.24±3.46	-1.98±0.59
PLIN2	Perilipin 2	8.11±4.19	2.26±1.21
TIMP1	TIMP metallopeptidase inhibitor 1	18.9±13.11	12.05±2.55
**Amino acid metabolic process**			
ACMSD	Aminocarboxymuconate semialdehyde decarboxylase	-2.1±1.45	-2.21±1.04
AGMAT	Agmatinase	-3.61±7.29	-3.45±3.4
GLYAT	Glycine-N-acyltransferase	-4.08±8.57	-2.25±0.86
GPT	Glutamic--pyruvic transaminase	-2.2±0.59	-2.28±0.06
PPA1	Pyrophosphatase (inorganic) 1	2.07±0.64	2.67±1.05
UPB1	Beta-ureidopropionase 1	-2.43±2.71	-2.65±0.73
**Protein metabolism**			
FUOM	Fucose mutarotase	-3.27±2.71	-2.12±0.36
TRAM1	Translocation associated membrane protein 1	5.72±3.12	1.92±0.65
WEE2	WEE1 homolog 2	-3.4±3.14	-2.17±0.51
**Fructose metabolism**			
FBP1	Fructose-bisphosphatase 1	-3.09±1.97	-1.9±1.08
GLYCTK	Glycerate kinase	-2.01±1.82	-1.88±0.69
**Anion transport**			
AGXT	Alanine-glyoxylate aminotransferase	-4.25±2.92	-1.94±0.25
SLC12A4	Solute carrier family 12 member 4	2.75±1.41	1.94±0.19
SLC16A4	Solute carrier family 16 member 4	-3.26±1.12	-2.21±1.08
SLC1A4	Solute carrier family 1 member 4	7.41±4.58	1.94±0.66
**Transporter activity**			
AQP11	Aquaporin 11	-2.45±3.67	-2.57±2.07
MVP	Major vault protein	2.86±1.06	2.65±0.41
NIPAL1	NIPA like domain containing 1	-2.39±0.09	-2.64±0.72
RBP5	Retinol binding protein 5	-2.85±7.37	-3.35±1.41
SFT2D2	SFT2 domain containing 2	2.31±0.54	1.88±0.68
**Transcriptional activation of mitochondrial biogenesis**			
MRPL22	Mitochondrial ribosomal protein L22	2.02±0.39	2.06±0.72
PPRC1	Peroxisome proliferator-activated receptor gamma, coactivator-related 1	2.73±1.12	2.06±0.54
**Extracellular matrix organization**			
CRISPLD2	Cysteine rich secretory protein LCCL domain containing 2	3.78±1.83	3.58±2.09
ECM2	Extracellular matrix protein 2	-4.39±2.21	-4.06±2 .0
**Regulation of epithelial cell differentiation and proliferation**			
PBLD	Phenazine biosynthesis like protein domain containing	-4.23±6.9	-2.92±1.34
TAGLN2	Transgelin 2	2.59±1	2.08±0.47
**Cell junction assembly**			
CD151	CD151 molecule (Raph blood group)	2.19±0.77	2.4±0.57
DSG1	Desmoglein 1	-3.91±2.69	-3.23±2.58
**Circadian rhythm**			
BHLHE41	Basic helix-loop-helix family member e41	-2.33±0.24	-2.84±1.37
DBP	D-box binding PAR bZIP transcription factor	-2.49±1.29	-3.12±1.67

The highly significant repression and near complete silencing of AKR1C3 implies an adaptive response to the mastocytosis induced by diclofenac treatment (Figure 5). Similarly, an induced expression of cystatin B suggests differentiation of monocytes into macrophages, and an independent study showed LPS treatment of human monocytes to induce cystatin B expression [64]. Indeed, the >30-fold induction of LBP in the liver of diclofenac treated animals highlights activation of the innate immune response though cystatin B may also function as an inhibitor of apoptosis as reported by others [65]. Testimony to drug induced inflammation is also the 19- and 12-fold induction of TIMP1, an inhibitor of metalloproteinases in liver and kidney. TIMP1 is highly induced by cytokines and was shown to inhibit cytokine dependent activation of NF-kappaB in pancreatic islets and beta-cells [66].

Furthermore, histopathology revealed almost complete depletion of hepatic glycogen in high dose treated animals to imply drug induced stress (Figure 5). We therefore investigated key member in glycogen synthesis and metabolism but only a small number of genes were significantly regulated (Supplementary Table 1); notwithstanding, the gene coding for the rate limiting step in glycolysis, i.e. PKLR was highly significantly repressed in expression to nearly 10% of control animals.

Upon diclofenac treatment various signaling pathways were regulated in liver and kidney (Supplementary Table 4) and included a 3-fold induction of glycerol kinase (GK). Note, GK is a key enzyme in triglyceride and glycerophospholipid synthesis and the near 4- and 3-fold repression of apolipoprotein C3 and the sterol carrier protein 2 define altered PPAR signaling. Alike, members of the PI3K-Akt pathway were regulated as shown by the 5-fold induction of the cyclin dependent kinase inhibitor 1A (p21) that blocks cell cycle progression, the >4-fold repression of insulin like growth factor 1 and the 4-fold induction of the Von Willebrand factor. Moreover, the 8- and 5-fold induction of the interleukin 1 receptor type 1 and 2 and the nearly 90% repression of the heat-shock cognate protein HSPA8 are testimony to an altered MAPK signaling pathway. Besides Fcγ receptor signaling was changed as demonstrated by the 4-fold induced expression of the Fc fragment of the IgG receptor Ia. Importantly, this receptor is strongly expressed on monocytes and macrophages and interacts with the Fc-fragment of IgG to adjust immune responses (Supplementary Table 4).

**Figure 16:**
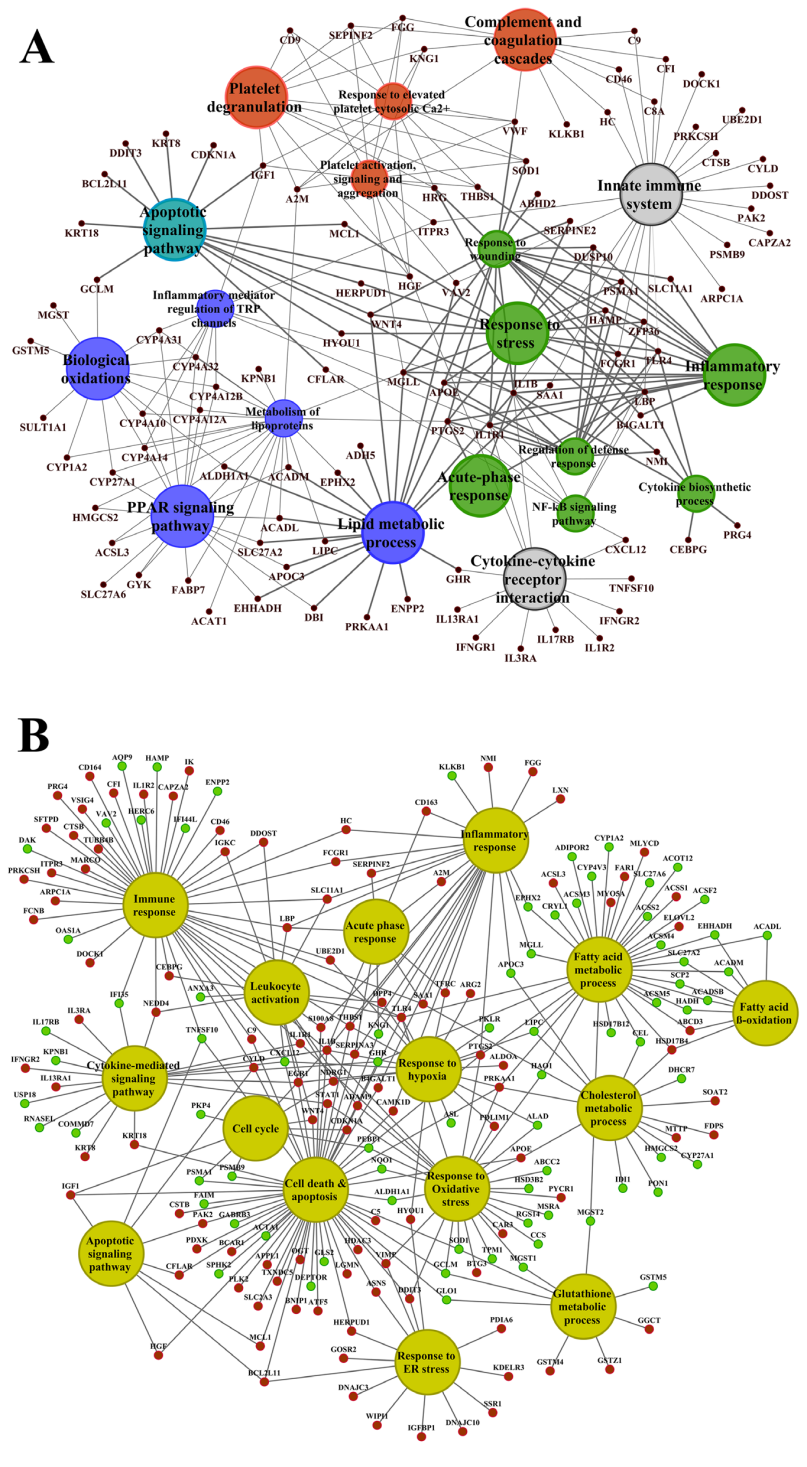
ClueGO gene ontology and pathway annotated hepatic networks in response to high dose diclofenac treatment A visualization of hepatic enriched pathway terms and biological processes computed with the ClueGo and the GeneXplain software of high dose treated animals. **(Panel A)** Network constructed by the plug-in ClueGO of the Cytoscape software. **(Panel B)** Network constructed manually using the GeneXplain platform. The enriched biological processes and pathways annotated with the GeneXplain platform and visualized with the Cytoscape software version 3.4. The red and green color nodes illustrate up- and down- regulated genes, respectively.

**Figure 17:**
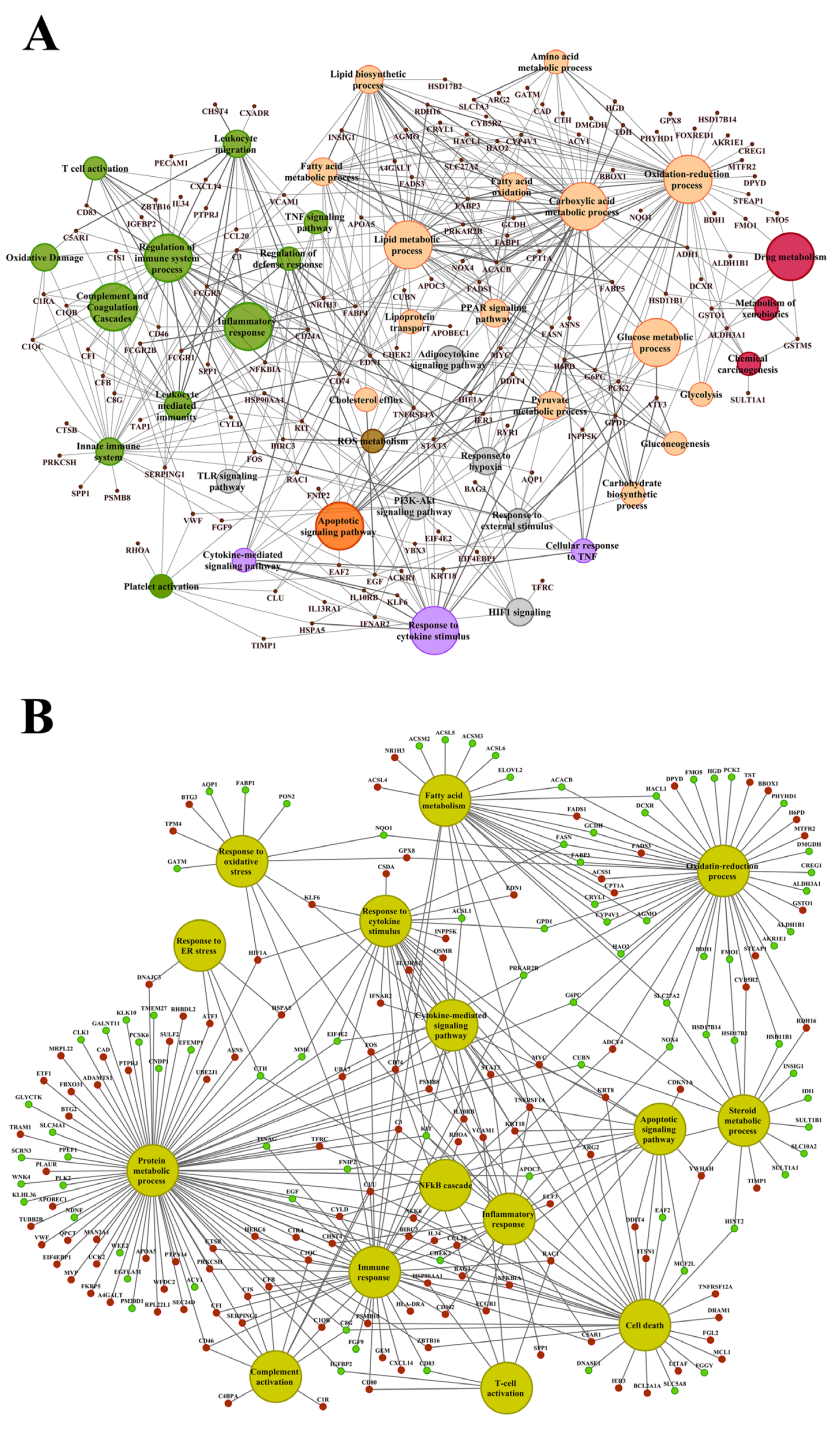
ClueGO gene ontology and pathway annotated networks of DEGs regulated in kidney in response to high dose diclofenac treatment A visualization of kidney enriched pathway terms and biological processes computed with the ClueGo and the GeneXplain software of high dose treated animals. **(Panel A)** Network constructed by the plug-in ClueGO of the Cytoscape software. **(Panel B)** Networks constructed manually using the GeneXplain platform. The enriched biological processes and pathways were annotated with the GeneXplain platform and visualized with the Cytoscape software version 3.4. The red and green color nodes illustrate up- and down- regulated genes, respectively.

A visualization of liver and kidney enriched pathway terms and biological processes computed with the ClueGo and the GeneXplain software is shown for high dose treated animals in Figures 16 and 17, and Supplementary Figures 5 and 6 inform on the low dose treatments.

### Drug metabolism and transporters

**Figure 18:**
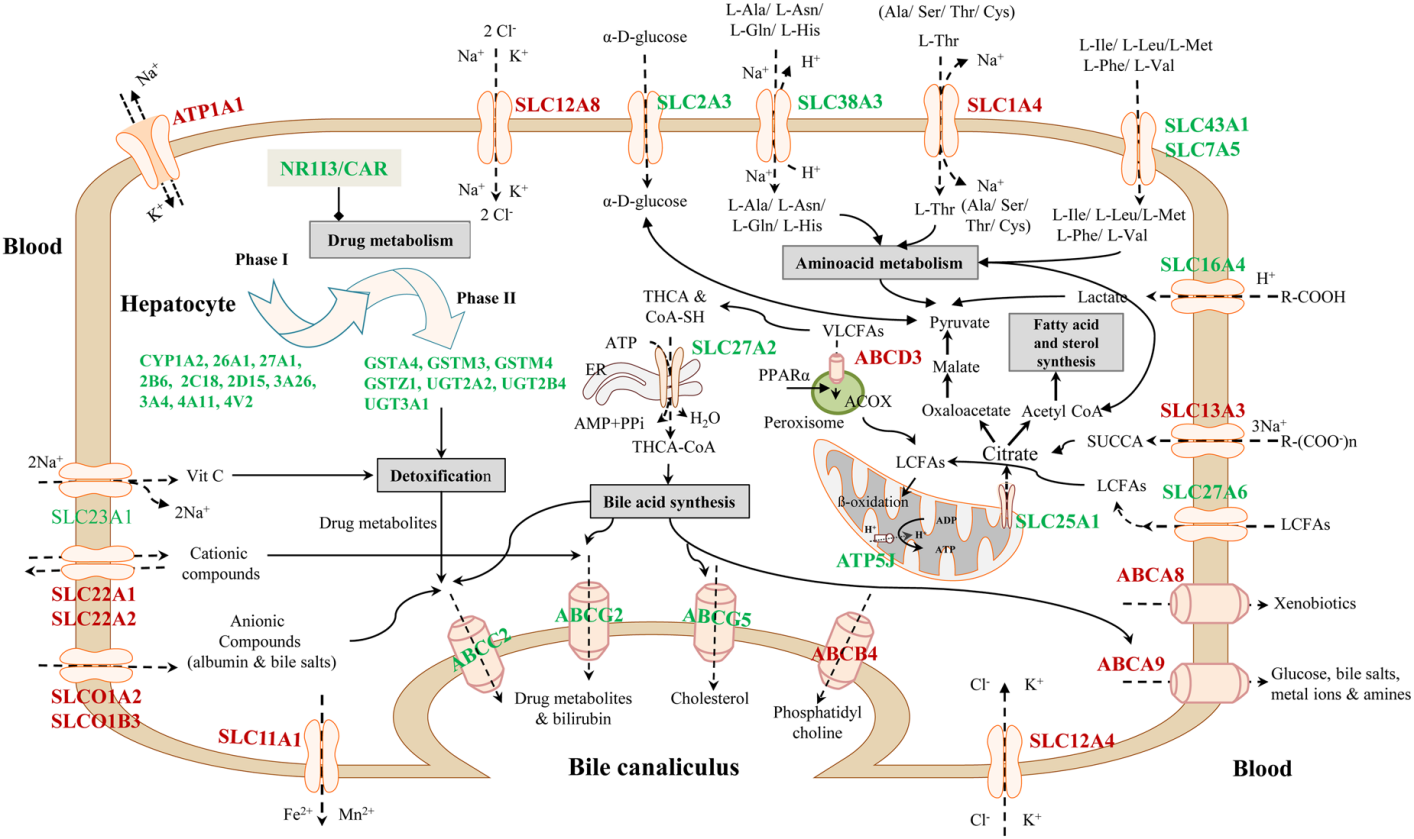
Schema of regulated drug metabolizing enzymes, solute carriers and transporters in the liver of diclofenac treated animals Genes shown in red are significantly up-regulated while those given in green are repressed in expression.

**Figure 19:**
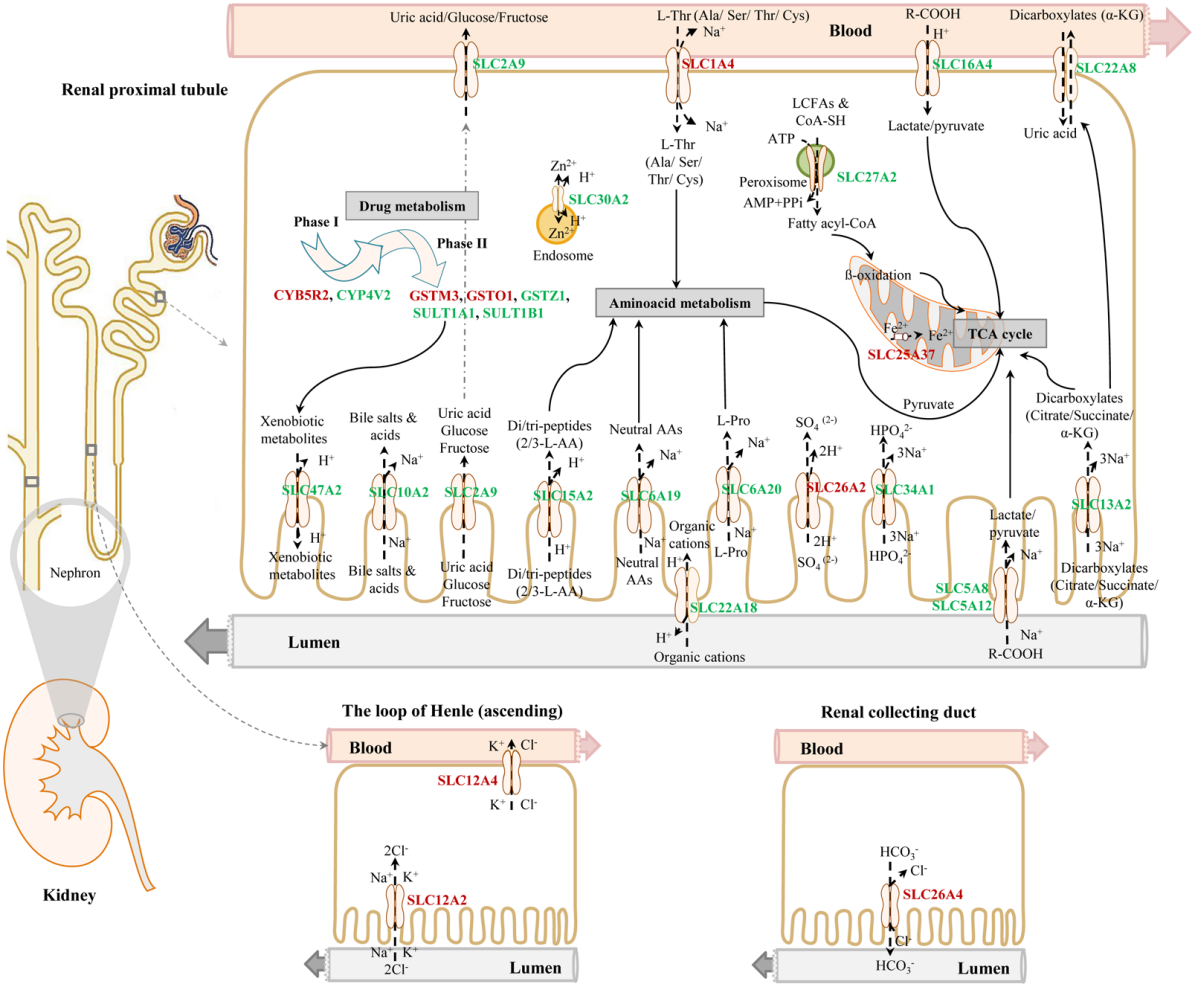
Schema of drug regulated metabolizing enzymes, solute carriers and transporters in the kidney of diclofenac treated animals Genes shown in red are significantly up-regulated while those given in green are repressed in expression.

Diclofenac is extensively metabolized; we therefore considered regulation of CYP monooxygenases and transporters involved in the metabolism and excretion of diclofenac and its metabolites. Predominant repression of hepatic CYP2A7 and CYP2A13 in the low dose and up to 90% repressed transcripts coding for CYP1A2, CYP27A1, CYP2C18, CYP2D15, CYP3A26, CYP3A4, CYP4A11, CYP4V2 in the high dose treatment was observed (Supplementary Table 5). Notwithstanding, CYP26A1 and CYP2B6 were commonly and dose dependently repressed among the two treatment groups. With kidney and at the low-dose CYB5B was repressed in expression. Conversely, a 2-fold increase in CYB5R2 and a similar 2-fold repression in CYP4V2 were determined for high dose treated animals. Among the Phase II drug metabolizing enzymes the glutathione and glucuronyl transferases GSTA4, GSTM3, GSTM4 and UGT2B4 were significantly repressed (up to 90%) in the liver of diclofenac treated animals. Similarly, transcripts of the sulfotransferase SULT1A1 and SULT1B1 were significantly repressed in kidney, and except of SLC1A4 and SLC13A3 which were induced by 7- and 3-fold, respectively, most of the transporters and mitochondrial solute carriers were repressed in expression (Supplementary Table 5). The reduced expression of the canalicular transporters ABCC2, ABCG2 and of the cholesterol transporter ABCG5 leads to impaired bile acid, bilirubin and cholesterol excretion and in the long term to intrahepatic cholestasis as was observed in DILI patients treated with diclofenac. Conversely, ABCA8 and ABCA9 were induced in expression (approx. 3-fold) to support efflux of drug metabolites and bile salts across the basolateral membrane into the systemic circulation. A summary of regulated drug metabolism, transporters and solute carriers in liver and kidney is shown in Figures 18 and 19.

### Markers of innate immune competent cells

The macrophage marker genes ARG2, CCL20, CD163, and CXCL8/IL-8, IgG FCγ receptor I, macrophage receptor with collagenous structure (MARCO), COX2 and C-reactive protein were up to 7- and 3-fold induced in liver and kidney, respectively, after repeated treatment for 28 days. Alike the T-, B- and Th-cell marker genes C3, DPP4, EGR1, IFNGR2, IL1B, STAT1, STAT3 and TLR4 were significantly increased up to 7-fold, and a similar up to 10-fold induction of the granulocyte markers CD9, CD63, CD164, ICAM-1, LRG1 and S100A8 was determined in the liver of high dose treated animals (Table 7 and Supplementary Table 6). Conversely, IL17RB and LY6E were repressed in expression.

**Table 7:** Gene expression markers of immune cells significantly regulated in diclofenac treated animals

Gene symbol	Gene Description	Liver		Kidney	
		LD	HD	LD	HD
		Fold change (average)± SD			
**Macrophage activation marker genes**					
**M1 polarization**					
C5AR1	Complement C5a Receptor 1	-1.06±0.04	1.65±0.41	-1.02±0.05	2.2±0.44^*^
CCL2	C-C motif chemokine ligand 2	1.01±0.1	2.34±1.74	-1.01±0.14	2.5±1.08^*^
CCL20	C-C Motif Chemokine Ligand 20	-1.02±0.11	7.28±3.13^*^	-1.3±0.13	2.54±0.1^*^
CD163^#^	CD163 molecule	-1.01±0.03	2.3±0.89^*^	-1.03±0.02	1.26±0.24
CD80	CD 80 molecule	1.18±0.14	1.53±0.24	1.2±0.05	2.02±0.66^*^
CRP	C-reactive protein	-2.19±1.5^*^	7.61±3.63^*^	1.03±0.04	-1.13±0.23
CXCL10^§^	C-X-C motif Chemokine Ligand 10	-1.5±0.42^*^	-1.17±0.48	1.18±0.23	5.45±3.02^*^
FCGR1A (IgG)	Fc Fragment Of IgG Receptor Ia	1.1±0.06	3.54±1.66^*^	1.1±0.08	2.72±0.77^*^
HIF1A	Hypoxia Inducible Factor 1 Alpha Subunit	1.18±0.03	3.71±2.86^*^	1±0.01	5.21±2.61^*^
HLA-DRB1 (MCH2)^ǂ^	Major Histocompatibility Complex, Class II, DR Beta 1	1.11±0.13	1.29±0.16	1.18±0.22	3.57±0.45^*^
IDO1	Indoleamine 2,3-Dioxygenase 1	1.17±0.23	-1.18± 0.19	-1.08± 0.08	-1.86±0.5^*^
IFNAR1	Interferon Alpha And Beta Receptor Subunit 1	1.02±0.1	1.35±0.27	1.18±0.09	1.57±0.21^*^
IFNAR2	Interferon Alpha And Beta Receptor Subunit 2	-1.09±0.07	-1.07±0.08	1.01±0.01	2.37±0.06^*^
IFNGR2	Interferon Gamma Receptor 2	1.31±0.11	2.23±0.93^*^	1.19±0.07	1.28±0.1
IL1B^ɸ^	Interleukin 1 beta	1.82±0.32^*^	2.02±0.16^*^	1.07±0.19	1.07±0.22
IL1R1	Interleukin 1 Receptor Type 1	1.01±0.03	8.23±1.92^*^	1.04±0.08	2.6±2.21^*^
IL8/CXCL8	Interleukin 8	1.18±0.15	2.85±1.65^*^	-1.3±1.07	1.2±0.15
INHBA	Inhibin Beta A Subunit (Activin A)	1.07±0.08	1.37±0.43	1.14±0.1	1.74±0.37^*^
IRF3	Interferon Regulatory Factor 3	1.09±0.07	1.06±0.09	1.04±0.03	1.82±0.26^*^
MYD88	Myeloid Differentiation Primary Response 88	-1.19±0.13	-1.17±0.14	1.14±0.16	1.76±0.34^*^
PTGS2/COX2	Prostaglandin-Endoperoxide Synthase 2	-1.07±0.03	5.34±2.54^*^	1.17±0.08	2.91±1.37^*^
STAT1^ɸ^	Signal Transducer And Activator of Transcription 1	1.04±0.06	4.06±1.7^*^	-1.04±0.06	-1.18±0.23
TLR2	Toll like receptor 2	1.14±0.17	1.6±0.24^*^	1.11±0.08	1.48±0.38
TLR4^¥^	Toll Like Receptor 4	1.04±0.02	2.11±0.74^*^	-1.08±0.14	-1-11±0.23
**M2a polarization**					
ARG2	Arginase 2	1.06±0.05	2.18±0.39^*^	1.04±0.01	3.39±1.44^*^
CD14	CD14 molecule	1.11±0.2	2.11±0.65^*^	1.14±0.12	3.89±1.96^*^
IL10RB	Interleukin 10 receptor subunit beta	1.07±0.05	1.74±0.71^*^	-1.1±0.07	3.22±1.24^*^
IL13RA1	Interleukin 13 Receptor Subunit Alpha 1	1.23±0.13	2.87±0.98^*^	1.1±0.05	2.73±0.81^*^
IL1R2	Interleukin 1 Receptor Type 2	1.02±0.06	4.96±2.1^*^	-1.5±0.07	1.29±0.27
MARCO	Macrophage Receptor With Collagenous Structure	1.36±0.22	2.61±0.77^*^	1.14±0.05	1.36±0.04
MPO	Myeloperoxidase	1.38±0.13	2.35±1.19^*^	-1.06±0.05	1.02±0.09
STAT3^¤^	Signal Transducer and Activator of Transcription 1	-1.15±0.03	2.37±0.69^*^	-1.07±0.04	2.25±0.99^*^
TGM2	Transglutaminase 2	1.28±0.22	4.48±3.07^*^	-1.11±0.1	1.08±0.06
**M2d polarization**					
ADK	Adenosine Kinase	-1.2±0.11	-1.6±0.17^*^	1.26±0.05	-1.28±0.03
CXCL16	C-X-C motif Chemokine Ligand 16	1.01±0.07	1.3±0.29	1.25±0.35	12.25±2.83^*^
VEGFA	Vascular Endothelial Growth Factor A	1.14±0.12	1.77±0.46^*^	-1.13±0.11	-1.15±0.19
**Sensors and effectors of M2 macrophage activation**					
C1QA	Complement C1q A Chain	1.03±0.02	-1.03±0.22	-1.01±0.08	1.80±0.67^*^
C1QB	Complement C1q B Chain	1.43±0.37	1.58±0.38^*^	1.15±0.09	1.95±0.69^*^
C1QC	Complement C1q C Chain	1.03±0.03	1.32±0.16	1.22±0.18	2.77±0.96^*^
IGF1	Insulin Like Growth Factor 1	1.09±0.16	-4.25±1.7^*^	-1.08±0.05	-1.19±0.29
NPY1R	Neuropeptide Y Receptor Y1	1.03±0.06	1.14±0.11	-1.11±0.09	-2.55±0.97^*^
**T-, B- and Th-cell markers**					
C3	Complement factor 3	1.32±0.01	1.96±0.03^*^	1.18±0.13	6.12±2.01^*^
DPP4	Dipeptidyl Peptidase 4	1.91±0.7	3±1.26^*^	-1.07±0.02	-1.14±0.13
EGR1	Early Growth Response 1	2.56±0.59	6.6±2.45^*^	-1.21±0.15	2.36±1.13^*^
IFNGR2	Interferon Gamma Receptor 2	1.1±0.09	2.23±0.76^*^	1.03±0.05	1.12±0.08
IL17RB	Interleukin 17 Receptor B	-1.15±0.04	-2.41±1.09^*^	1.04±0.04	-1.29±0.46
SLC11A1	Solute Carrier Family 11 Member 1	1.07±0.07	2.75±0.9^*^	-1.05±0.09	2.71±0.07^*^
**Granulocyte markers**					
CD116/CSF2RA	Colony Stimulating Factor 2 Receptor Alpha Subunit	1.12±0.02	1.88±0.17^*^	1.05±0.05	1.76±0.32^*^
CD164	CD164 Molecule	1.19±0.17	2.92±0.95^*^	1.33±0.03	1.85±0.48^*^
CD62/SELP	Selectin P	1.38±0.08	1.96±0.38^*^	1.13±0.06	1.44±0.11
CD63	CD63 Molecule	1.34±0.2	2.66±0.88^*^	1.04±0.01	1.63±0.07
CD9	CD9 Molecule	1.78±0.48	2.81±1.18^*^	-1.09±0.16	-1.52±0.31
CMA1	Chymase 1	-1.02±0.04	-2.29±0.94^*^	1.11±0.05	-1.16±0.1
ICAM1	Intercellular Adhesion Molecule 1	-1±0.03	2.38±1.02^*^	1±0.04	1.62±0.42
LRG1	Leucine Rich Alpha-2-Glycoprotein 1	-1.12±0.06	2.56±0.76^*^	-1.02±0.02	20.25±13.17
LY6E	Lymphocyte Antigen 6 Complex, Locus E	-1.18±0.28	-4.34±2.11^*^	1.04±0.26	-3.63±1.32^*^
NCF1	Neutrophil Cytosolic Factor 1	1.03±0.01	1.89±0.69^*^	1.37±0.13	1.33±0.32
S100A8	S100 Calcium Binding Protein A8	1.57±0.08	9.77±2.02^*^	1.08±0.13	5.25±3.35

The marker genes signify polarized macrophages, T-, B-, Th-cells and granulocytes. Some marker genes were commonly expressed among different immune cells and are highlighted by the following symbols.

^#^ M1, M2a, M2c and M2-tissue resident.

^ǂ^ M1, M2a and M2b.

^¥^ M1, M2 sensors and T-, B- and Th-cell marker.

^§^ M1 and M2d.

^ɸ^M1 and T-, B- and Th-cell marker.

^¤^ M2a and T-, B- and Th-cell marker.

^*^ Statistically significant.

In kidney C3 expression was induced by 6-fold and an up to 3-fold induction for SLC11A1, EGR1 and STAT3 was observed. Although statistically insignificant LRG1 was highly induced by 20-fold; nonetheless the acute phase protein and granulocyte marker S100A8 was statistically significantly induced by 5-fold.

**Figure 20:**
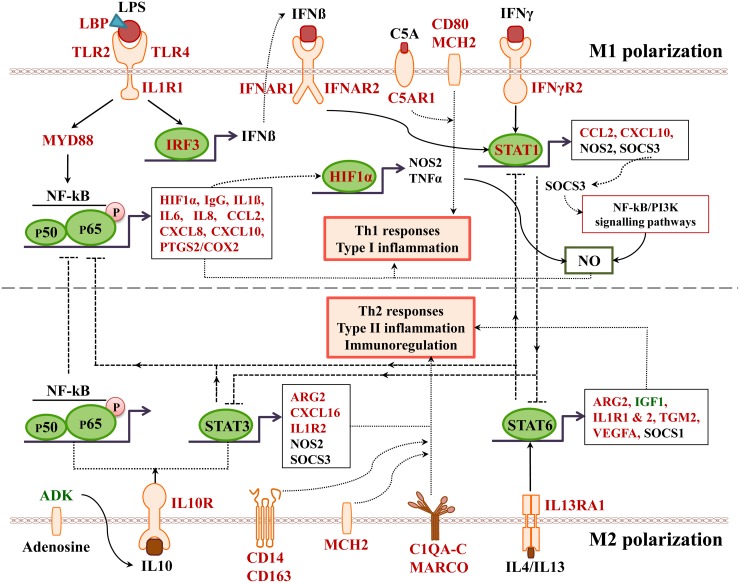
Schema of regulated marker genes associated with M1 and M2 polarized macrophages in the liver of diclofenac treated animals Genes shown in red are significantly up-regulated while those given in green are repressed in expression. Expressed but unchanged genes in canonical pathways are shown in black.

Shown in Table 7 are marker genes of immune cells to evidence a heterogeneous population of polarized macrophages and cytotoxic lymphocytes in response to diclofenac treatment, and Figure 20 summarizes the molecular signals of M1 and M2 polarized macrophages (see also Supplementary Table 6 for insignificantly regulated marker genes).

### STRING PPI networks

**Figure 21:**
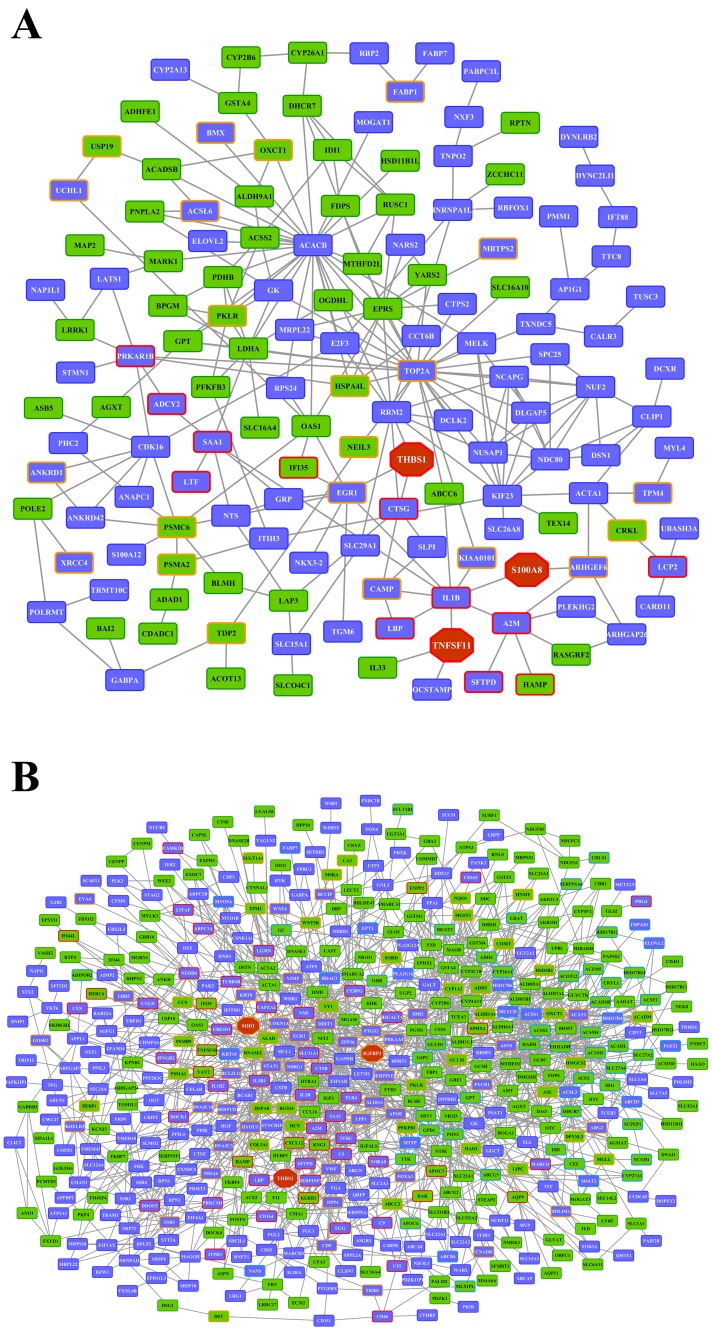
STRING protein-protein interaction networks in the liver of diclofenac treated animals **(Panel A)** Low dose treatment. A total of 287 DEGs were used to construct a PPI network of which 148 interacted among 258 PPIs. The purple and green color highlights up- and down-regulated DEGs. **(Panel B)** High dose treatment. A total of 663 DEGs were used to construct a PPI network of which 519 DEGs interacted among 1749 PPIs. The purple and green color highlights the up- and down-regulated DEGs. The red hexagon denotes master regulator molecules of the network. Immune and inflammation response genes were marked by red colored boxes, whereas stress response genes and lipid metabolic genes are highlighted in orange and blue colored boxes, respectively. The protein interaction networks were constructed using STRING version 10.0. Zoom in for better readability of individual genes.

**Figure 22:**
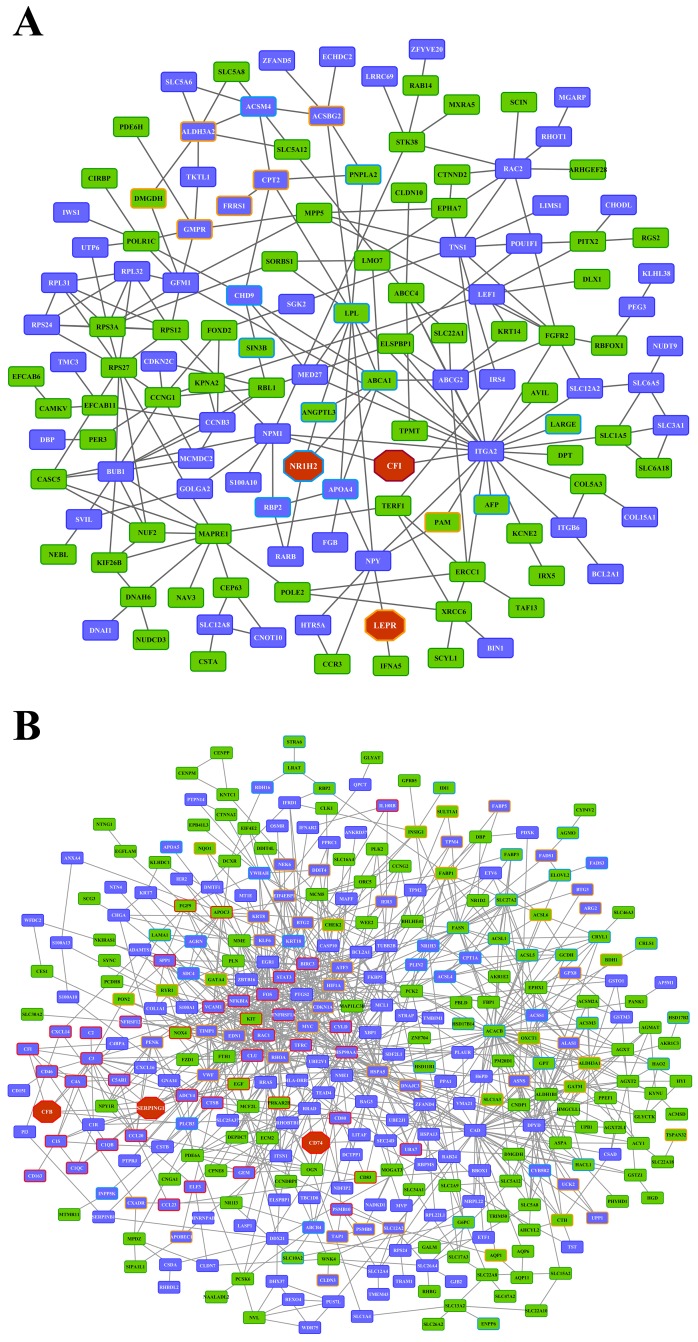
STRING protein-protein interaction networks in kidney of diclofenac treated animals **(Panel A)** Low dose treatment. Out of 249 DEGs a network that consists of 135 DEGs was constructed and interacted among 227 PPIs. **(Panel B)** High dose treatment. Out of 488 DEGs a network that consists of 338 DEGs was constructed and interacted among 1031 PPIs. The purple and green color rectangles highlight the up- and down-regulated DEGs and red hexagon indicates master regulatory molecules of the network. Immune and inflammation response genes were marked by red colored boxes, whereas stress response genes and lipid metabolic genes are highlighted in orange and blue colored boxes, respectively. Zoom in for better readability of individual genes.

Interaction networks for liver and kidney regulated genes were constructed by mapping the data to the STRING database. Among hepatic DEGs 52% (148 out of 276 genes) and 78% (519 out of 663 genes) engaged in 258 and 1749 protein-protein interactions (PPIs) after low and high dose treatments (Figure 21A and 21B). Likewise, 54% (135 out of 246 genes) in the low and 69% (338 out of 488 genes) in the high dose were regulated in kidney and engaged in 227 and 1031 PPIs, respectively (Figure 22A and 22B). The PPI networks could be consolidated by considering biological processes to reveal networks of immune, inflammation, acute phase and stress responses.

### Master regulator genes and their associated networks

In an effort to determine regulatory gene networks the GeneWays platform was queried; in the case of liver specific gene regulations thrombospondin 1 (THBS1), TNF (ligand) superfamily, member 11 (TNFSF11), S100 calcium binding protein A8 (S100A8) and IL-17A were identified as master regulators. After low dose diclofenac treatment the constructed networks consisted of 58, 60, 59 and 45 significantly regulated DEGs (about 21% of DEGs). Alike, the high dose diclofenac treatments linked 22% of hepatic DEGs in networks of THBS1 (160 DEGs), superoxide dismutase 1 (SOD1) (144 DEGs) and insulin-like growth factor binding protein 1 (150 DEGs). The same methodology was applied to kidney specific gene regulations. This defined the leptin receptor (LEPR), nuclear receptor subfamily 1, group H, member 2 (NR1H2) and complement component factor I (CFI) as master regulators. Here, 17% of DEGs of the low dose treatment were connected to the constructed networks. Alike after high dose diclofenac treatment 18% of DEGs were allied in networks linked to serine peptidase inhibitor, clade G, member 1 (SERPING1), CD74 antigen (CD74), and complement factor B (CFB). It is of considerable importance that except for IL-17A the master regulators themselves were significantly regulated in liver and kidney of low and high dose treated animals (Table 8).

**Table 8:** Master regulatory genes in liver and kidney after low and high dose diclofenac treatments

Master regulatory genes	No of genes		Score	FDR	Z-score	Fold change (average)±SD
	Total no. of genes in the network	Statistically significant DEGs				
**Low dose_liver**						
Thrombospondin 1 (THBS1)	120	58	0.50532	0.003	2.38305	1.74±0.48
Tumor necrosis factor (ligand) superfamily, member 11 (TNFSF11)	123	60	0.52204	0.016	1.8603	1.54± 0.18
S100 calcium binding protein A8 (S100A8)	120	59	0.49161	0.032	1.66594	1.57±0.1
Interleukin 17A (IL17A)	83	45	0.34041	0.018	2.80822	1.14±0.20
**High dose_liver**						
Thrombospondin 1 (THBS1)	287	160	0.43202	0.021	2.29552	2.14±0.55
Superoxide dismutase 1, soluble (SOD1)	245	144	0.32782	0.043	2.01236	-2.46±0.97
Insulin-like growth factor binding protein 1 (IGFBP1)	261	150	0.34794	0.045	2.13555	5.97±3.56
**Low dose_kidney**						
Leptin receptor (LEPR)	114	50	0.39552	0.038	1.7895	-1.64±0.2
Nuclear receptor subfamily 1, group H, member 2 (NR1H2)	95	45	0.36826	0.05	1.59419	-1.51±0.31
Complement factor I (CFI)	59	30	0.20321	0.03	2.01247	-1.62±0.22
**High dose_kidney**						
CD74 molecule (CD74)	138	85	0.20624	0.025	2.02739	5.34±0.97
Serpin peptidase inhibitor, clade G (C1 inhibitor), member 1 (SERPING1)	180	103	0.34598	0.031	2.18179	2.03±0.59
Complement factor B (CFB)	117	73	0.20292	0.018	2.7436	5.56±2.08

The key master molecules were identified and their regulatory networks were constructed using the GeneXplain software version 3.0. Given is a summary of master regulatory genes and its associated networks with the number of total interacting genes and DEGs, network score, Z-score and average fold change. The filtering criteria Z- Score>1 and Score>0.2 was set to select statistically significant master regulators.

We next considered cross-talk amongst individual gene networks and therefore constructed an integrated master regulatory network. The liver networks consisted of 22% and 24% of DEGs in low and high dose diclofenac treatment regimens. Likewise, 21% of DEGs were regulated in common in kidney specific networks (Supplementary Figures 7 and 8). Note, the complement factor CFI was also regulated in fused gene networks of the liver.

### Co-occupancy of TFBS in promoters of regulated genes

To gain further insight into regulatory gene networks, the enriched transcription factor binding sites at gene specific promoters coding for inflammation, immune, stress, hypoxia, acute-phase response, cytotoxicity (cell death and apoptosis) and oxidation-reduction were investigated. The computational analysis utilised positional weight matrices available within the MatInspector database of the Genomatix software suite (Supplementary Tables 7-10). The co-occupancy of different TFBS at gene specific promoters was also analysed with the FrameWorker tool that encompasses the vertebrate matrix library 9.2 of the MatInspector database. Among enriched TFBS we found two significant modules with two elements in liver specific gene regulations after low dose diclofenac treatment and these consisted of ETS1 and P53 (Supplementary Figure 9A1) and the autoimmune regulator AIRE and E2F-myc activator/cell cycle regulator (E2F) family transcription factors (Supplementary Figure 9A2). At the high dose the Krüppel-like transcription factor KLF6 and E2F1 were significantly enriched (Supplementary Figure 9B). Composite modules were also constructed for kidney associated DEGs and consisted of MYOD and E2F1 for low (Supplementary Figure 10A) and CREB and GATA for high dose treatments (Supplementary Figure 10B).

### Highly regulated genes

A total of 28 and 23 genes were >5-fold regulated in liver and kidney, respectively (Supplementary Table 3), and notable examples of hepatic inflammatory and immune responses are the induced expression of alpha-2 macroglobulin, CRP, transferrin receptor as well as hepcidin antimicrobial peptide, proteoglycan 4, uridine phosphorylase 1, early growth response 1 and ficolin 1; the latter is part of the lectin pathway of the complement system. Alike the cyclin dependent kinase inhibitor 1 A was significantly induced. Further examples include fatty acid elongase 2 and perilipin 2 which were >9 fold increased in expression. Their regulation is associated with oxidative stress and hepatic steatosis by increasing intracellular triacylglycerol synthesis and lipid droplet accumulation [67]. Furthermore, carbonic anhydrase III (CA III) was significantly down-regulated in liver; repression of this protein was reported to increase oxidative stress in hepatocytes after administration of ethanol and carbon tetrachloride [68].

With kidney highly regulated genes included increased expression of secreted phosphoprotein 1, CXCL10 and HLA-DRA that latter being induced by 10-fold. As seen with the liver uridine phosphorylase 1 was markedly increased in expression as was the chaperone clusterin that is regulated in conditions of oxidative stress and inflammation to influence programmed cell death.

### Cross-validation of gene expression data by RT-qPCR

**Figure 23:**
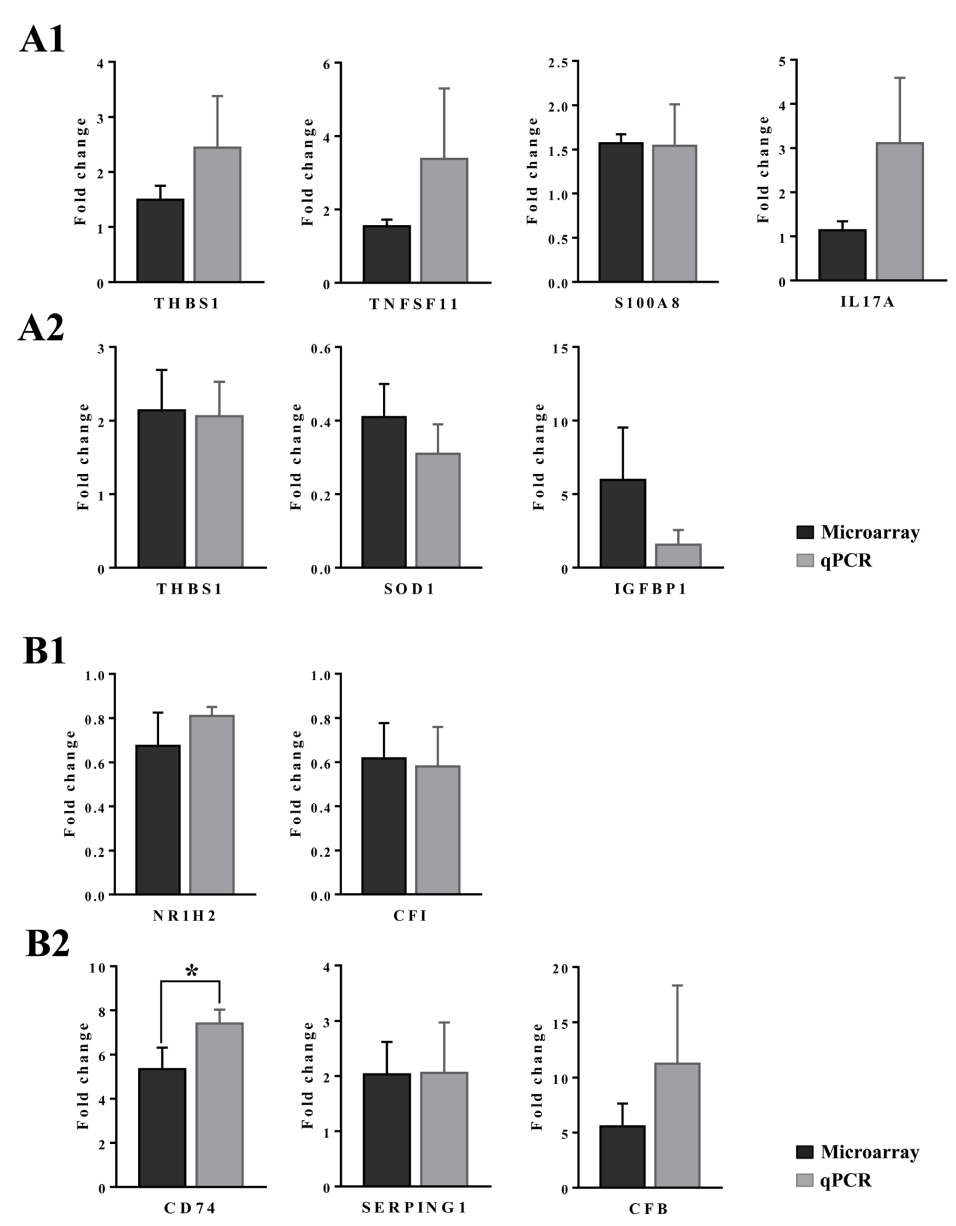
Experimental validation of master regulators by quantitative real-time PCR **(Panel A1 and A2)** depict the expression of hepatic master regulator genes after low and high dose diclofenac treatment, respectively. **(Panel B1 and B2)** illustrate expression of master regulatory genes in kidney after low and high dose diclofenac treatment, respectively. The *y*-axis indicates the average fold change in expression (diclofenac-treated *vs*. controls). Data are means ± SD (*n* = 3).

**Figure 24:**
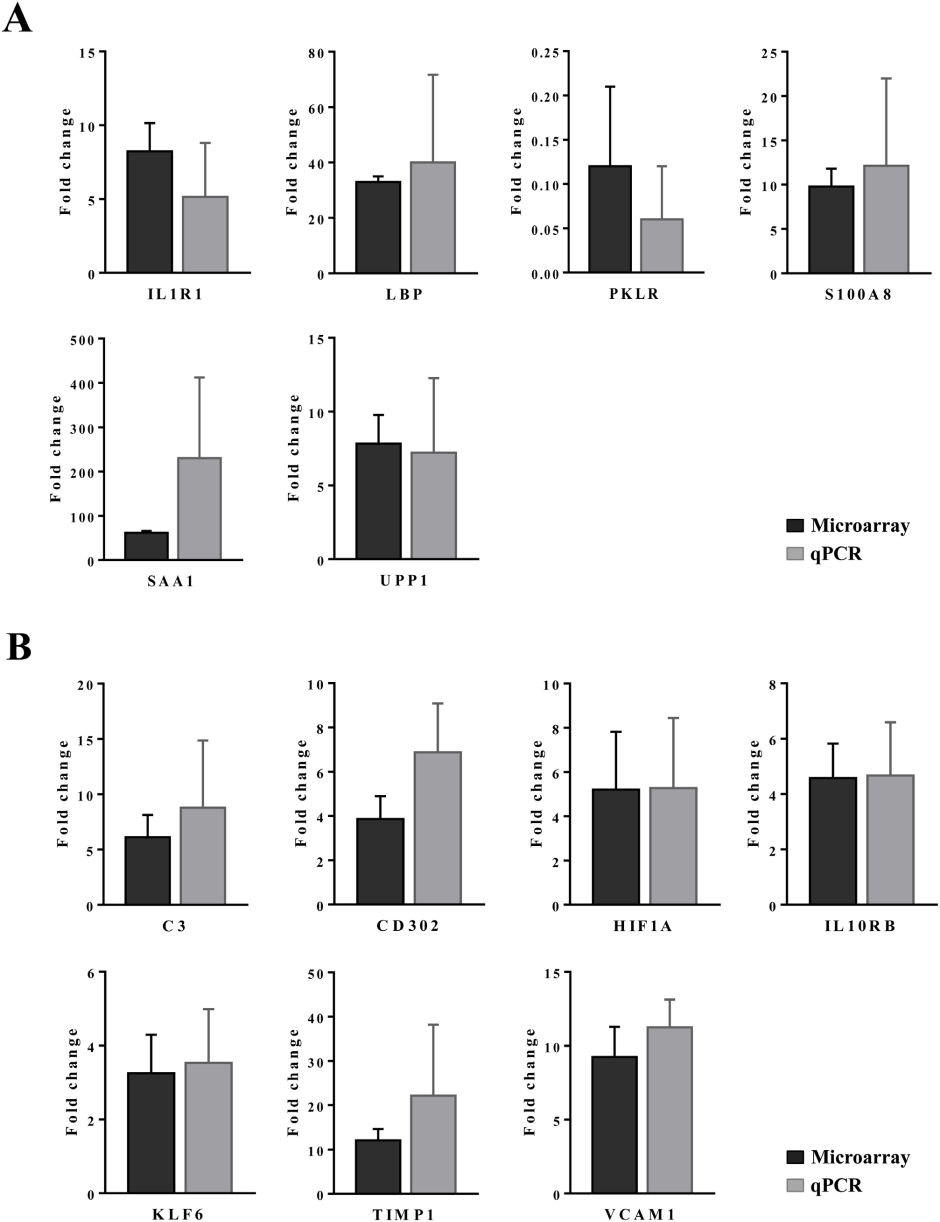
Experimental validation of highly regulated genes in liver and kidney in response to diclofenac treatment **(Panel A)** Highly regulated genes in liver. **(Panel B)** Highly regulated genes in kidney. The *y*-axis indicates the average fold change in expression (diclofenac-treated *vs*. controls). Data are means ± SD (*n* = 3).

To confirm the microarray data with another method RT-qPCR assays were performed. We selected genes coding for master regulators and highly regulated ones. As shown in Figures 23A and 23B similar results were obtained when fold changes among the two platforms were compared. Notwithstanding the RT-qPCR assays revealed the master regulators THBS1, TNFSF11, IL17A to be consistently higher in low dose treated animals though SOD1 and IGFBP1 was lower in expression in high dose treated animals (Figure 23A). Furthermore, RT-qPCR suggested higher fold-changes for CD74 and complement factor B (Figure 23B). We next considered highly regulated genes and once again observed agreement among the two platforms. Notwithstanding IL1R1 and PKLR were less but SAA1 was increased in hepatic expression. Similar the RT-qPCR assays revealed increased expression of C3, CD302, TIMP1 and VCAM-1 in kidney (Figure 24A and 24B).

## DISCUSSION

Idiosyncratic DILI is an unpredictable event [69] and risk factors associated with it were summarized in the seminal review of Chalasani and Björnsson [70].

Diclofenac is one of the most commonly used NSAID around the world, however burdened with significant risks for ADRs. Recently, the Pharmacovigilance Risk Assessment Committee (PRAC) of the European Medical Agency endorsed new measures to minimize risk for adverse drug reactions with diclofenac medications. This NSAID though effective in the management of pain and inflammation is associated with an increased risk for arterial thromboembolic events and liver and kidney injury. The mechanism of diclofenac mediated liver and kidney injury remains to be clarified. Frequently elevated serum transaminases are observed in patients on chronic drug use and histopathology is typically associated with necrosis (zone 3) and inflammation (portal/peri-portal). Chronic liver injury induced by diclofenac may involve interface hepatitis and even fibrosis as well as mixed hepatocellular cholestatic hepatitis. Alike, the spectrum of syndromes associated with diclofenac induced kidney injury includes acute kidney injury/tubulonecrosis, interstitial nephritis, membranous nephropathy, hyponatremia, hypokalemia, hypertension, papillary necrosis and analgesic nephropathy.

In an effort to define mechanism(s) of diclofenac induced allergic liver injury, whole genome expression profiling was performed. This revealed significant changes in the regulation of immune, stress, inflammation, apoptotic and ADME-coding genes in response to diclofenac treatment. Several lines of evidence suggest a critical role of diclofenac reactive metabolites in eliciting immune mediated responses. We observed marked induction of myeloperoxidase expression in Kupffer cells and cellular depletion of SOD1 as a result of oxidative stress. Importantly MPO catalyzes diclofenac reactive metabolism therefore linking innate immune cells of the liver to the production of reactive metabolites. We further assessed genes coding for drug metabolism and transport and observed highly significant repression of CYP monooxygenases in the liver but not in kidney of treated animals. Alike, various glutathione-S- and UDP-glucuronyl transferases were repressed. The significant repression of hepatic CYP monooxygenases and phase II enzymes may be caused by the immune response. Testimony to diclofenac induced sterile inflammation is the highly significant regulation of acute phase proteins with a >60-fold induction of serum amyloid A (SAA), i.e. the foremost acute phase protein, as well as α-2 macroglobulin, fibrinogen, various complement factors in addition to a number of cytokines. A major function of SAA is inhibition of myeloperoxidase (MPO) and activation of cytotoxic lymphocyte. The significant induction of SAA can be considered as an adaptive response to protect against the harmful effects of MPO and exaggerated reactive metabolite production. Similarly, the regulation of major solute carriers in liver and kidney is an important finding and differed among the two organs (Supplementary Table 5). The hepatic expression of SOD1 was decreased after diclofenac treatment and its regulation is associated with oxidative stress and programmed cell death as was shown in previous studies with mouse and rat [71, 72]. In kidney the mitochondrial reductase CYB5B, CYB5R2 and the fatty acid hydroxylase CYP4V2 were significantly repressed after diclofenac treatment. Note, CYB5B and CYB5R2 where shown to function in androgenesis in Leydig cells of rats [73], and the significant reduction in testis and epididymis tissue weight overserved in the present study agrees with the aforementioned study. Furthermore, these monooxygenases are involved in the fatty acid metabolism and regulation of cholesterol synthesis [74].

We recently reported the complex pro- and anti-inflammatory reactions in mice after diclofenac treatment, and it appears that the immune system is generally involved in the toxicity of several NSAIDs [22, 75–77]. Given that the liver is enriched with macrophages and other cells of the innate and adaptive immune system such as sinusoidal endothelium and hepatic stellate cells with functions in antigen presentation and cytokine release [19, 78, 79] we were particularly interested in investigating immune mediated reactions of hepatic injury. We observed a range of cytokines, chemokines and TNFα mediated causes of liver injury. Specifically, the pro-inflammatory chemokine CXCL12 and its receptor CXCR4 and CXCR7 was shown to play a major role in cancer progression [80]; however, this chemokine also induces CD4^+^ T cell activation and regulates the trafficking of inflammatory cells (neutrophils, leukocytes, B-lymphocytes) and apoptosis to sites of inflammation [81, 82]. IL-1ß is another potent pro-inflammatory cytokine; it likewise activates and recruits leukocytes, especially neutrophils and induces expression of acute phase proteins including SAA1 to SAA3 to cause liver injury by releasing proteases during diclofenac treatment [22, 83, 84]. Increased expression of IL-1ß was reported for different NSAID including diclofenac treated mice [17, 22, 85, 86]. Additionally, we observed repressed IL-33, and this member of the IL-1 superfamily stimulates production of type 2 cytokines by T helper cells during inflammation [87]. Additionally, the TNF family member TNFSF11 (also known as RANKL) was significantly up-regulated, and this cytokine is known to be involved in T cell-dependent immune response and hepatic inflammation by activating RANK/RANKL signaling [88, 89]. Moreover, TNFSF15, which we found to be up-regulated upon diclofenac treatment, is associated with T helper (Th1, Th2 and Th17) cell-mediated immune response [90, 91]. The significant repression of TNFSF10 or TRAIL to 30% of controls signifies adaptive response to cytokine induced apoptosis as observed in the present study (Figure 5) and in cultures of human intestinal epithelial cells [92]. Collectively, the above mentioned cytokines and chemokines bind and activate their respective receptors to promote inflammatory responses in the liver. In addition, a highly significant >30 fold induction of LPS-binding protein (LBP, an acute phase protein) and of the Toll- like receptor 4 (TLR4) (>2 fold) was observed. The role of these proteins in hepatic injury by mediating LPS-induced inflammatory cascades has been the subject of several independent reports [93–96].

Next to the liver we considered inflammatory responses in kidney and observed significant regulation of cytokines and chemokines including CXCL14, CXCL16, CCL20, and IL-34 and its receptor IL10RB. The chemokines and their receptors regulate the trafficking of immune-competent cells to sites of inflammation in organs such as intestines, liver, lungs and kidneys. In this regard the chemokine CXCL14 (breast- and kidney-expressed) is of particular importance for its ability to stimulate proliferation of tissue macrophages; its increased expression promotes T-cell activation and Th1 differentiation as well as the trafficking of macrophages, dendritic cells and natural killer cells to sites of inflammation [97–99]. The chemokine CXCL16 was also significantly up-regulated (12 fold) in response to diclofenac treatment and plays a pivotal role in the pathogenesis of renal injury and fibrosis by regulating macrophage and T-cell infiltration and myeloid fibroblast accumulation in kidney [100, 101]. Several factors including TWEAK, ANG II and inflammatory cytokines were reported to induce the expression of CXCL16 during renal inflammation [101–103]. Additionally, chemokine (C-C motif) ligand 20 (CCL20), also known as macrophage inflammatory protein-3α, is mainly expressed in activated cells of inflamed tissues, and the ligand-receptor pair CCL20-CCR6 attracts immature dendritic cells, effector/memory T cells and B cells to the site of renal inflammation [104, 105]. The increased expression of IL-34 is testimony to a coordinate response by regulating the activation and differentiation of monocytes and macrophages in the pathogenesis of inflammation [106, 107]. Expression of IL10RB was also increased (5-fold) in response to diclofenac treatment; as a co-receptor, IL10RB interacts with heterodimeric receptor complex of IL-10 cytokine family members and activate JAK/STAT signaling pathway [108–110]. An activated JAK/STAT signaling was shown to play a decisive role in inflammatory cell infiltration, accumulation of an extracellular matrix and development of tubulointerstitial fibrosis of the kidney [111]. Moreover, several studies reported this pathway to be implicated in renal diseases including diabetic nephropathy [112, 113], glomerular nephritis [114, 115] and renal ischemia/reperfusion (I/R) injury [116]. Thus, the increased expression of these cytokines and chemokines in response to diclofenac treatment promotes immune-mediated glomerular injury as was summarized in a review [117].

In kidney of diclofenac treated animals, significant up-regulation of the genes coding for C1Q, C1S, C1R, C2, C3, C4, CFB and CFI was observed. The activation of complement factors is part of the innate immune system but can also be activated as a result of I/R injury induced in renal cells [118, 119]. Specifically, diclofenac treatment induced transcriptional activation of C1Q, C1S, C1R and C2 which are implicated in the activation of classical pathway and are ultimately responsible for the activation of C3 convertase, a key molecule of the alternative pathway by cleaving the C3 molecule [120]. As was observed in hepatic tissue an increased expression of C3, C5 and C9 facilitates the formation of membrane attack complex (MAC) to cause cell lysis and resulting in tubulointerstitial injury after reperfusion [121, 122]. The release of pro-inflammatory cytokines/chemokines and ROS by anaphylatoxins (C3a and C5a), i.e. the cleavage of C3 and C5 molecules, perpetuate the immune response and injury [122]. Our study highlights activation of the complement system as a mechanism of renal toxicity as was reported for patients who developed renal papillary necrosis and other injuries in response to diclofenac medication. Furthermore, the reduction in prostaglandin formation and associated renal blood flow causes ischemia and hypoxic injury. Moreover, the expression of several membrane complement regulatory proteins known to inhibit complement activation was observed. We found significant induction (3-fold) of CD46, a membrane cofactor protein which regulates the inactivation of soluble factor I mediated complement cascades to protect renal cells from damage [123, 124].

Apart, pathway analysis revealed activation of MAPK signaling molecules in the liver of diclofenac treated animals most notably ARHGEF6, CDK1, CRKL, DDIT3, DUSP10, HSPA8, IL-1ß, IL1R1, IL1R2, MAP2K7, PAK2 and TNFSF11. Their regulation can be triggered by a variety of extracellular stimuli including mitogens, cellular stress or pro-inflammatory cytokines and play a crucial role in cellular activities such as gene expression, proliferation, differentiation, cell cycle control, apoptosis and inflammation-mediated hepatotoxicity [125–128].

### Gene regulatory networks in the liver

The network analysis defined several master regulators, i.e. THBS1, TNFSF11, S100A8, IL17A, IGFBP-1 and SOD1 after low and high dose diclofenac treatment in liver. THBS1 is an adhesive glycoprotein and facilitates platelet aggregation and cell matrix interaction. It was significantly up-regulated (2-fold) in response to diclofenac treatment and is released early during the acute phase response. Importantly, THBS1 activates transforming growth factor (TGF)-β, a key molecule in the development of liver fibrosis [129–131] and inflammatory renal diseases [132–134]. Several studies reported increased expression of THBS1 in hepatic fibrosis and hepatocellular carcinoma [130, 135, 136], and a recent review summarizes the function of THBS1 and its activity via CD47 in inhibiting NO, cGMP, cAMP and VEGF signalling to promote thrombosis and to decrease tissue survival [137]. These findings suggest an important role of THBS1 in liver diseases; its inhibition improves liver regeneration and therefore represents an interesting drug target to mitigate hepatic injury [138].

TNFSF11, also known as receptor activator of NF-κB (RANK) is another master regulator and functions in the regulation of T cell-dependent immune response [139]. TNFSF11 instructs activation of NF-κB, JNK, p38 and ERK1/ERK2 and promotes multiple cellular events including apoptosis, proliferation, survival and differentiation [140]. NF-kB activation is a major molecular response in I/R injury, and the interaction of RANK with its ligand (RANKL) promotes its activation and hepatic inflammation [89]. Note, RANKL and RANK were modestly but significantly induced by 50% and a similar 1.5-fold induction was observed for TNFRSF11B (osteoprotegrin), a decoy receptor of RANKL after repeated diclofenac treatments for 28 days. In mice, RANKL was shown to protect against hepatic I/R injury; therefore, it is tempting to speculate that oxidative stress induced by diclofenac treatments elucidated an adaptive response to reduce liver injury [89]. The acute phase protein S100A8 was identified as another master regulator and was shown to be strongly induced in various pathological conditions associated with inflammation [141, 142]. S100A8/9 amplifies pro-inflammatory signals such as IL-6, TNFα, CXCL1 and CXCL2 which were induced up to 12 and 3-fold, respectively, in liver of diclofenac treated dogs. Furthermore, increased transcript expression of S100A8 was observed in the livers of mice after treatment with a range of drugs, e.g. acetaminophen, diclofenac and halothane [143], and a similar 2-fold induction of this acute phase reactant was determined in the present study. In addition, we identified IL-17A as master regulator after low dose diclofenac treatment, and this cytokine is produced by CD4+ Th- cells (Th17). Increased expression of plasma IL-17 levels was also reported after diclofenac treatment of mice [22]. Consistent with the broad function of its receptor, IL-17 up-regulates expression of several pro-inflammatory cytokines, chemokines and metalloproteases and plays a critical role in neutrophil recruitment, angiogenesis, inflammation and autoimmune diseases [144]. Collectively, IL-17 plays an essential role in the pathogenesis of immune-mediated liver injury induced by alpha-naphthyl isothiocyanate [145], carbamazepine [146], diclofenac [22] and halothane [147].

Furthermore, we determined SOD1 as a master regulator in the liver of diclofenac treated dogs. SOD1 is one of the major intracellular antioxidant enzymes and of critical importance in antioxidant defence by catalyzing the conversion of superoxide free radicals to hydrogen peroxide and molecular oxygen [148]. Earlier studies reported ROS to be significantly increased in hepatocytes treated with diclofenac [149]. We observed decreased SOD1 expression in harmed hepatocytes to aggravates liver injury and similar findings had been reported by others [72, 150–152]. Another master molecule significantly up-regulated in response to high dose diclofenac treatment was IGFBP-1, a member of structurally related soluble proteins that binds and modulates the actions of insulin growth factors 1 and 2. Several proteins of the IGFBP family are potent inducers of the apoptotic cell death pathways; however, IGFBP-1 acts as a pro-survival factor by modulating the mitochondrial apoptotic pathway in the liver [153]. IGFBP-1 is an early-response factor rapidly induced by glucocorticoids, pro-inflammatory cytokines and ROS [154, 155] as well as other stress-related events [156, 157] with hepatic IGFBP-1 mRNA expression being also increased in alcoholic liver disease, cirrhosis [156], hepatocellular carcinomas [158] and steatosis [159]. Elevated IGFBP1 serum levels are also a prognostic factor for worse outcome in critically ill patients [160].

### Gene regulatory network in the kidney

Network analysis of LD and HD diclofenac treated dogs revealed LEPR, NR1H2, CFI, SERPING1, CD74 and CFB as master regulatory molecules in kidney. Intriguingly, at the low dose all master regulators were repressed in expression, however, at the 3 mg/kg/day dose CD74 and complement factor B were induced >5-fold. The leptin receptor plays a key role in energy expenditure and food intake but also in the control of inflammation by influencing monocyte/macrophage-mediated responses [161, 162]. Several studies suggest leptin to be a pro-inflammatory molecule whereby the leptin receptor directly or indirectly modulates signaling pathways involved in kinase-induced phosphorylation by JAK2/STAT3, ERBB2, ERK, IRSl and Rho/Rac [163]. Among its versatile functions leptin induces the expression of TGF-β1 in glomerular endothelial cells and promotes G_1_ progression of the cell cycle through cyclin-dependent kinases [164]. Leptin induces proliferation, differentiation and function of haemopoietic cells including T cells [165] and plays a role in renal fibrosis [166]. It also activates human leukocytes via receptor expression on monocytes [167] and induces oxidative stress [168]. Collectively, leptin is a mediator of the immune response [169], and in our recently published study with diclofenac treated mice we evidenced induction of the leptin receptor by immunohistochemistry, even though transcriptional responses are opposite when the two species are compared [17].

Furthermore, computational analysis revealed the nuclear receptor NR1H2 (also known as LXRß) as another master molecule. This receptor functions a key regulator of lipid metabolism, immune and inflammatory signalling in macrophages [170–172]. It was shown that LPS and inflammatory cytokines repress the expression of LXRs and its target genes (i.e. ABCA1 and ABCG4) to cause acute renal injury through lipid accumulation in kidney cells [173, 174]. Moreover, the complement factor I and B were identified as master regulators in kidney of diclofenac treated dogs, and the kidney was shown to be susceptible to complement-mediated inflammatory injury in different animal models [175]. Interestingly, diclofenac treatment elicited activation of the alternate pathway in liver but the classical pathway in kidney with strong induction of IgM and IgG in liver (as evidenced by IHC) and up to nearly 9-fold induction of individual components of the classical pathway (e.g. C1RA (8.5-fold), C1S (7.5-fold), C3 (6-fold), CFB/C2 (5.5-fold), CFI and C4 (approx. 2.5-fold)) in kidney. An activation of the two master regulators CFI and CFB are considered to be culprits of immune complex mediated glomerular tissue injury [176–179].

The present study also highlights SERPING1 (also known as C1INH), an extracellular matrix protein, as a master molecule that regulates complement activation and leukocyte trafficking to prevent tissue damage induced by inflammatory cytotoxic reactions. It was reported that the increased expression of this inhibitor at inflammation sites protects the kidney from complement-mediated injury by non-specific defence mechanisms during renal I/R injury and fibrosis [122, 180]. Lastly, network analysis revealed CD74, a single-pass type II transmembrane glycoprotein that functions as a chaperon in the control antigenic peptide loading as a master regulator. A functional heteromeric MIF factor is formed by CD74 and CXCR4 [181] and its associated protein complexes (i.e. MIF, CD44, and CXCR) regulate various signal transduction pathways during immune-mediated inflammatory conditions [47, 182–185]. High glucose, metabolic mediators of injury, nephrotoxins and cytokines increase the expression of CD74 in renal cells to cause kidney injury [186] and is also highly up-regulated in clear cell renal cell carcinoma [187]. CD74 also regulates B-cell development and proliferation, survival and inflammatory mediators in non-immune cells [183, 188] and the performed immunohistochemistry (see Figure 8) clearly evidenced high expression of CD74 of activated leukocytes in hepatic sinusoids of diclofenac treated animals.

### Co-occupancy of transcription factor binding sites in liver and kidney

To further interrogate the constructed networks, TFBS at gene specific promoters for immune, stress, inflammatory, hypoxia, cytokine stimulus and acute-phase responses were analyzed, and the related TFBS modules/composite modules were computed for low and high dose diclofenac administered liver and kidney. Specifically, E2F1, a member of the E2F transcription factor family, was significantly repressed in expression in liver and kidney of low dose treated animals. This transcription factor endorses cell cycling and regulates the expression of genes involved in cell proliferation, differentiation, apoptosis and DNA repair [189]. The increased expression of E2F1 transcription factor can alter TGF-ß mediated growth inhibitory effects by inducing cell cycle arrest and apoptosis as a result of liver and kidney injury [190–192] but also contributes to cancer development [193, 194]. E2F1 was also identified as a novel regulator of immune responses to LPS treatment of mice [195, 196]. Furthermore, the transcription factor AIRE is part of the hepatic composite module and a key regulator in autoimmune diseases [197]. It was shown that the CARD domain of AIRE forms a protein complex through dimerization that triggers apoptosis, inflammation and innate immune recognition [198]. AIRE-induced stress response and cell death could be a result of the simultaneous transcription of a high number of genes including T-cell receptors [197].

Additionally, low dose diclofenac treatment revealed hepatic P53 which is typically regulated in response to DNA damage and hypoxia and mediates a variety of anti-proliferative processes [199, 200]. As a stress-activated transcription factor, P53 regulates the expression of numerous downstream genes which could promote apoptosis via cell cycle arrest [201] and in some cases represses the expression of genes to protect cells from cytotoxicity [157]. Another transcription factor of the composite module is ETS1, and the ETS family of transcription factors are critical regulators for endothelial cell proliferation, differentiation, migration, apoptosis and cell-cell interactions [202]. ETS1 is implicated in the cell response to stress [203], and an increased expression of this factor regulates various metabolic and oxidative stress pathways in several cancer models [204, 205] but mediates vascular inflammation as well [206]. Moreover ETS1 interacts with P53 to activate p53-responsive pro-apoptotic genes including BAX, DR5 and FAS [207]; however, in the present study the genes were mostly unchanged in liver and kidney of diclofenac treated animals.

In addition, KLF6 was identified as part of the composite module, and this zinc finger protein influences a wide range of processes including cellular differentiation, cell cycle progression, oxidative stress and apoptosis as well as adipogenesis and glucogenesis [208–210]. Among the KLF proteins, isoforms 1-3, 6, 11 and 13 play major roles in hematopoiesis and immunity [211] whereas KLF4 and KLF6 regulate pro-inflammatory signaling in macrophage [62, 212]. In the present study most of the KLF-transcription factors were unchanged or slightly (<2-fold) regulated in liver and kidney; however, KLF6 transcript was induced >3-fold and the protein was markedly expressed (Figure 13). Increased KLF6 expression augments enhanced collagen 1 and TGFß1 expression to result in liver fibrosis [213], and overexpression of KLF2 induces expression of the fatty acid translocator CD36 [214] whereas KLF15 plays an essential role in ER stress-mediated insulin resistance in the liver [215]. Of note, CD36 was increased by 2-fold and histopathology revealed hepatic steatosis (Figure 5 and Table 5).

Interrogation of promoter sequences of regulated genes in kidney revealed MYOD as enriched transcription factor. Pro-inflammatory cytokine mediated hypoxia and stress response signaling repress the expression of MYOD and this was also observed in the present study. MYOD was reported to induce cell cycle arrest through activation of E2F and P21 mediated activities [216, 217].

Additionally, the constructed composite module identified cAMP response element binding protein (CREB) as significantly enriched, and this protein plays an important role in the regulation of T- and B- lymphocytes and Th-cells [218]. A variety of growth factors and inflammatory/stress signals induce CREB1 activation though diclofenac treatment caused a minor 1.6-fold increased transcript expression of this factor. Collectively, CREB participates in the transcriptional regulation of pro-inflammatory and/or immune-related genes as observed in the present study and as reported by others [218, 219].

Finally, GATA factors though unchanged in expression were enriched in the constructed composite module. Of note, GATA1 plays a major role in the hematopoietic and immune system in mice [220] and regulates pro- and anti-inflammatory chemokine receptors in the orchestration of the inflammatory response [221].

In conclusion, a mechanism of allergic liver injury is proposed that results in lobular inflammation, inflammatory cell infiltrates, hepatocellular damage and granulomatous hepatitis. Diclofenac causes unprecedented induction of MPO in macrophages and oxidative stress that is associated with reactive metabolite production and the formation of diclofenac adducts. These are phagocytized by APCs and other cells of the mononuclear phagocytic system and through interaction with MHC molecules elicit a T- and B-cell response. The highly induced mast cell infiltration and their activation is testimony to an allergic reaction and involve the complement system. Given the observed sequence of liver lesions we consider the findings as relevant for other NSAIDs and may possibly be extended to a general mechanism of allergic liver injury.

## MATERIALS AND METHODS

### Animals

Nine male beagle dogs were purchased from the Marshall Beijing (Marshall Farms Wayao village, Liucun town, Changping District, China). The average body weight was 7.5±0.17 kg. The animals were maintained under controled housing conditions of a temperature of 23±3°C, humidity of 55±10% and with 12-hour light/dark cycle at 150–300 lux and an air ventilation of 10-20 times/hour. The dogs were given dog chow - 300 g (Safe Diet, Scientific Animal Food & Engineering, France) and water *ad libitum*. All animals were acclimatized to their housing for 4 weeks prior to randomization into treatment groups.

### Drug treatment

Diclofenac was purchased from Sigma-Aldrich, Inc (CA, USA) and next to controls (N=3) groups of N=3 animals were administrated orally with either 1 mg/kg/day (LD) or 3 mg/kg/day (HD) for 28 days. The drug substance was loaded into gelatine capsules to achieve individual doses based on body weights and the vehicle control group received one empty capsule per day.

### Clinical pathology

Blood and urine samples were collected during pre-treatment and treatment periods (Day 1, 15 and 28) for evaluation of haematology, serum chemistry and urinalysis. The animals were fasted overnight prior to blood or urine collection. The blood samples were collected from the cephalic vein. For haematology, 0.5 mL of the blood samples were placed into the tubes containing EDTA-2K and the parameters white blood cells (WBC), red blood cells (RBC), haemoglobin concentration (HGB), hematocrit (HCT), platelet (PLT) and differential leukocyte count were measured with an ACL 9000 analyser (Instrumentation Laboratory, Italy). In addition, 2.0 mL blood samples were drained into tubes without an anticoagulant and stored at room temperature for 90 minutes and then centrifuged at 3000 rpm for 10 minutes at room temperature to obtain serum. Aspartate aminotransferase (AST), alanine aminotransferase (ALT), alkaline phosphatase (ALP), gamma glutamyl transferase (GGT), total bilirubin (TBIL), triglyceride (TG), glucose (GLU), albumin (ALB), total protein (TP), creatine phosphokinase (CK), blood urea nitrogen (BUN) and creatinine (CREA) were assayed on a Toshiba 200FR NEO (Toshiba Co., Japan). Furthermore, the serum and urine electrolytes (Na, K, Ca and Cl) were analyzed on a Toshiba 200FR NEO (Toshiba Co., Japan).

### Histopathology

Using standard operating procedures of the laboratory a range of stains were employed to evaluate the liver morphology of control and diclofenac treated dog liver and kidney and included Hematoxylin and Eosin (H&E), Periodic Acid-Schiff's reaction (PAS), PAS diastase digestion, Elastica van Gieson, silver and Prussian blue, Giemsa and chloroacetate esterase (CAE) stain.

### Immunohistochemistry

Immunohistochemistry was done as described in our recent study [17]. Livers from control and diclofenac treated animals were fixed in 4% buffered paraformaldehyde and embedded in paraffin block using standard protocols of the laboratory. 1 μm thick sections were deparaffinised and rehydrated through a descending alcohol series followed by a 4 min washing step in distilled H2O. Subsequently, antigen retrieval was performed in citrate buffer (pH 6) in a water bath at 98°C for 30 minutes. The ZytoChem-Plus HRP Polymer-Kit of Zytomed Systems (Berlin, Germany) was used for immunohistochemistry. The slides were rinsed with distilled H2O, and after a 5 min incubation step in tris-buffered saline (washing buffer) endogenous peroxidase activity was blocked with 3% peroxidase blocking reagent (Merck, Darmstadt, Germany) for 5 min followed by a second washing step. Thereafter, the sections were blocked for 5 min with protein-block serum free reagent (ZytoChem-Plus HRP Polymer-Kit, reagent 1) and incubated with primary antibodies for 60 min. The antibodies were purchased from diverse vendors and diluted with washing buffer as given in parenthesis (see Table below). The bound primary antibodies or bridging antibodies were incubated with labeled polymer HRP Anti-Rabbit or anti-mouse secondary antibody (ZytoChem-Plus HRP Polymer-Kit, reagent 2) for 20 minutes. Subsequently, the reaction was developed and visualized by use of reagent 3 of the ZytoChem-Plus HRP Polymer-Kit and by placing the slides in a moist chamber at room temperature allowing an incubation time of 30 min.

Finally, the sections were counterstained with hematoxylin for 5 min, washed under running warm tap water for 10 minutes and dehydrated in a cabinet at 60°C for 20 minutes, cover slipped and examined under a light microscope (Nikon Ni-E microscope, Japan). Image capture was done with the Nikon NIS basic research microscopic imaging software version 4.3. The images were converted into tiff files and unless otherwise stated the images were finalized in Adobe Photoshop version CS5.

**Table:** List of antibodies used in immunohistochemistry

Antibody	Vendor	Cat no.	Lot number	Dilution	Antigen retrieval
**C1 INH**	Santa Cruz	Sc-46298	B2706	1:50	pH6
**C3**	Abcam	ab112820	GR119618-2	1:1000	pH6
**CD 74**	Santa Cruz	sc-5441	12807	1:150	pH6
**DEC205 (D-18)**	Santa Cruz	sc-14602	L1813	1:25	pH6
**Factor B**	Santa Cruz	sc-67141	G1808	1:25	pH9
**HIF-1alpha**	Abcam	ab463	GR252860-1	1:50	Pronase
**IgM**	Dako	REF A0425	00086531	1:1000	Pronase
**KLF6**	Biobyt	orb36742	E5903	1:50	ph6
**MPO**	Dako	REF A0398	20001076	1:500	-
**SAA1**	Abcam	ab171030	GR147621-10	1:100	pH6
**SOD 1**	Abcam	ab13498	GR80256-31	1:200	pH6
**SOD 2**	Santa Cruz	sc-30080	J0713	1:250	pH6
**VCAM-1 (D-19)**	Santa Cruz	sc-1504	H1215	1:50	pH6

### RNA extraction

Liver and kidneys were surgically removed from diclofenac treated dogs (1 and 3 mg/kg/day) and control animals and snap-frozen in liquid nitrogen. The liver and kidney samples were stored in a deep freezer until RNA extraction. Frozen liver samples were immediately added to buffer RLT with β-mercaptoethanol and homogenized using a TissueLyser (Qiagen, Hilden, Germany) for microarray analysis. Total RNA from each tissue was isolated and purified using the RNase mini kit (Qiagen) according to the manufacturer's protocol. Furthermore, the concentration of total RNA was measured using NanoDrop spectrophotometer (NanoDrop Technologies, Wilmington, DE) and RNA integrity was determined on a 2100 Bioanalyzer (Agilent Technologies, Santa Clara, CA).

### Microarray experiments and data analysis

250 ng of total RNA was used for each microarray experiment. All steps of cDNA synthesis, biotin labeling, fragmentation, hybridization, staining, washing and scanning was done according to the manufacturer's recommendation (Affymetrix, Santa Clara, CA) and further details are given in [17, 222]. Whole genome expression profiling was performed with the Affymetrix GeneChip canine genome 2.0 and scanned on a GeneChip Scanner 3000 (Affymetrix).

*In vivo* liver and kidney expression data from the low (1 mg/kg/day) and high (3 mg/kg/day) dose and vehicle control groups was processed with the MAS5 algorithm available in the GeneXplain 3.0 platform (http://platform.genexplain.com/bioumlweb). Thereafter, differentially expressed genes (DEGs) were identified from the normalized datasets by applying the hypergeometric statistical analysis with a fold change >1.5 and a p-value<0.05. Genes satisfying those conditions were grouped as up- and down-regulated, and heatmaps for the predicted DEGs were constructed based on the average-linkage hierarchical clustering with Euclidean distance using the software Multi Experimental Viewer (MeV) (http://www.tm4.org/mev.html).

### RT-qPCR

RT-qPCR was performed for 13 genes selected by their level of regulation and included master regulators. The primers were purchased from GenoTech (Daejeon, Korea). Total RNA (2 μg) was reverse-transcribed with SuperScript II (Invitrogen, Carlsbad, CA) using an oligo-dT primer as described by the manufacturer. cDNA samples were stored at –20°C until use. Quantitative real-time RT-PCR was performed in a 20 μl reaction volume containing 0.5 μl (10pM) forward and reverse specific primers, 10 μl of SYBR Green master mix (Applied Biosystems, Carlsbad, California), 2 μl of cDNA and 7 μl of nuclease-free water. The cDNA was amplified using a StepOne and StepOnePlus Real-Time PCR System (Applied Biosystems) following the manufacturer's protocol. The *18S* ribosomal RNA primers were used as an internal control. Note, the primer sequences of all genes investigated are listed in Supplementary Table 11.

### Identification of orthologous genes

As the dog genome is not fully sequenced, orthologs for diclofenac regulated genes were searched against human, mouse and rat genomes using the g:Orth tool in g:profiler server (http://biit.cs.ut.ee/gprofiler/gorth.cgi). This tool retrieves orthologues based on sequence similarity [223] and the identified genes were used for further analysis.

### Functional enrichment analysis

The mapping of DEGs to ontologies included biological processes, cellular components, molecular functions, metabolic and signaling pathways and transcription factor classification. For each ontological term the p-value and the expected and actual number of hits were calculated using the GeneXplain platform version 3.0. A biological process was considered significant at a p-value <0.05 and the terms were used for further analysis. In addition, significantly enriched gene ontology term/pathway networks were visualized with the ClueGO version 2.3.2, a Cytoscape plug-in. Note, ClueGO retrieves the enriched biological term/pathway and gene ontology clusters from precompiled annotation files including GO, KEGG and BioCarta database entries and calculates enrichment score based on the hypergeometric distribution [224].

### Gene/protein interaction networks

Protein-protein-interaction (PPI) networks for DEGs were constructed using the STRING database version 10.0 (http://string-db.org/). The functional relationship among DEGs is considered by retrieving the associations from the publically available biological datasets including high-throughput experimental data, mining of databases and literature and predictions based on genomic context analysis. Furthermore, it calculates the confidence score for each predicted association and constructs protein-protein-interaction (PPI) networks [225].

### Identification of master regulatory molecules

Master regulatory molecules and associated networks of DEGs were identified based on the network analysis tool of the GeneWays database (http://anya.igsb.anl.gov/Geneways/GeneWays.html). The tool is available within the GeneXplain platform version 3.0. A default cutoff (score at 0.2, FDR at 0.05 and Z-score at 1.0) with a maximum radius of 4 steps upstream of an input gene set was used for predicting statistically significant master regulatory genes. The tool automatically extracts, analyze, visualize and integrate molecular pathway data from published peer reviewed articles and more than eight million abstracts [226].

### Promoter analysis

Transcription factor binding sites (TFBS) in promoter sequences of regulated genes were identified within the Genomatix software suite (Munich, Germany). For this purpose DEGs implicated in inflammation, immune, stress, hypoxia, cytokine stimulus, acute-phase responses, cytotoxicity (cell death and apoptosis) and oxidation-reduction processes were retrieved and inspected with the Gene2Promoter tool. The promoter sequences were extracted and transcription start sites (TSS) were automatically assigned on the basis of 5′ cap site database entries. Next, the cis-regulatory binding sites of genomic sequences with a length of -1000 to +100 base pairs relative to TSS were interrogated with the MatInspector tool. Overrepresented (=enriched) TFBS were defined based on statistical significance and by calculating the Z-scores using the vertebrate matrix family library version 9.2 (IUPAC strings and predefined IUPAC libraries) [227].

In addition, composite modules based on the co-occupancy of sets of significantly enriched TFBS in promoters of regulated genes were computed using DEGs as input function. After individual TFBS such modules represent the next level of functional organization. We used the following parameters to predict specific TFBS combinations: The quorum constraint for framework, i.e. minimum number of sequences to contain the common framework was set as 75%, minimum number of TFBSs in a framework 2, variation of distance range 20 bp, minimum distance 10 bp and a maximum distance of 200 bp between TFBS. Eventually the FrameWorker tool listed all modules up to a given number of elements that are found to be common among input sequences.

## SUPPLEMENTARY MATERIALS FIGURES AND TABLES





















## Abbreviations

ADR: Adverse drug reaction
ADME: Absorption, distribution, metabolism and excretion
AKR1C3: Aldo-keto reductase family 1 member C3
ALB: Albumin
ALP: Alkaline phosphatase
ALT: Alanine aminotransferase
APC: Antigen presenting cell
AST: Serum aspartate aminotransferase
BUN: Blood urea nitrogen
CA: Carbonic anhydrase
CAE: Chloracetate esterase
CARPA: Complement activation-related pseudoallergy
CK: Creatine phosphokinase
COX: Cyclooxygenase
CREA: Creatinine
CYP: Cytochrome P450
DAMPs: Danger-associated molecular patterns
DEGs: Differentially expressed genes
DILI: Drug-induced liver injury
EMA: European Medicine Agency
GGT: Gamma glutamyl transferase
GLU: Glucose
GO: Gene Ontology
H&E: Hematoxylin and Eosin
HCT: Hematocrit
HD: High dose
HGB: Haemoglobin
IFN: Interferon
Ig: Immunoglobulin
IHC: Immunohistochemistry
IL: Interleukin
LD: Low dose
LPS: Lipopolysaccharide
LUC: Large undifferentiated cells
MAC: Membrane attack complex
MeV: Multi Experimental Viewer
MPO: Myeloperoxidase
NMDA: N-methyl-D-aspartate receptor
NSAID: Nonsteroidal anti-inflammatory drug
PAS: Periodic acid-Schiff reaction
PLT: Platelet
PPI: protein-protein interaction
PRAC: Pharmacovigilance Risk Assessment Committee
RBC: Red blood cells
ROS: Reactive oxygen species
TBIL: Total bilirubin
TFBS: Transcription factor binding site
TG: Triglyceride
Th: T helper cells
TLR: Toll-like receptor
TNF: Tumour necrosis factor
TP: Total protein
TSS: Transcription start site
VLCFA: Very long chain fatty acids
WBC: White blood cells
