# Supplementary material for: The pathogenesis of diclofenac induced immunoallergic hepatitis in a canine model of liver injury
Source: Oncotarget. 2017 Sep 23;8(64):107763–824. doi: 10.18632/oncotarget.21201 (PMC5746105; doi:10.18632/oncotarget.21201)
Supplement: Supplementary file 1 [file oncotarget-08-107763-s001.pdf]

The pathogenesis of diclofenac induced immunoallergic hepatitis in a canine model of liver injury

SUPPLEMENTARY MATERIALS

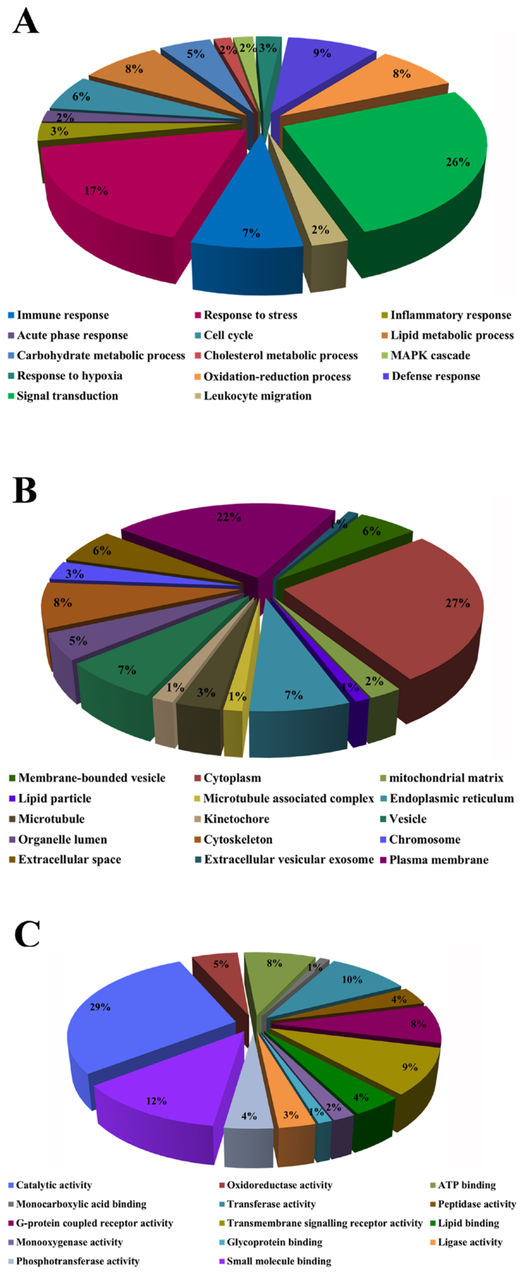

Supplementary Figure 1: GO terms regulated in liver after diclofenac treatment. The gene ontology was analyzed using the GeneXplain platform and a p-value threshold that was set to <0.05. The pie charts depict the distribution of **(Panel A)** key biological processes, **(Panel B)** cellular components and **(Panel C)** molecular functions of low dose diclofenac treatment.

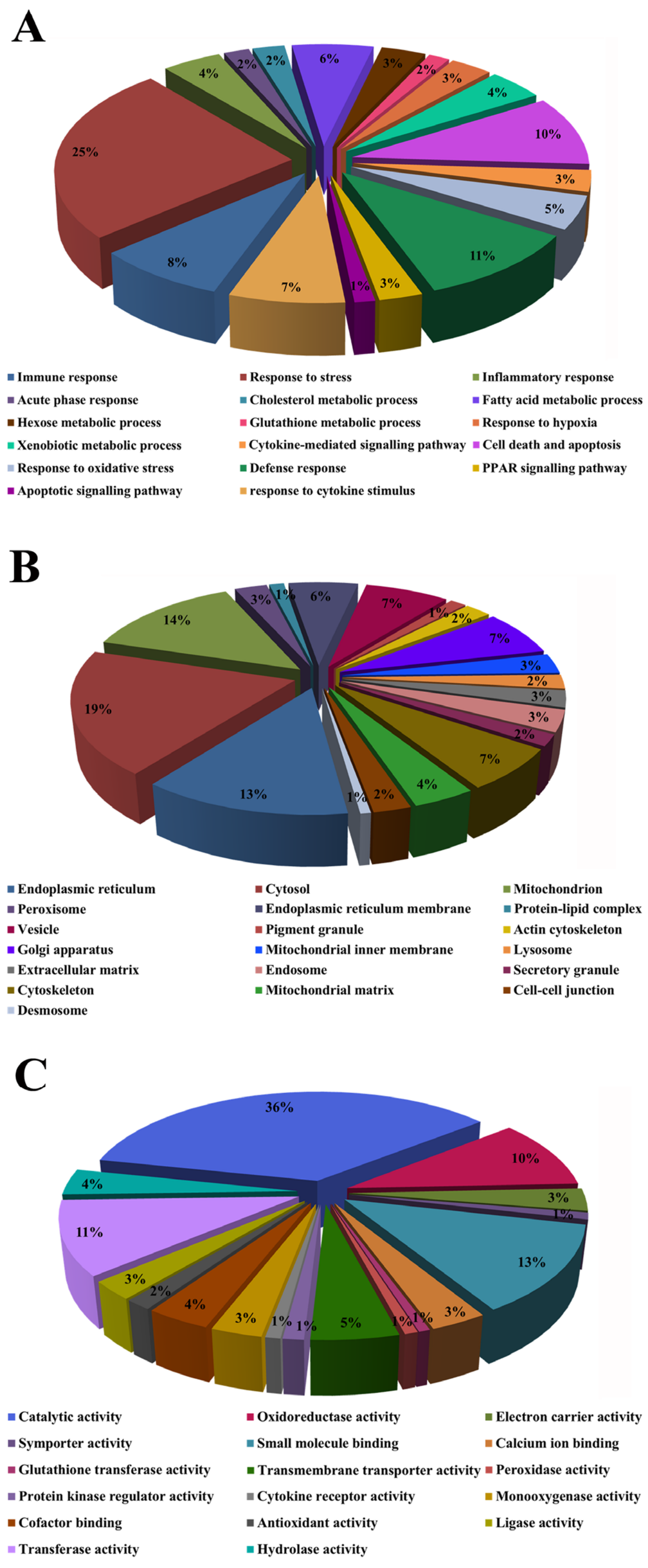

Supplementary Figure 2: GO terms regulated in liver after diclofenac treatment. The gene ontology was analyzed using the GeneXplain platform and the p-value threshold was set as <0.05. The pie charts depict the distribution of **(Panel A)** key biological processes, **(Panel B)** cellular components and **(Panel C)** molecular functions of high dose diclofenac treatment.

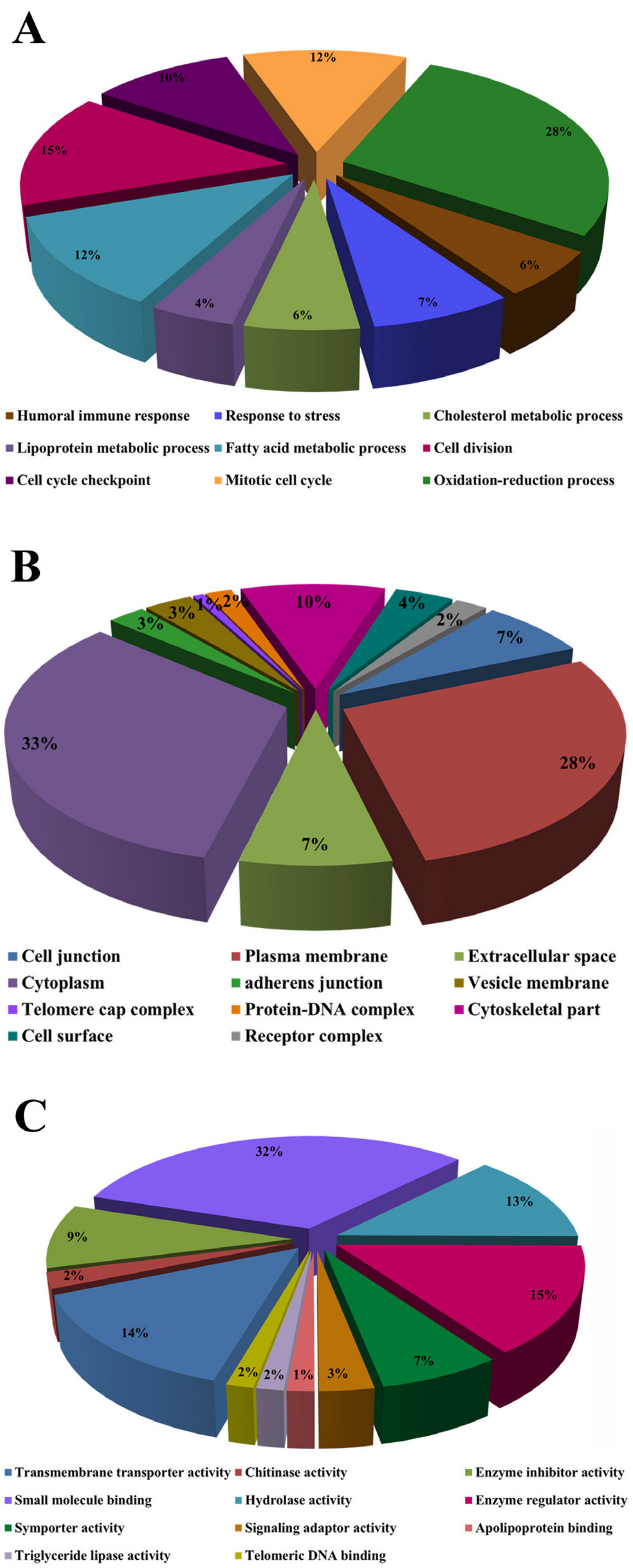

Supplementary Figure 3: GO terms regulated in kidney after diclofenac treatment. The gene ontology was analyzed using the GeneXplain platform and a p-value threshold that was set to <0.05. The pie charts depict the distribution of significant **(Panel A)** biological processes, **(Panel B)** cellular components and **(Panel C)** molecular functions of low dose diclofenac treatment.

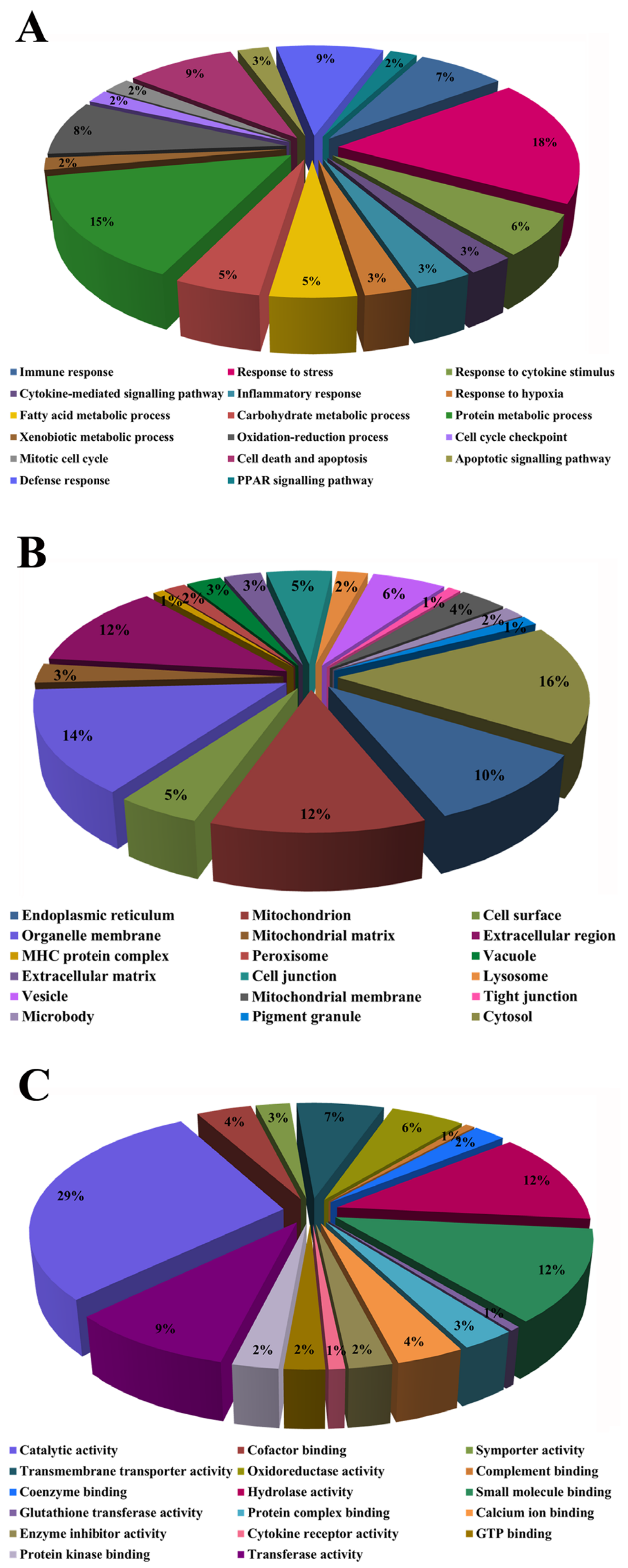

Supplementary Figure 4: GO terms regulated in kidney after diclofenac treatment. The gene ontology was analyzed using the GeneXplain platform and the p-value threshold was set to <0.05. The pie charts depict the distribution of significant **(Panel A)** biological processes, **(Panel B)** cellular components and **(Panel C)** molecular functions of high dose diclofenac treatment.

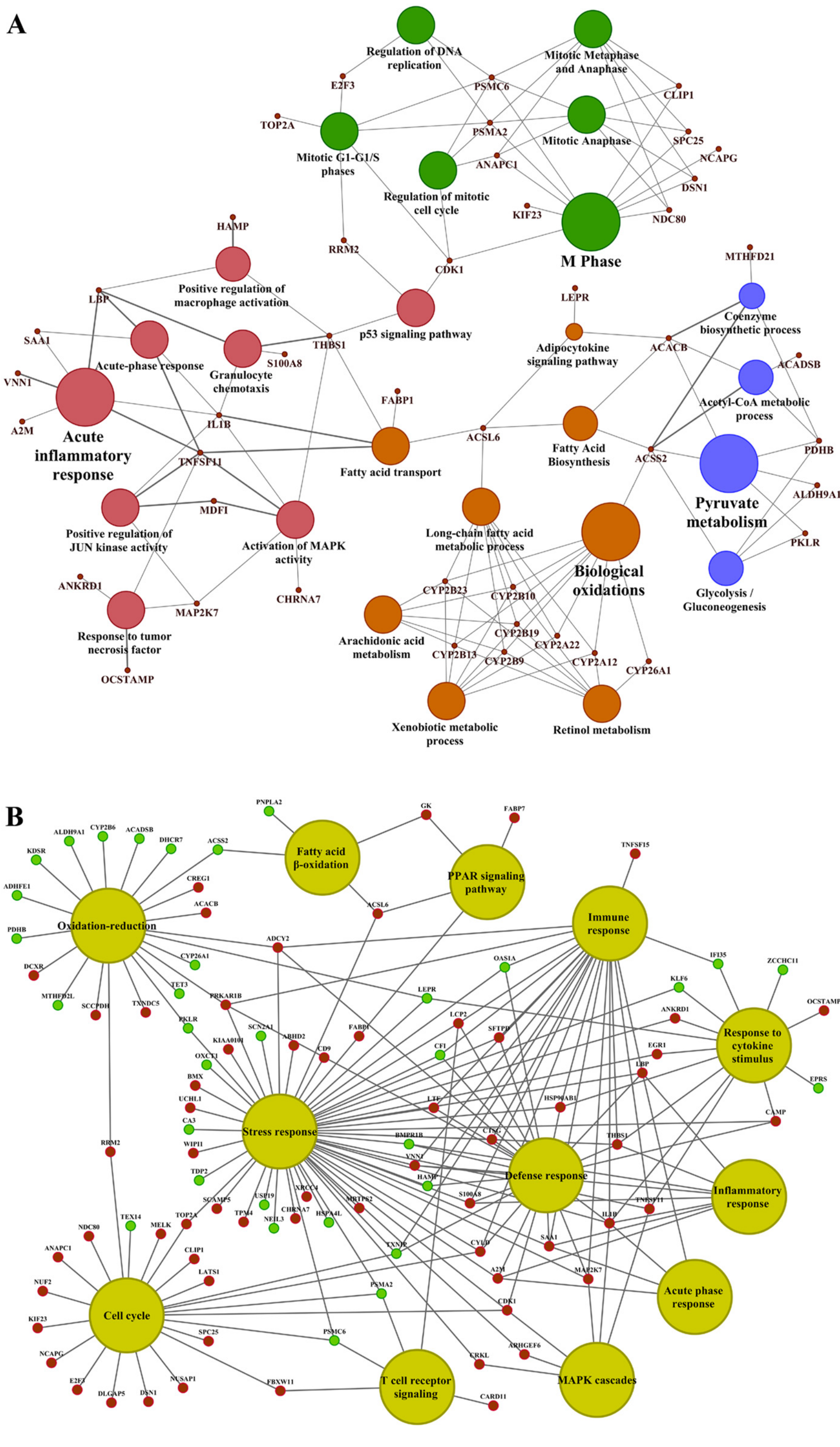

Supplementary Figure 5: ClueGO gene ontology and pathway annotated hepatic networks in response to low dose diclofenac treatment. A visualization of hepatic enriched pathway terms and biological processes were computed with the ClueGo and the GeneXplain software of low dose treated animals. **(Panel A)** Network constructed by the plug-in ClueGO of the Cytoscape software. **(Panel B)** Networks constructed manually using the GeneXplain platform. The enriched biological processes and pathways annotated with the GeneXplain platform and visualized using the Cytoscape software version 3.4. The red and green colored nodes illustrate up- and down - regulated genes, respectively.

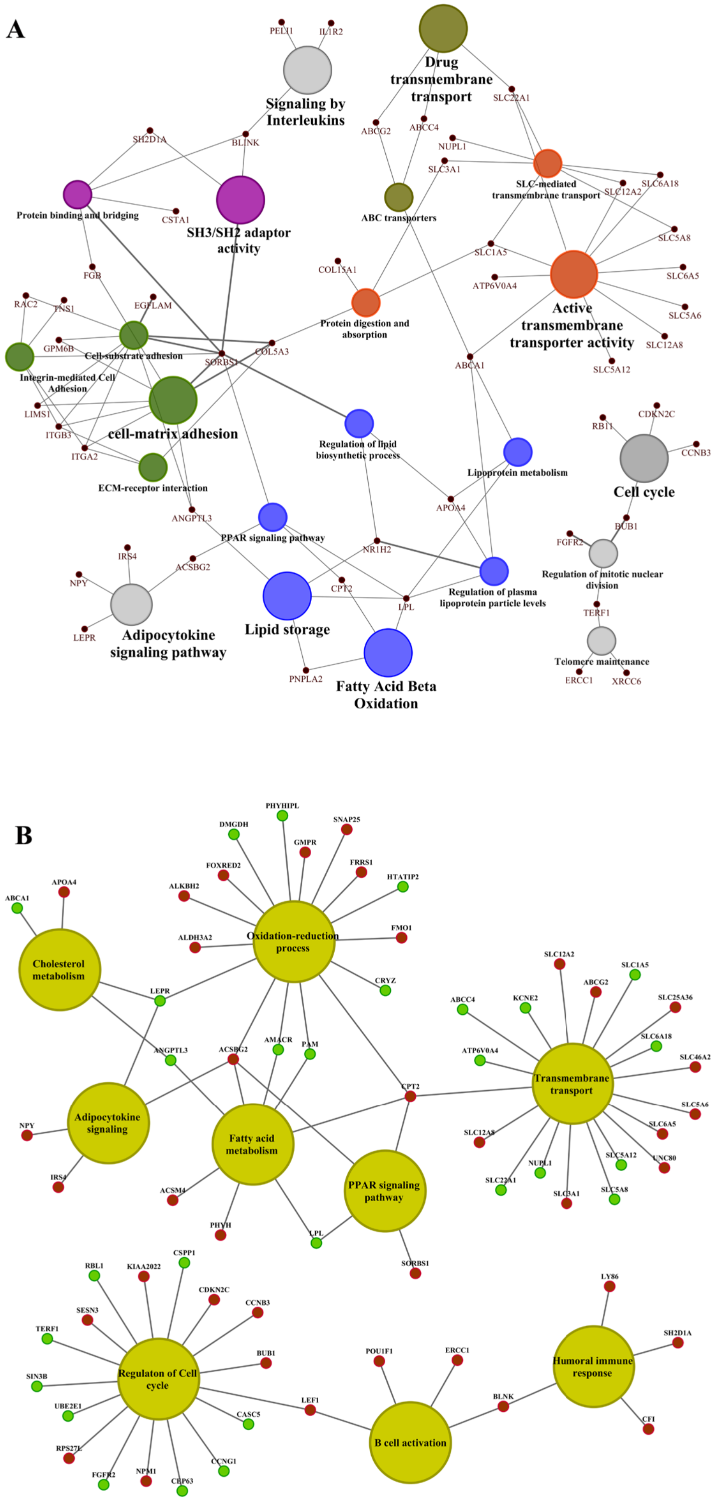

Supplementary Figure 6: ClueGO gene ontology and pathway annotated networks in kidney in response to low dose diclofenac treatment. A visualization of kidney enriched pathway terms and biological processes computed with the ClueGo and the GeneXplain software of low dose treated animals. **(Panel A)** Network constructed by the plug-in ClueGO of the Cytoscape software. **(Panel B)** Networks constructed manually using the GeneXplain platform. The enriched biological processes and pathways were annotated by the GeneXplain platform and visualized using the Cytoscape software version 3.4. The red and green colored nodes illustrate up- and down-regulated genes, respectively.

A

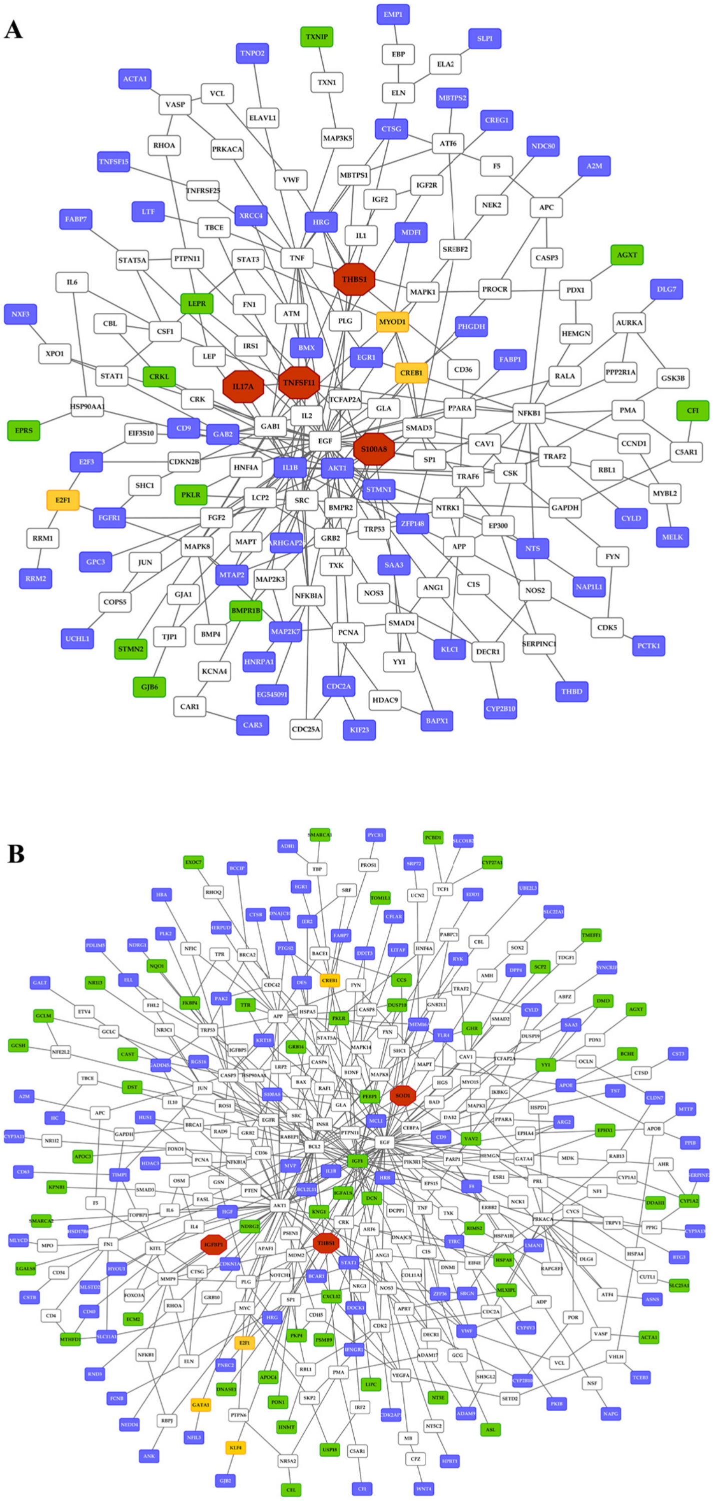

B

Supplementary Figure 7: Master regulatory gene networks in the liver of diclofenac treated animals. Based on interaction information available in the GeneWays database the master regulatory gene networks were constructed and fused using the GeneXplain software. The red, violet, green and yellow colored nodes represent the genes coding for master regulators, DEGs, connecting genes and enriched transcription factors, respectively. **(Panel A)** low dose diclofenac treatment: The network comprised 173 genes of which 63 were significantly regulated. **(Panel B)** high dose diclofenac treatment: The network comprised 347 genes of which 161 were significantly regulated.

A

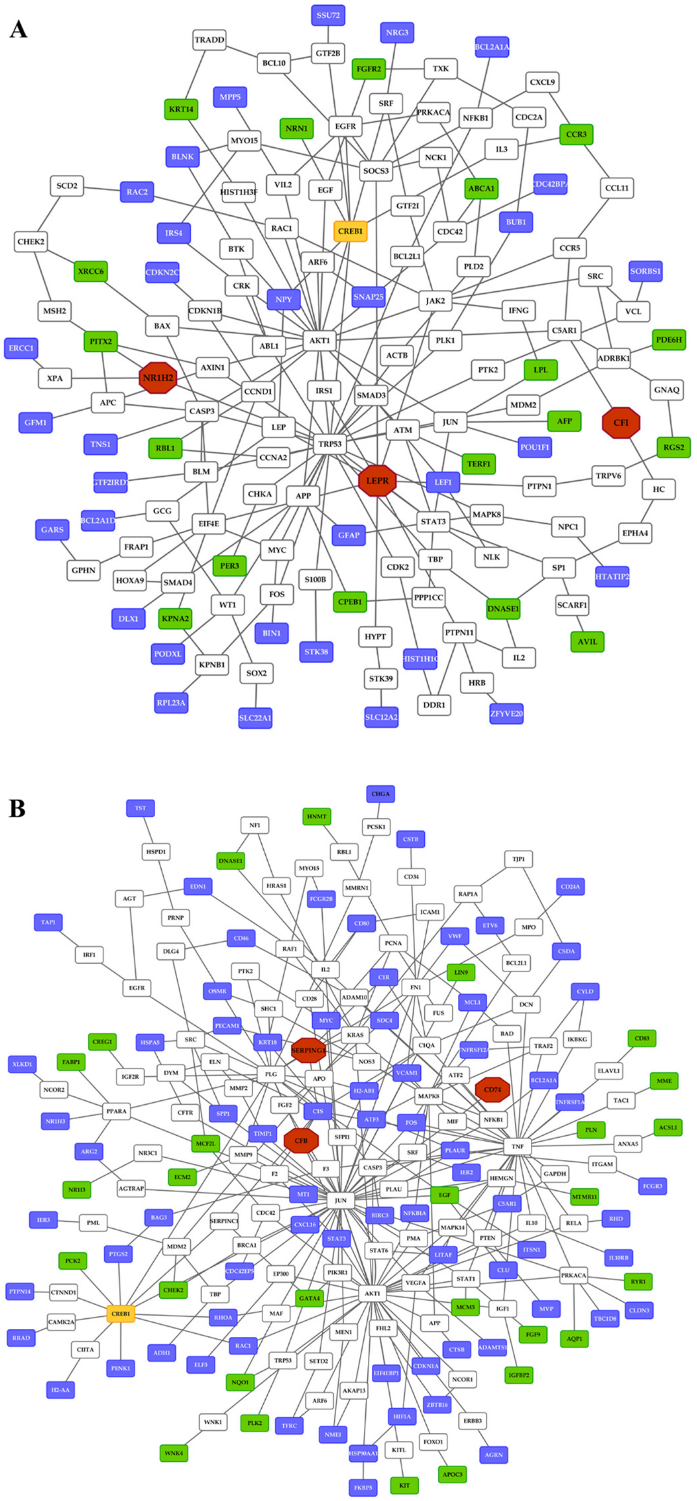

B

Supplementary Figure 8: Master regulatory gene networks in kidney of diclofenac treated animals. Based on the interaction information available in the GeneWays database the master regulatory gene networks were constructed and fused using the GeneXplain software. The red, violet, green and yellow colored nodes represent the genes coding for master regulators, DEGs, connecting genes and enriched transcription factors, respectively. **(Panel A)** Low dose diclofenac treatment: The network comprised 146 genes of which 52 genes were significantly regulated. **(Panel B)** High dose diclofenac treatment: The network comprised 210 genes of which 104 genes were significantly regulated.

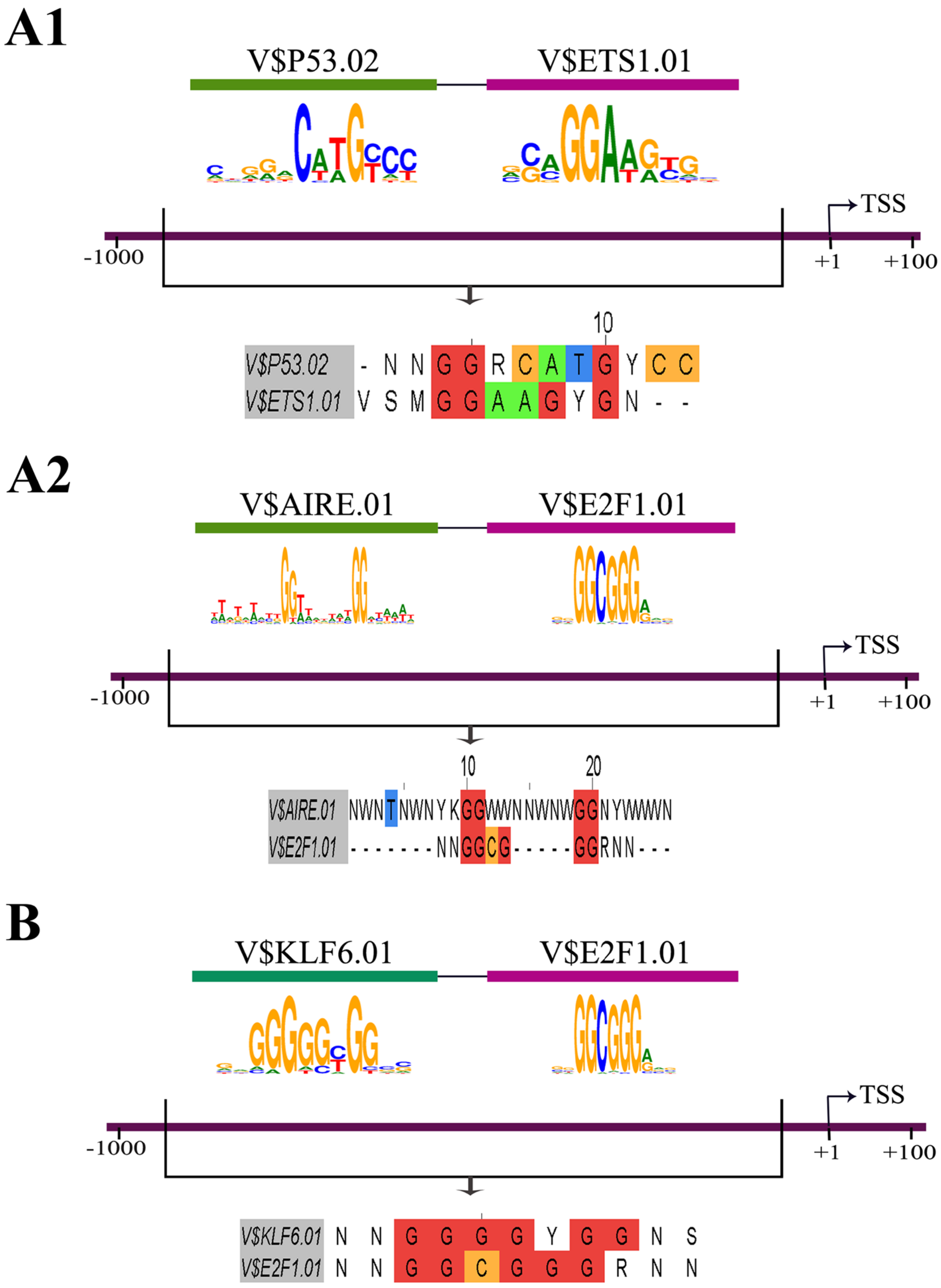

Supplementary Figure 9: Composite module searches to predict gene regulation in the liver of diclofenac treated animals. Significantly regulated DEGs coding for immune, stress, inflammation, hypoxia, cytokine and acute-phase responses were interrogated using the Genomatix FrameWorker software (version 5.4). Transcription factor binding sites at gene specific promoters with the length of -1000 to +100 base pairs relative to TSS were analyzed. The sequence alignment defines the overlapping regions of consensus sequences specific for TFBS. **(Panel A1 and A2)** Composite modules of co-bound transcription factors at promoters of low dose diclofenac induced DEGs. **(Panel B)** Composite module of regulated DEGs after high dose diclofenac treatment in liver.

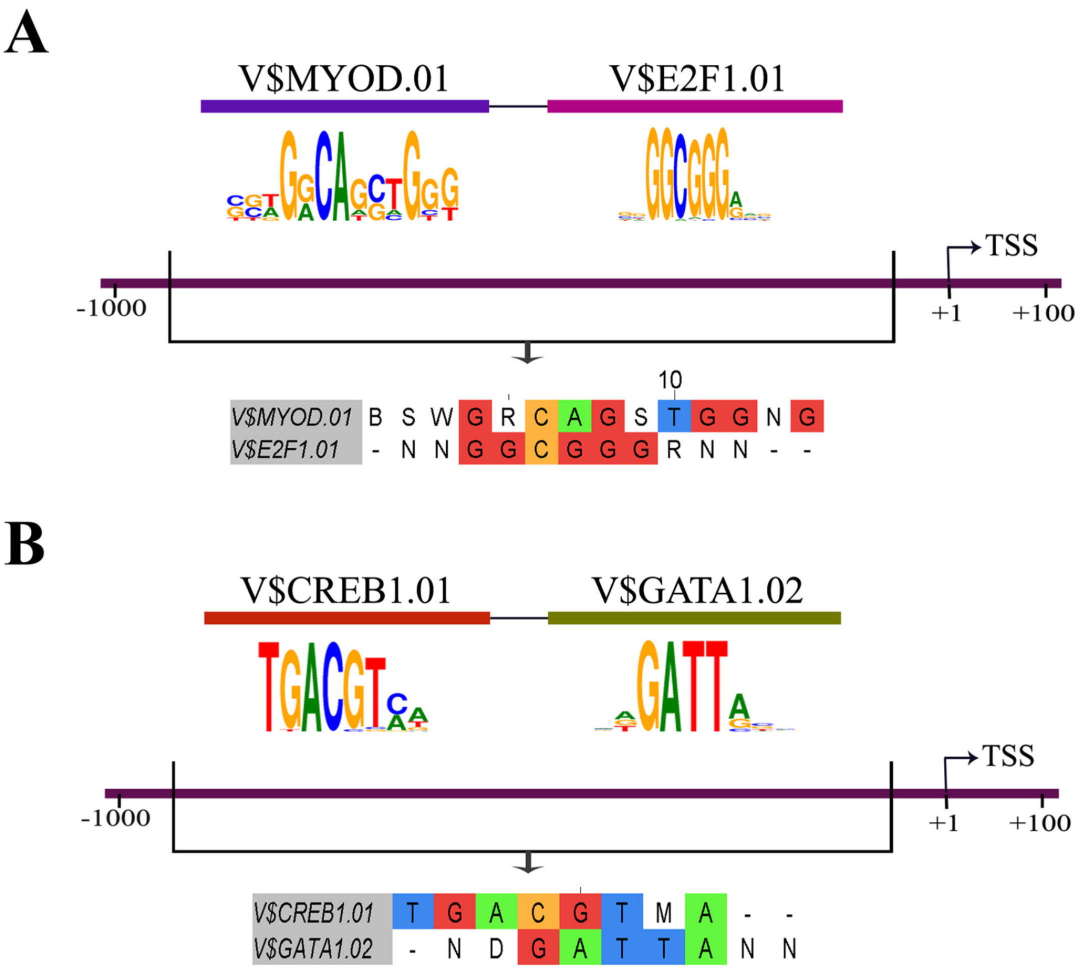

Supplementary Figure 10: Composite module searches to predict gene regulation in kidney of diclofenac treated animals. Cis-regulatory modules at gene specific promoters of DEGs with the length of -1000 to +100 base pairs relative to TSS were interrogated using the Genomatix FrameWorker software (version 5.4). The sequence alignment defines the overlapping regions of consensus sequences specific for TFBS. **(Panel A)** Composite module of low dose regulated DEGs after diclofenac treatment. **(Panel B)** Composite module of high dose regulated DEGs after diclofenac treatment.

Supplementary Table 1: Glycogen synthesis and glucose metabolism pathway regulated genes in liver and kidney after diclofenac treatment

Gene	Gene description	Liver		Kidney	
		LD	HD	LD	HD
		Fold change (average)±SD			
AGL	4-alpha-glucanotransferase	1.33±0.36	1.01±0.13	1.1±0.06	1.12±0.18
ALDOA	Aldolase, Fructose-bisphosphate A	1.02±0.08	1.68±0.29*	1.02±0.01	1.36±0.22
ENO1	Enolase 1	-1.08±0.06	1.02±0.09	1.07±0.01	1.26±0.23
GAPDH	Glyceraldehyde-3-phosphate dehydrogenase	1.05±0.1	-1.09±0.05	1.06±0.05	1.09±0.03
GBE1	1,4-alpha-glucan branching enzyme 1	-1.06±0.11	-2.11±0.5*	-1.03±0.02	-1.24±0.26
GPI	Glucose-6-phosphate isomerase	-1.07±0.06	-1.6±0.62	1.03±0.06	1.1±0.06
GSK3A	Glycogen synthase kinase 3 alpha	-1.1±0.2	1.07±0.07	1.09±0.07	-1.01±0.01
GSK3B	Glycogen synthase kinase 3 beta	1.06±0.06	1.28±0.24	-1.03±0.03	1.37±0.19
GYS1	Glycogen synthase 1	1.01±0.03	-1.15±0.09	1.06±0.05	-1.03±0.1
GYS2	Glycogen synthase 2	1.12±0.13	1.13±0.12	-1.04±0.1	-1.04±0.02
HK1	Hexokinase 1	1.07±0.1	-1.44±0.09	-1.05±0.04	1.07±0.07
PFKL	Phosphofructokinase, Liver	-1.07±0.08	-1.03±0.07	1.07±0.06	1.19±0.17
PGAM1	Phosphoglycerate mutase 1	-1.02±0.02	1.4±0.33	1.05±0.03	1.28±0.19
PGK1	Phosphoglycerate kinase 1	-1.09±0.12	-1.2±0.13	1.13±0.14	-1.66±0.85
PGM1	Phosphoglucomutase 1	-1.16±0.16	-1.66±1.47*	1.23±0.06	1.22±0.18
PGM2	Phosphoglucomutase 2	-1.3±0.23	-1.45±0.34	1.09±0.1	1.18±0.14
PHKA1	Phosphorylase kinase regulatory subunit alpha 1	1.02±0.02	-1.35±0.24	1.15±0.16	-1.11±0.08
PHKG1	Phosphorylase kinase catalytic subunit gamma 1	1±0.01	-1.01±0.12	-1.09±0.11	1.12±0.11
PHKG2	Phosphorylase kinase regulatory subunit alpha 2	-1.09±0.13	1.11±0.1	-1.01±0.02	1.11±0.16
PKLR	Pyruvate kinase, Liver and RBC	-1.78±0.46*	-8.22±3.5*	1.04±0.08	-1.42±0.59
UGP2	UDP-glucose pyrophosphorylase 2	1.11±0.11	-2.05±1.9*	-1.78±0.66	1.03±0.3

* Statistically significant genes.

Supplementary Table 2: Mast cell marker genes

Gene	Gene Description	Liver		Kidney	
		LD	HD	LD	HD
		Fold change (average)±SD			
AOC1	Amine oxidase, Cu containing 1	-1.15±0.07	-1.26±0.01	1.07±0.07	-1.78±0.55*
AOC2	Amine oxidase, Cu containing 2	-1.04±0.05	1.5±0.44	1.35±0.02	1.17±0.08
HDC	Histidine decarboxylase	-1.02±0.12	-1.38±0.24	-1.01±0.1	-1.21±0.04
HNMT	Histamine N-methyltransferase	-1.14±0.32	-2.09±1.79*	-1.29±0.09	-1.98±0.87*
HRH1	Histamine receptor H1	-1.36±0.2	-1.16±0.35	1.11±0.14	1.2±0.08
HRH2	Histamine receptor H2	-1.2±0.17	-1.21±0.1	1.19±0.11	-1.15±0.11
HRH3	Histamine receptor H3	1.23±0.08	1.18±0.19	1.17±0.1	-1.13±0.31
HRH4	Histamine receptor H4	1.1±0.15	-1.05±0.06	1.31±0.12	1.19±0.09
MAOA	Monoamine oxidase A	1.05±0.04	2.16±0.78*	1.09±0.08	1.75±0.34*
MAOB	Monoamine oxidase B	-1.06±0.04	-2.06±0.76*	1.07±0.02	-1.34±0.24

* Statistically significant genes.

Supplementary Table 3: Enriched biological processes in liver and kidney

See Supplementary File 1

Supplementary Table 4: Enhanced signaling pathways in liver and kidney after high dose diclofenac treatment

See Supplementary File 2

Supplementary Table 5: Drug metabolism, solute carriers and transporters

See Supplementary Files 3A and 3B

Supplementary Table 6: Macrophage and granulocyte marker genes in liver and kidney after diclofenac treatment

See Supplementary File 4

Supplementary Table 7: Over-represented transcription factor binding sites in hepatic DEGs after low dose diclofenac treatment

See Supplementary File 5

Supplementary Table 8: Over-represented transcription factor binding sites in hepatic DEGs after high dose diclofenac treatment

See Supplementary File 6

Supplementary Table 9: Over-represented transcription factor binding sites in kidney regulated DEGs after low dose diclofenac treatment

See Supplementary File 7

Supplementary Table 10: Over-represented transcription factor binding sites in kidney regulated DEGs after high dose diclofenac treatment

See Supplementary File 8

Supplementary Table 11: Primer sequences used in real-time RT-qPCR analyses

Gene Symbol	Forward	Reverse
C3	AATCAGGCTCCGATGAGGTG	CCATGTGTCCTTCCCGATGA
CD302	TGGGCCCTGATTACAGAGGA	GAACTGAGGAGAAGCCTGCC
CD74	TAGGACCTGGGCCAAGCTAT	TTGCTGGAGAGAGGGAAGGA
CFB	TGGCTCTCGAAGCCCAATTT	ATGGTATCTGTGCAGGCCAC
CFI	GGGGTTGGTCTTGTTGTTCC	GCTAGGAGGCAGGTGGTTAAT
HIF1A	TGCGCAGAGAAAGCGAAAAA	CGCGTTTCCAAGAAAGTGATGT
IGFBP1	GCTGCACGTTTCAGATGCC	CAGAGGATGGGCGTGTCC
IL10RB	GGAACTGTCTGCCTCAGCAT	GTCTGCTGCTTTCGGAGACT
IL17A	GATCCTGGTTCTGCGAAGGG	ACATGGCGAACAATAGGGGT
IL1R1	GCTGCCTGGGGAATTCTTCT	ATGGCCCCATGTTTCTGCTT
KLF6	TTGGAAGGTCTTTCGAGGGC	GGACACCCGACCATTAGCAT
LBP	TAGTCCCCAAGTTAGCCCGA	ATGAGGGGTGCCAAAGACAG
LEPR	AGTGGCTTAGAATTCCCTCG	ACATACAGCCCTGCGTCATT
NR1H2	GCTGTCTCCGGTTGTTTCCA	GTCCAGAGAACTGGTGGTGG
PKLR	TTCCCCTCTCCATTTCACGC	TGAGAGCATTGAGTGCAGGG
S100A8	CTGACGGAACTGGAGAGTGC	ACCAGGTGTCTGCATCCTTT
SAA1	CAAGAGCCGGTCTATGAGCC	TGTGCTCCTTCTAAACTATTGAGT
SERPING1	GCCTTGTCCTCCTCAATGCT	GTGAAATGGGCCACAGGGTA
SOD1	TTGGAGACCTGGGCAATGTG	CGGCGTTTCCTGTCTGTGTA
THBS1	AACCAATGCTGGTGTTGCAC	TCTTCGCGTTCAGAGGCTAC
TIMP1	AGAGCGTCTGCGGATACTTG	AGCCAGCAGCATAGGTCTTG
TNFSF11	GTACCATGACCGAGGTTGGG	TGGCGAGGTCTCCTGAAGTT
UPP1	ATGCTGCACGAGGTCATCAA	AAACATGGGCGAGAAGGAGG
VCAM1	ACCAACAAGGCACTAGGTTCA	AGCAGTTGCATTTTAGACCACA
GAPDH	GTAGTGAAGCAGGCATCGGA	GTCGAAGGTGGAAGAGTGGG
