# Supplementary material for: The pathogenesis of diclofenac induced immunoallergic hepatitis in a canine model of liver injury
Source: Oncotarget. 2017 Sep 23;8(64):107763–824. doi: 10.18632/oncotarget.21201 (PMC5746105; doi:10.18632/oncotarget.21201)
Supplement: Supplementary file 2 [file oncotarget-08-107763-s002.doc]

**Supplementary Table 3**: **Enriched biological processes in liver and kidney**

| **Low dose_Liver** | | |
| --- | --- | --- |
| **Probe set ID** | **Gene symbol** | **Fold change (average)±SD** |
| **Immune response** | | |
| Cfa.6896.1.A1_at | ADCY2 | 1.94±0.31 |
| Cfa.14495.1.S1_at | CFI | -1.6±0.48 |
| CfaAffx.19298.1.S1_at | CTSG | 1.54±0.25 |
| CfaAffx.15385.1.S1_s_at | CYLD | 1.73±0.46 |
| Cfa.14486.1.S1_s_at | HAMP | -4.99±1.35 |
| CfaAffx.3889.1.S1_s_at | HSP90AB1 | 1.71±0.41 |
| Cfa.16440.1.S1_at | IFI35 | -1.56±0.16 |
| Cfa.3511.1.S1_at | IL1B | 1.82±0.32 |
| CfaAffx.14090.1.S1_at | LBP | 3.21±1.64 |
| CfaAffx.25943.1.S1_at | LCP2 | 1.51±0.31 |
| CfaAffx.21286.1.S1_s_at | LTF | 1.51±0.06 |
| CfaAffx.28061.1.S1_s_at | MAP2K7 | 1.50±0.23 |
| Cfa.21191.1.S1_at | OAS1A | -1.51±0.19 |
| Cfa.19835.1.S1_s_at | PRKAR1B | 1.52±0.16 |
| CfaAffx.26852.1.S1_at | S100A8 | 1.57±0.1 |
| Cfa.3173.2.A1_x_at | SAA1 | 3.09±1.02 |
| CfaAffx.24148.1.S1_s_at | SFTPD | 1.5±0.2 |
| Cfa.21204.1.S1_at | THBS1 | 1.74±0.48 |
| CfaAffx.7934.1.S1_at | TNFSF11 | 1.54±0.18 |
| CfaAffx.5973.1.S1_at | TNFSF15 | 1.59±0.03 |
| CfaAffx.17765.1.S1_s_at | TXNIP | -1.58±0.27 |
| CfaAffx.1247.1.S1_s_at | VNN1 | 1.66±0.08 |
| **Inflammatory response** | | |
| Cfa.6037.1.S1_s_at | A2M | 1.79±0.3 |
| CfaAffx.15843.1.S1_at | BMPR1B | -1.51±0.34 |
| Cfa.3511.1.S1_at | IL1B | 1.82±0.32 |
| CfaAffx.14090.1.S1_at | LBP | 3.21±1.64 |
| CfaAffx.26852.1.S1_at | S100A8 | 1.57±0.1 |
| Cfa.3173.2.A1_x_at | SAA1 | 3.09±1.02 |
| Cfa.21204.1.S1_at | THBS1 | 1.74±0.48 |
| CfaAffx.1247.1.S1_s_at | VNN1 | 1.66±0.08 |
| **Stress response** | | |
| Cfa.6037.1.S1_s_at | A2M | 1.79±0.3 |
| Cfa.436.3.S1_a_at | ABHD2 | 1.74±0.52 |
| Cfa.9802.1.S1_at | ACSL6 | 1.66±0.21 |
| Cfa.6896.1.A1_at | ADCY2 | 1.94±0.31 |
| Cfa.16041.1.S1_at | ANKRD1 | 1.50±0.24 |
| CfaAffx.28963.1.S1_at | ARHGEF6 | 1.6±0.29 |
| CfaAffx.15843.1.S1_at | BMPR1B | -1.51±0.34 |
| Cfa.12505.1.A1_at | BMX | 1.54±0.23 |
| Cfa.4552.1.S1_s_at | CAMP | 2.55±0.86 |
| Cfa.20942.1.S1_s_at | CA3 | -3.44±2.01 |
| Cfa.3854.1.S1_at | CD9 | 1.78±0.59 |
| Cfa.20663.1.A1_at | CDK1 | 1.72±0.24 |
| Cfa.14495.1.S1_at | CFI | -1.6±0.48 |
| Cfa.4753.1.A1_at | CHRNA7 | 1.50±0.31 |
| CfaAffx.23379.1.S1_at | CRKL | -1.58±0.39 |
| CfaAffx.19298.1.S1_at | CTSG | 1.54±0.25 |
| CfaAffx.15385.1.S1_s_at | CYLD | 1.73±0.46 |
| CfaAffx.2782.1.S1_s_at | EGR1 | 2.56±0.72 |
| CfaAffx.11994.1.S1_at | FABP1 | 4.66±2.72 |
| Cfa.14486.1.S1_s_at | HAMP | -4.99±1.35 |
| CfaAffx.3889.1.S1_s_at | HSP90AB1 | 1.71±0.41 |
| Cfa.19944.1.S1_s_at | HSPA4L | -1.50±0.2 |
| Cfa.3511.1.S1_at | IL1B | 1.82±0.32 |
| Cfa.19552.1.S1_at | KIAA0101 | 2.37±0.74 |
| CfaAffx.8966.1.S1_at | KLF6 | -1.51±0.23 |
| CfaAffx.14090.1.S1_at | LBP | 3.21±1.64 |
| CfaAffx.25943.1.S1_at | LCP2 | 1.51±0.31 |
| CfaAffx.28433.1.S1_at | LEPR | -1.53±0.23 |
| CfaAffx.21286.1.S1_s_at | LTF | 1.51±0.06 |
| CfaAffx.28061.1.S1_s_at | MAP2K7 | 1.50±0.23 |
| CfaAffx.20531.1.S1_s_at | MBTPS2 | 1.51±0.25 |
| Cfa.3096.1.A1_at | NEIL3 | -2.15±0.47 |
| Cfa.21191.1.S1_at | OAS1A | -1.51±0.19 |
| Cfa.16830.1.S1_at | OXCT1 | -1.83±1.4 |
| CfaAffx.25935.1.S1_at | PKLR | -1.78±0.46 |
| Cfa.19835.1.S1_s_at | PRKAR1B | 1.52±0.16 |
| CfaAffx.6214.1.S1_at | PSMA2 | -1.56±0.31 |
| Cfa.638.1.S1_at | PSMC6 | -1.55±0.56 |
| CfaAffx.26852.1.S1_at | S100A8 | 1.57±0.1 |
| Cfa.3173.2.A1_x_at | SAA1 | 3.09±1.02 |
| CfaAffx.27544.1.S1_at | SCAMP5 | 1.56±0.11 |
| CfaAffx.17395.1.S1_s_at | SCN2A1 | -1.55±0.28 |
| CfaAffx.24148.1.S1_s_at | SFTPD | 1.50±0.2 |
| CfaAffx.16307.1.S1_at | TDP2 | -1.57±0.3 |
| Cfa.21204.1.S1_at | THBS1 | 1.74±0.48 |
| CfaAffx.7934.1.S1_at | TNFSF11 | 1.54±0.18 |
| Cfa.18946.1.S1_s_at | TOP2A | 2.11±0.78 |
| CfaAffx.24216.1.S1_at | TPM4 | 1.50±0.27 |
| CfaAffx.17765.1.S1_s_at | TXNIP | -1.58±0.27 |
| Cfa.4489.1.S1_at | UCHL1 | 1.67±0.12 |
| Cfa.21495.1.S1_s_at | USP19 | -1.50±0.17 |
| CfaAffx.1247.1.S1_s_at | VNN1 | 1.66±0.08 |
| Cfa.4588.1.A1_at | WIPI1 | 1.67±0.24 |
| CfaAffx.13644.1.S1_at | XRCC4 | 1.50±0.14 |
| **Response to cytokine stimulus** | | |
| Cfa.16041.1.S1_at | ANKRD1 | 1.50±0.24 |
| Cfa.4552.1.S1_s_at | CAMP | 2.55±0.86 |
| CfaAffx.2782.1.S1_s_at | EGR1 | 2.56±0.72 |
| CfaAffx.17037.1.S1_at | EPRS | -1.62±0.56 |
| CfaAffx.3889.1.S1_s_at | HSP90AB1 | 1.71±0.41 |
| Cfa.16440.1.S1_at | IFI35 | -1.56±0.16 |
| Cfa.3511.1.S1_at | IL1B | 1.82±0.32 |
| CfaAffx.8966.1.S1_at | KLF6 | -1.51±0.23 |
| CfaAffx.28433.1.S1_at | LEPR | -1.53±0.23 |
| Cfa.12076.1.A1_at | OCSTAMP | 1.64±0.05 |
| Cfa.21204.1.S1_at | THBS1 | 1.74±0.48 |
| CfaAffx.7934.1.S1_at | TNFSF11 | 1.54±0.18 |
| Cfa.6673.1.A1_at | ZCCHC11 | -1.51±0.17 |
| **Oxidation-reduction process** | | |
| CfaAffx.17336.1.S1_s_at | ACACB | 1.65±0.25 |
| Cfa.14365.1.A1_s_at | ACADSB | -1.50±0.14 |
| CfaAffx.12337.1.S1_s_at | ACSS2 | -1.58±0.32 |
| Cfa.6896.1.A1_at | ADCY2 | 1.94±0.31 |
| Cfa.9585.1.A1_at | ADHFE1 | -1.50±0.33 |
| Cfa.19197.2.S1_at | ALDH9A1 | -1.72±0.38 |
| Cfa.10558.2.S1_s_at | CREG1 | 1.5±0.02 |
| CfaAffx.12221.1.S1_s_at | CYP26A1 | -2.58±0.34 |
| CfaAffx.8512.1.S1_s_at | CYP2B6 | -1.7±0.24 |
| Cfa.17639.1.S1_s_at | DCXR | 2.6±1.01 |
| CfaAffx.16305.1.S1_at | DHCR7 | -1.96±0.15 |
| CfaAffx.1080.1.S1_at | KDSR | -1.53±0.06 |
| CfaAffx.28433.1.S1_at | LEPR | -1.53±0.23 |
| CfaAffx.5541.1.S1_at | MTHFD2L | -1.51±0.08 |
| CfaAffx.11880.1.S1_at | PDHB | -1.5±0.22 |
| CfaAffx.25935.1.S1_at | PKLR | -1.78±0.46 |
| Cfa.19835.1.S1_s_at | PRKAR1B | 1.52±0.16 |
| Cfa.13912.1.A1_at | RRM2 | 1.95±0.7 |
| CfaAffx.24345.1.S1_at | SCCPDH | 1.52±0.24 |
| CfaAffx.13821.1.S1_s_at | TET3 | -1.51±0.22 |
| Cfa.15702.1.S1_at | TXNDC5 | 1.52±0.32 |
| **Cell cycle** | | |
| Cfa.15097.1.A1_at | ANAPC1 | 1.42±0.23 |
| Cfa.20663.1.A1_at | CDK1 | 1.72±0.24 |
| CfaAffx.12680.1.S1_s_at | CLIP1 | 1.64±0.41 |
| CfaAffx.15385.1.S1_s_at | CYLD | 1.73±0.46 |
| CfaAffx.23135.1.S1_s_at | DLGAP5 | 1.56±0.15 |
| Cfa.9662.1.A1_at | DSN1 | 1.53±0.29 |
| CfaAffx.15979.1.S1_at | E2F3 | 1.44±0.1 |
| CfaAffx.25835.1.S1_s_at | FBXW11 | 1.48±0.1 |
| Cfa.15293.1.A1_at | KIF23 | 1.46±0.3 |
| CfaAffx.1540.1.S1_at | LATS1 | 1.81±0.17 |
| CfaAffx.4364.1.S1_s_at | MELK | 1.45±0.29 |
| Cfa.1476.1.A1_at | NCAPG | 1.51±0.36 |
| Cfa.3066.1.S1_at | NDC80 | 1.47±0.26 |
| Cfa.20194.1.S1_s_at | NUF2 | 1.41±0.23 |
| Cfa.1521.1.S1_at | NUSAP1 | 1.52±0.32 |
| CfaAffx.6214.1.S1_at | PSMA2 | -1.56±0.31 |
| Cfa.638.1.S1_at | PSMC6 | -1.55±0.56 |
| Cfa.13912.1.A1_at | RRM2 | 1.95±0.7 |
| CfaAffx.18433.1.S1_at | SPC25 | 1.48±0.25 |
| CfaAffx.26861.1.S1_at | TEX14 | -1.55±0.36 |
| Cfa.18946.1.S1_s_at | TOP2A | 2.11±0.78 |
| CfaAffx.17765.1.S1_s_at | TXNIP | -1.58±0.27 |
| **Defense response** | | |
| Cfa.6037.1.S1_s_at | A2M | 1.79±0.3 |
| Cfa.6896.1.A1_at | ADCY2 | 1.94±0.31 |
| CfaAffx.15843.1.S1_at | BMPR1B | -1.51±0.34 |
| Cfa.4552.1.S1_s_at | CAMP | 2.55±0.86 |
| Cfa.20663.1.A1_at | CDK1 | 1.72±0.24 |
| Cfa.14495.1.S1_at | CFI | -1.6±0.48 |
| CfaAffx.19298.1.S1_at | CTSG | 1.54±0.25 |
| CfaAffx.15385.1.S1_s_at | CYLD | 1.73±0.46 |
| Cfa.14486.1.S1_s_at | HAMP | -4.99±1.35 |
| CfaAffx.3889.1.S1_s_at | HSP90AB1 | 1.71±0.41 |
| Cfa.3511.1.S1_at | IL1B | 1.82±0.32 |
| CfaAffx.14090.1.S1_at | LBP | 3.21±1.64 |
| CfaAffx.25943.1.S1_at | LCP2 | 1.51±0.31 |
| CfaAffx.21286.1.S1_s_at | LTF | 1.51±0.06 |
| CfaAffx.28061.1.S1_s_at | MAP2K7 | 1.50±0.23 |
| Cfa.21191.1.S1_at | OAS1A | -1.51±0.19 |
| Cfa.19835.1.S1_s_at | PRKAR1B | 1.52±0.16 |
| CfaAffx.26852.1.S1_at | S100A8 | 1.57±0.1 |
| Cfa.3173.2.A1_x_at | SAA1 | 3.09±1.02 |
| CfaAffx.24148.1.S1_s_at | SFTPD | 1.5±0.2 |
| Cfa.21204.1.S1_at | THBS1 | 1.74±0.48 |
| CfaAffx.17765.1.S1_s_at | TXNIP | -1.58±0.27 |
| CfaAffx.1247.1.S1_s_at | VNN1 | 1.66±0.08 |
| CfaAffx.17336.1.S1_s_at | ACACB | 1.65±0.25 |
| Cfa.14365.1.A1_s_at | ACADSB | -1.49±0.14 |
| Cfa.9802.1.S1_at | ACSL6 | 1.66±0.21 |
| CfaAffx.12337.1.S1_s_at | ACSS2 | -1.58±0.32 |
| Cfa.16041.1.S1_at | ANKRD1 | 1.42±0.24 |
| CfaAffx.15843.1.S1_at | BMPR1B | -1.43±0.34 |
| CfaAffx.12221.1.S1_s_at | CYP26A1 | -2.58±0.34 |
| CfaAffx.25636.1.S1_at | DGAT2L6 | 1.42±0.32 |
| CfaAffx.16305.1.S1_at | DHCR7 | -1.96±0.15 |
| Cfa.10678.1.A1_at | ELOVL2 | 4.01±1.67 |
| CfaAffx.11994.1.S1_at | FABP1 | 4.66±2.72 |
| Cfa.18651.1.S1_s_at | FDPS | -1.5±0.24 |
| CfaAffx.28804.1.S1_s_at | GK | 1.61±0.33 |
| CfaAffx.21204.1.S1_s_at | GPC3 | 1.58±0.36 |
| Cfa.11107.1.A1_at | IDI1 | -1.59±0.24 |
| Cfa.3511.1.S1_at | IL1B | 1.82±0.32 |
| CfaAffx.17374.1.S1_s_at | INPP5A | -1.43±0.31 |
| CfaAffx.1080.1.S1_at | KDSR | -1.53±0.06 |
| CfaAffx.28433.1.S1_at | LEPR | -1.44±0.23 |
| CfaAffx.20531.1.S1_s_at | MBTPS2 | 1.46±0.25 |
| Cfa.2757.1.S1_at | MOGAT1 | -1.55±0.24 |
| Cfa.16830.1.S1_at | OXCT1 | -1.83±1.4 |
| CfaAffx.15010.1.S1_at | PLIN2 | -1.56±0.37 |
| CfaAffx.12266.1.S1_s_at | PNPLA2 | 1.48±0.29 |
| Cfa.3173.2.A1_x_at | RBP2 | 3.09±1.02 |
| Cfa.15673.1.S1_at | SAA1 | 1.88±0.5 |
| CfaAffx.3314.1.S1_at | SMPDL3A | 1.65±0.26 |
|  |  |  |
| **High dose_Liver** | | |
| **Probe set ID** | **Gene symbol** | **Fold change (average)±SD** |
| **Immune response** | | |
| CfaAffx.25331.1.S1_at | AQP9 | -3.15±1.6 |
| Cfa.6259.1.A1_at | ARPC1A | 2.1±0.29 |
| Cfa.13405.1.A1_at | C9 | 2.78±1.05 |
| Cfa.16612.1.S2_s_at | CAPZA2 | 2.02±0.53 |
| CfaAffx.6642.1.S1_s_at | CD164 | 2.59±1.16 |
| Cfa.21507.1.S1_at | CD46 | 2.49±1.19 |
| CfaAffx.11979.1.S1_at | CEBPG | 2.61±1.27 |
| Cfa.8242.1.A1_s_at | CFI | 3.05±0.86 |
| Cfa.21450.1.S1_s_at | CTSB | 2.45±0.34 |
| Cfa.20779.1.S1_at | CXCL12 | -2.4±1.2 |
| CfaAffx.15385.1.S1_s_at | CYLD | 2.12±0.9 |
| Cfa.14548.1.S1_at | DAK | 2.96±1.62 |
| Cfa.3884.1.S1_at | DDOST | 2.31±0.81 |
| Cfa.15172.1.A1_at | DOCK1 | 2.1±0.88 |
| Cfa.1016.1.A1_s_at | ENPP2 | -2.88±1.65 |
| Cfa.173.1.A1_s_at | FCGR1 | 3.54±2.04 |
| CfaAffx.30397.1.S1_at | FCNB | 5.25±2.42 |
| Cfa.14486.1.S1_s_at | HAMP | -4.12±1.98 |
| CfaAffx.6326.1.S1_s_at | HC | 2.71±1.03 |
| CfaAffx.15349.1.S1_at | HERC6 | -2.36±0.67 |
| Cfa.16440.1.S1_at | IFI35 | -3.67±1.54 |
| Cfa.3380.1.S1_at | IFI44L | -2.65±1.46 |
| Cfa.12195.14.S1_s_at | IGKC | 2.34±0.74 |
| Cfa.2867.1.A1_at | IK | 2.05±0.81 |
| Cfa.3511.1.S1_at | IL1B | 2.02±0.16 |
| CfaAffx.4117.1.S1_at | IL1R1 | 8.23±1.92 |
| Cfa.5221.1.A1_s_at | IL1R2 | 4.96±2.1 |
| Cfa.16944.1.S1_at | ITPR3 | 2.17±0.67 |
| CfaAffx.14090.1.S1_at | LBP | 32.93±2.07 |
| CfaAffx.17218.1.S1_at | LGMN | 2.81±1.17 |
| Cfa.15713.1.A1_s_at | MARCO | 2.61±0.95 |
| CfaAffx.24527.1.S1_s_at | NCF1 | 3.95±2.06 |
| Cfa.21191.1.S1_a_at | NEDD4 | -2.75±1.41 |
| CfaAffx.21142.1.S1_s_at | OAS1A | 5.32±2.23 |
| Cfa.12527.1.A1_at | PRG4 | 2.59±0.99 |
| CfaAffx.26852.1.S1_at | PRKCSH | 9.77±2.02 |
| Cfa.3173.2.A1_at | S100A8 | 61.77±3.97 |
| CfaAffx.24148.1.S1_s_at | SAA1 | 3.74±0.97 |
| CfaAffx.22578.1.S1_at | SFTPD | 2.64±1.1 |
| CfaAffx.13822.1.S1_s_at | SLC11A1 | 2.14±0.55 |
| Cfa.118.1.S1_at | THBS1 | 2.11±0.74 |
| Cfa.20893.1.S1_at | TLR4 | -2.69±3.03 |
| CfaAffx.471.1.S1_s_at | TNFSF10 | 2.34±0.84 |
| CfaAffx.19102.1.S1_at | TUBB4B | 2.1±0.69 |
| CfaAffx.30317.1.S1_at | UBE2D1 | -2.01±0.6 |
| CfaAffx.25438.1.S1_at | VAV2 | 2.01±0.39 |
| Cfa.6442.1.A1_at | VSIG4 | 1.89±0.69 |
| **Inflammatory response** | | |
| Cfa.18689.1.S1_at | A2M | 5.78±1.41 |
| Cfa.3891.1.S1_x_at | APOC3 | -3.55±1.03 |
| CfaAffx.3670.1.S1_at | B4GALT1 | 3.46±1.93 |
| Cfa.4396.1.A1_at | CAMK1D | 2.38±0.96 |
| Cfa.19174.1.S1_s_at | CD163 | 2.3±0.89 |
| CfaAffx.13394.1.S1_s_at | EPHX2 | -2.43±1.06 |
| Cfa.173.1.A1_s_at | FCGR1 | 3.54±2.04 |
| Cfa.13273.1.A1_x_at | FGG | 2.3±0.51 |
| CfaAffx.6326.1.S1_s_at | HC | 2.71±1.03 |
| Cfa.3511.1.S1_at | IL1B | 2.02±0.16 |
| Cfa.15539.1.A1_s_at | KLKB1 | -2.07±1.6 |
| CfaAffx.21050.1.S1_s_at | KNG1 | -2.48±1.84 |
| CfaAffx.14090.1.S1_at | LBP | 32.93±2.07 |
| Cfa.5195.1.A1_s_at | LXN | 4.56±2.39 |
| Cfa.7827.1.A1_at | MGLL | -3.05±1.32 |
| Cfa.15049.1.S1_s_at | NMI | 2.87±1.21 |
| Cfa.61.2.S1_s_at | PTGS1/ COX-1 | -1.85±0.75 |
| Cfa.3449.1.S1_s_at | PTGS2/ COX-2 | 5.34±2.54 |
| CfaAffx.26852.1.S1_at | S100A8 | 9.77±2.02 |
| Cfa.3173.2.A1_at | SAA1 | 61.77±3.97 |
| CfaAffx.29354.1.S1_s_at | SERPINF2 | 2.26±0.76 |
| CfaAffx.22578.1.S1_at | SLC11A1 | 2.64±1.1 |
| Cfa.18951.1.S1_at | TFRC | 6.33±1.49 |
| CfaAffx.13822.1.S1_s_at | THBS1 | 2.14±0.55 |
| Cfa.118.1.S1_at | TLR4 | 2.11±0.74 |
| **Stress response** | | |
| Cfa.18689.1.S1_at | A2M | 5.78±1.41 |
| Cfa.18.2.S1_at | ABCC2 | -2.57±1.95 |
| CfaAffx.18000.1.S1_at | ABHD2 | 2.05±0.46 |
| CfaAffx.31225.1.S1_at | ACADM | -2.03±0.76 |
| CfaAffx.22504.1.S1_s_at | ACAT1 | 2.05±0.44 |
| Cfa.20983.1.S1_at | ADAM9 | 3.18±1.55 |
| CfaAffx.20142.1.S1_s_at | ADH5 | -2.03±0.75 |
| Cfa.17192.1.S1_at | ALAD | -2.39±2.72 |
| CfaAffx.3596.1.S1_s_at | ALDH1A1 | -4.84±6.12 |
| CfaAffx.26135.1.S1_s_at | ALDOA | 2.37±1.02 |
| CfaAffx.13980.1.S1_s_at | ANXA3 | -2.47±0.75 |
| Cfa.3891.1.S1_x_at | APOC3 | -3.55±1.03 |
| Cfa.16211.1.S1_at | APOE | 3.6±0.69 |
| CfaAffx.25331.1.S1_at | AQP9 | -3.15±1.6 |
| Cfa.9384.1.S1_s_at | ARG2 | 2.18±0.48 |
| Cfa.6259.1.A1_at | ARPC1A | 2.1±0.29 |
| Cfa.13202.1.S1_s_at | ASL | -2.9±1.78 |
| Cfa.12008.1.A1_at | ASNS | 4.63±1.48 |
| CfaAffx.3670.1.S1_at | B4GALT1 | 3.46±1.93 |
| CfaAffx.19936.1.S1_s_at | BCCIP | 2±0.54 |
| Cfa.13686.1.A1_s_at | BCL2L11 | 2.33±0.84 |
| Cfa.2953.1.A1_at | BTG3 | 4.17±1.94 |
| Cfa.13405.1.A1_at | C9 | 2.78±1.05 |
| Cfa.4396.1.A1_at | CAMK1D | 2.38±0.96 |
| Cfa.16612.1.S2_s_at | CAPZA2 | 2.02±0.53 |
| Cfa.20942.1.S1_s_at | CA3 | -24.38±1.86 |
| Cfa.4563.1.A1_at | CCS | -2.36±1.01 |
| Cfa.19174.1.S1_s_at | CD163 | 2.3±0.89 |
| Cfa.21507.1.S1_at | CD46 | 2.49±1.19 |
| Cfa.3854.1.S1_at | CD9 | 3.2±1.44 |
| Cfa.11563.1.A1_at | CDKN1A | 5.1±1.17 |
| CfaAffx.11979.1.S1_at | CEBPG | 2.61±1.27 |
| Cfa.8242.1.A1_s_at | CFI | 3.05±0.86 |
| CfaAffx.7939.1.S1_at | CGREF1 | 4.2±1.77 |
| Cfa.14626.1.S1_at | COL3A1 | -4.29±1.61 |
| CfaAffx.22010.1.S1_s_at | COMT | -3.07±1.57 |
| Cfa.21450.1.S1_s_at | CTSB | 2.45±0.34 |
| CfaAffx.13148.1.S1_s_at | CXADR | 2.24±0.59 |
| Cfa.20779.1.S1_at | CXCL12 | -2.4±1.2 |
| CfaAffx.15385.1.S1_s_at | CYLD | 2.12±0.9 |
| CfaAffx.27467.1.S1_s_at | CYP1A2 | -3.93±1.54 |
| Cfa.14548.1.S1_at | DAK | -2.96±1.62 |
| Cfa.3762.1.A1_s_at | DCN | -2.11±0.78 |
| CfaAffx.572.1.S1_at | DDIT3 | 3.04±1.21 |
| Cfa.3884.1.S1_at | DDOST | 2.31±0.81 |
| CfaAffx.22084.1.S1_s_at | DNAJC10 | 2.15±0.89 |
| Cfa.16320.1.A1_s_at | DNAJC3 | 4.49±2.5 |
| Cfa.15172.1.A1_at | DOCK1 | 2.1±0.88 |
| Cfa.15116.1.S1_s_at | DPP4 | 3±1.55 |
| Cfa.11054.1.A1_at | DPYSL3 | -2.24±1.23 |
| CfaAffx.4509.1.S1_s_at | DST | -2.26±1.41 |
| CfaAffx.21064.1.S1_at | DUSP10 | -2.58±1.28 |
| CfaAffx.2782.1.S1_s_at | EGR1 | 6.6±3 |
| CfaAffx.13394.1.S1_s_at | EPHX2 | -2.43±1.06 |
| CfaAffx.1262.1.S1_s_at | EYA4 | 2.31±1.03 |
| Cfa.173.1.A1_s_at | FCGR1 | 3.54±2.04 |
| CfaAffx.30397.1.S1_at | FCNB | 5.25±2.42 |
| Cfa.13273.1.A1_x_at | FGG | 2.3±0.51 |
| CfaAffx.7459.1.S1_at | FOXA3 | 2.15±0.73 |
| Cfa.2376.1.A1_at | GCLM | -2.24±0.39 |
| CfaAffx.20753.1.S1_s_at | GOSR2 | 2.04±0.75 |
| Cfa.878.1.A1_s_at | GSTM5 | -9.55±1.75 |
| Cfa.14486.1.S1_s_at | HAMP | -4.12±1.98 |
| CfaAffx.9805.1.S1_at | HAO1 | -3.19±1.61 |
| CfaAffx.6326.1.S1_s_at | HC | 2.71±1.03 |
| CfaAffx.15349.1.S1_at | HERC6 | -2.36±0.67 |
| Cfa.808.1.S1_at | HERPUD1 | 2.27±0.92 |
| Cfa.14368.1.A1_at | HMGCS2 | -2.68±6.31 |
| CfaAffx.8715.1.S1_s_at | HNMT | -2.09±1.79 |
| Cfa.13172.1.S1_at | HSD3B2 | -7.6±3.8 |
| Cfa.10153.1.S1_at | HSPA8 | -8.24±1.57 |
| Cfa.11015.1.A1_at | HUS1 | 2.24±0.58 |
| CfaAffx.18907.1.S1_at | HYOU1 | 3.18±1.58 |
| Cfa.278.2.S1_a_at | IDH2 | 2.21±0.67 |
| Cfa.3380.1.S1_at | IFI44L | -2.65±1.46 |
| CfaAffx.19068.1.S1_at | IGFBP1 | 5.97±3.56 |
| Cfa.12195.14.S1_s_at | IGKC | 2.34±0.74 |
| Cfa.3136.1.A1_s_at | IL17RB | -2.41±1.34 |
| Cfa.3511.1.S1_at | IL1B | 2.02±0.16 |
| CfaAffx.4117.1.S1_at | IL1R1 | 8.23±4.92 |
| Cfa.16944.1.S1_at | ITPR3 | 2.17±0.67 |
| CfaAffx.2994.1.S1_s_at | KDELR3 | 2.09±0.81 |
| Cfa.15539.1.A1_s_at | KLKB1 | -2.07±1.6 |
| CfaAffx.21050.1.S1_s_at | KNG1 | -2.48±1.84 |
| CfaAffx.11668.1.S1_s_at | KRT8 | 3.3±1.6 |
| CfaAffx.14090.1.S1_at | LBP | 32.93±2.07 |
| CfaAffx.17218.1.S1_at | LGMN | 2.81±1.17 |
| CfaAffx.25334.1.S1_at | LIPC | -4.49±1.84 |
| Cfa.5195.1.A1_s_at | LXN | 4.56±2.39 |
| Cfa.16144.1.S1_at | MACROD1 | -2.42±2.23 |
| CfaAffx.25362.1.S1_at | MAD2L2 | 2.15±0.29 |
| Cfa.15713.1.A1_s_at | MARCO | 2.61±0.95 |
| Cfa.7827.1.A1_at | MGLL | -3.05±1.32 |
| Cfa.18659.1.S1_at | MGST1 | -2.94±1.59 |
| Cfa.18658.1.S1_a_at | MSRA | -2.23±1.27 |
| Cfa.12184.1.A1_at | NDRG1 | 4.27±2.57 |
| CfaAffx.24527.1.S1_s_at | NEDD4 | 3.95±2.06 |
| Cfa.3096.1.A1_at | NEIL3 | -3.59±0.9 |
| Cfa.15049.1.S1_s_at | NMI | 2.87±1.21 |
| CfaAffx.31023.1.S1_at | NQO1 | -2.77±1.98 |
| Cfa.21191.1.S1_a_at | OAS1A | -2.75±1.41 |
| Cfa.16830.1.S1_at | OXCT1 | -6.24±3.46 |
| Cfa.5563.1.A1_s_at | PDIA6 | 2.24±0.9 |
| Cfa.12195.6.A1_at | PDLIM1 | 2.01±0.78 |
| CfaAffx.15569.1.S1_s_at | PEBP1 | -2.81±2.52 |
| CfaAffx.25228.1.S1_at | PFKFB1 | -2.61±4.09 |
| CfaAffx.25935.1.S1_at | PKLR | -8.22±1.5 |
| Cfa.9738.1.S1_s_at | PRKAA1 | 3.39±1.35 |
| Cfa.12527.1.A1_at | PRKCSH | 2.59±0.99 |
| Cfa.882.1.A1_at | PSMA1 | -2.18±0.73 |
| Cfa.14371.1.S1_s_at | PSMB9 | -2.03±0.15 |
| Cfa.61.2.S1_s_at | PTGS1/ COX-1 | -1.85±0.75 |
| Cfa.3449.1.S1_s_at | PTGS2/ COX-2 | 5.34±2.54 |
| Cfa.16941.1.S1_at | PYCR1 | 2.13±0.28 |
| Cfa.9358.1.A1_at | RGS14 | -2.27±0.83 |
| Cfa.17844.1.A1_at | RNASEL | -2.64±3.32 |
| CfaAffx.26852.1.S1_at | S100A8 | 9.77±2.02 |
| Cfa.3173.2.A1_at | SAA1 | 61.77±3.97 |
| CfaAffx.24902.1.S1_at | SERPINE2 | 2.15±0.84 |
| CfaAffx.29354.1.S1_s_at | SERPINF2 | 2.26±0.76 |
| CfaAffx.24148.1.S1_s_at | SFTPD | 3.74±0.97 |
| CfaAffx.22578.1.S1_at | SLC11A1 | 2.64±1.1 |
| Cfa.75.1.S1_at | SOD1 | -2.46±0.97 |
| Cfa.17154.1.S1_at | SORD | -4.25±1.06 |
| CfaAffx.14997.1.S1_s_at | SSR1 | 3.46±2 |
| CfaAffx.15629.1.S1_s_at | STAT1 | 4.06±2.08 |
| Cfa.70.1.A1_s_at | SULT1A1 | -2.69±1.83 |
| Cfa.18951.1.S1_at | TFRC | 6.33±1.49 |
| CfaAffx.13822.1.S1_s_at | THBS1 | 2.14±0.55 |
| Cfa.118.1.S1_at | TLR4 | 2.11±0.74 |
| CfaAffx.25960.1.S1_s_at | TPM1 | -2.09±1.36 |
| Cfa.12502.1.A1_at | TRIB1 | 3.72±2.05 |
| CfaAffx.13305.1.S1_s_at | TSC22D2 | 2.12±0.8 |
| CfaAffx.471.1.S1_s_at | TUBB4B | 2.34±0.84 |
| CfaAffx.19102.1.S1_at | UBE2D1 | 2.1±0.69 |
| CfaAffx.1869.1.S1_s_at | UBR5 | 2.42±1.01 |
| Cfa.12204.1.A1_at | UPP1 | 7.84±1.94 |
| CfaAffx.30317.1.S1_at | VAV2 | -2.01±0.6 |
| Cfa.21389.1.S1_at | VIMP | 2.62±1.31 |
| CfaAffx.25438.1.S1_at | VSIG4 | 2.01±0.39 |
| Cfa.111.1.A1_s_at | VWF | 4.22±1.71 |
| Cfa.4588.1.A1_at | WIPI1 | 6.13±1.05 |
| CfaAffx.22623.1.S1_s_at | WNT4 | 2.91±1.32 |
| CfaAffx.24413.1.S1_at | WNT5B | -3.31±2.61 |
| Cfa.12341.1.A1_at | YY1 | -2.35±1.44 |
| CfaAffx.9244.1.S1_at | ZFP36 | 2.68±1.35 |
| **Glutathione metabolic process** | | |
| Cfa.2376.1.A1_at | GCLM | -2.24±0.39 |
| CfaAffx.5557.1.S1_at | GGCT | 2±0.51 |
| Cfa.1976.1.A1_at | GLO1 | -2.23±0.6 |
| Cfa.537.1.S1_at | GSTA4 | -5.29±1.8 |
| Cfa.878.1.A1_s_at | GSTM3 | -9.55±1.75 |
| CfaAffx.30320.1.S1_s_at | GSTM4 | -4.77±1.49 |
| CfaAffx.26294.1.S1_s_at | GSTZ1 | -2.51±1.86 |
| CfaAffx.22214.1.S1_s_at | HBD/HBG2 | -4.23±1.82 |
| Cfa.18659.1.S1_at | MGST1 | -2.94±1.59 |
| Cfa.12422.1.A1_at | MGST2 | -5.99±3.31 |
| Cfa.75.1.S1_at | SOD1 | -2.46±0.97 |
| **Oxidation-reduction process** | | |
| Cfa.13983.1.S1_s_at | ABCD3 | 2.29±0.53 |
| CfaAffx.21401.1.S1_at | ACADL | -2.33±1.37 |
| CfaAffx.31225.1.S1_at | ACADM | -2.03±0.76 |
| Cfa.14365.1.A1_s_at | ACADSB | -2.3±1.01 |
| Cfa.20564.1.S1_s_at | ACSS1 | 2.5±1.01 |
| CfaAffx.12337.1.S1_s_at | ACSS2 | -2.46±0.61 |
| CfaAffx.16423.1.S1_s_at | ADH4 | -3.26±1.22 |
| CfaAffx.20142.1.S1_s_at | ADH5 | -2.03±0.75 |
| Cfa.10882.1.A1_at | ADIPOR2 | -2.34±1.89 |
| Cfa.13124.1.A1_at | AKR1D1 | -3.06±1.92 |
| CfaAffx.3596.1.S1_s_at | ALDH1A1 | -4.84±1.12 |
| Cfa.2170.1.S1_at | ALDH1B1 | -5.24±1.26 |
| Cfa.9671.1.A1_at | ALDH1L1 | -2.06±1.52 |
| Cfa.15712.1.A1_at | ALDH4A1 | -2.94±1.74 |
| Cfa.403.1.A1_s_at | ALDH7A1 | -2.44±2.36 |
| Cfa.12575.1.S1_at | ALDH9A1 | -3.73±1.37 |
| Cfa.4210.2.S2_at | ATP5J | -2.06±0.84 |
| Cfa.4563.1.A1_at | CCS | -2.36±1.01 |
| Cfa.4354.1.S1_a_at | CRYL1 | -3.02±0.81 |
| Cfa.17211.1.S1_at | CRYZ | -2.56±2.74 |
| CfaAffx.27467.1.S1_s_at | CYP1A2 | -3.93±1.54 |
| CfaAffx.12221.1.S1_s_at | CYP26A1 | -4.44±0.25 |
| CfaAffx.22992.1.S1_at | CYP27A1 | -2.51±3.82 |
| Cfa.3883.1.S1_at | CYP2B6 | -8.63±1.07 |
| Cfa.10767.1.S1_s_at | CYP3A4 | -2.31±1.3 |
| Cfa.18637.1.S1_x_at | CYP4A11 | -2.54±0.62 |
| CfaAffx.11967.1.S1_at | CYP4V2 | -2.78±2.86 |
| CfaAffx.17496.1.S1_s_at | DAO | -2.25±1.65 |
| CfaAffx.16305.1.S1_at | DHCR7 | -2.08±0.94 |
| Cfa.3648.1.S1_at | DHDH | -3.27±1.76 |
| CfaAffx.14436.1.S1_at | DMGDH | -3.36±1.4 |
| CfaAffx.22084.1.S1_s_at | DNAJC10 | 2.15±0.89 |
| CfaAffx.20460.1.S1_s_at | EHHADH | -2.43±1.34 |
| Cfa.1095.1.A1_at | FAM213A | -2.25±1.66 |
| Cfa.1879.1.S1_s_at | FAR1 | -3.23±1.58 |
| Cfa.204.1.S1_s_at | G6PC | -2.2±1.08 |
| CfaAffx.22564.1.S1_s_at | GAPDH | 2.12±0.41 |
| CfaAffx.12587.1.S1_at | GBE1 | -2.35±0.32 |
| CfaAffx.3022.1.S1_s_at | GLDC | -2.04±0.94 |
| Cfa.247.1.S1_at | GLUD1 | -2.3±1.42 |
| CfaAffx.13165.1.S1_s_at | GPD1 | -5.11±1.9 |
| CfaAffx.4525.1.S1_at | HAAO | -2.03±1.36 |
| Cfa.18694.1.S1_at | HADH | -2.02±0.99 |
| CfaAffx.9805.1.S1_at | HAO1 | -3.19±1.61 |
| CfaAffx.21049.1.S1_at | HHIPL2 | -2.72±1.17 |
| Cfa.2048.1.S1_at | HIBADH | -2.13±1.42 |
| Cfa.441.1.A2_at | HSD17B12 | -2.52±0.93 |
| Cfa.20903.1.S1_s_at | HSD17B13 | -3.08±1.77 |
| CfaAffx.6781.1.S1_s_at | HSD17B14 | -2.61±2.87 |
| CfaAffx.1219.1.S1_s_at | HSD17B4 | 2.05±0.79 |
| CfaAffx.1191.1.S1_at | HSD17B6 | 2.65±0.91 |
| Cfa.13172.1.S1_at | HSD3B2 | -7.6±3.8 |
| Cfa.278.2.S1_a_at | IDH2 | 2.21±0.67 |
| Cfa.4592.1.S1_s_at | IMPDH2 | 2.31±1.03 |
| Cfa.16944.1.S1_at | ITPR3 | 2.17±0.67 |
| Cfa.13139.1.A1_at | IYD | -2.26±1.82 |
| Cfa.14297.1.A1_s_at | MAOB | -2.06±0.76 |
| Cfa.18659.1.S1_at | MGST1 | -2.94±1.59 |
| Cfa.12422.1.A1_at | MGST2 | -5.99±3.31 |
| Cfa.5237.1.A1_at | MLXIPL | -3.39±2.66 |
| CfaAffx.30572.1.S1_at | MLYCD | 2.23±0.98 |
| Cfa.18658.1.S1_a_at | MSRA | -2.23±1.27 |
| Cfa.21627.1.S1_s_at | MTHFD1 | -2.22±0.87 |
| Cfa.9904.1.A1_s_at | NDUFB5 | -2.72±1.81 |
| Cfa.4406.1.S1_at | NDUFC1 | -2.16±0.68 |
| Cfa.10895.1.A1_at | NDUFS1 | -2.26±1.25 |
| CfaAffx.31023.1.S1_at | NQO1 | -2.77±1.98 |
| Cfa.12508.1.A1_at | PCBD1 | -2.54±2.44 |
| Cfa.5563.1.A1_s_at | PDIA6 | 2.24±0.9 |
| CfaAffx.25228.1.S1_at | PFKFB1 | -2.62±1.09 |
| CfaAffx.28543.1.S1_s_at | PGM1 | -2.29±1.18 |
| CfaAffx.25935.1.S1_at | PKLR | -8.22±1.5 |
| Cfa.9738.1.S1_s_at | PRKAA1 | 3.39±1.35 |
| Cfa.61.2.S1_s_at | PTGS1/ COX-1 | -1.85±0.75 |
| Cfa.3449.1.S1_s_at | PTGS2/ COX-2 | 5.34±2.54 |
| Cfa.16941.1.S1_at | PYCR1 | 2.13±0.28 |
| CfaAffx.19707.1.S1_at | QSOX1 | 3.04±1.23 |
| Cfa.12560.1.A1_at | RNLS | -2.36±2.72 |
| CfaAffx.29303.1.S1_s_at | SCP2 | -3.05±3.62 |
| Cfa.13227.1.A1_at | SLC27A2 | -2.68±2.07 |
| Cfa.75.1.S1_at | SOD1 | -2.46±0.97 |
| Cfa.17154.1.S1_at | SORD | -4.25±1.06 |
| Cfa.9430.1.A1_at | STEAP2 | -4.87±2.91 |
| CfaAffx.30226.1.S1_s_at | SURF1 | -2.4±1.04 |
| CfaAffx.3164.1.S1_s_at | TST | 2.34±0.68 |
| Cfa.15702.1.S1_at | TXNDC5 | 4.85±2.4 |
| Cfa.3210.1.A1_at | UGP2 | -3.66±2.88 |
| **Response to cytokine stimulus** | | |
| Cfa.20983.1.S1_at | ADAM9 | 3.18±1.55 |
| Cfa.17192.1.S1_at | ALAD | -2.39±2.72 |
| Cfa.16211.1.S1_at | APOE | 3.6±0.69 |
| Cfa.9384.1.S1_s_at | ARG2 | 2.18±0.48 |
| Cfa.13202.1.S1_s_at | ASL | 2.9±2.78 |
| Cfa.14626.1.S1_at | COL3A1 | -4.29±1.61 |
| Cfa.11261.1.A1_at | COMMD7 | -2.12±0.76 |
| Cfa.21450.1.S1_s_at | CTSB | 2.45±0.34 |
| Cfa.20779.1.S1_at | CXCL12 | -2.4±1.2 |
| Cfa.3884.1.S1_at | DDOST | 2.31±0.81 |
| Cfa.11054.1.A1_at | DPYSL3 | -2.24±1.23 |
| CfaAffx.2782.1.S1_s_at | EGR1 | 6.6±3 |
| CfaAffx.28402.1.S1_s_at | GHR | -2.06±0.97 |
| CfaAffx.13165.1.S1_s_at | GPD1 | -5.11±1.9 |
| CfaAffx.8715.1.S1_s_at | HNMT | -2.09±1.79 |
| Cfa.16440.1.S1_at | IFI35 | -3.67±1.54 |
| CfaAffx.14354.1.S1_at | IFNGR2 | 2.23±0.93 |
| CfaAffx.28101.1.S1_s_at | IL13RA1 | 2.87±1.18 |
| Cfa.3136.1.A1_s_at | IL17RB | -2.41±1.34 |
| Cfa.3511.1.S1_at | IL1B | 2.02±0.16 |
| CfaAffx.4117.1.S1_at | IL1R1 | 8.23±1.92 |
| CfaAffx.17252.1.S1_at | IL3RA | 2.06±0.78 |
| Cfa.4592.1.S1_s_at | IMPDH2 | 2.31±1.03 |
| Cfa.13782.1.A1_at | KPNB1 | -2.01±0.46 |
| CfaAffx.438.1.S1_s_at | KRT18 | 3.01±1.33 |
| CfaAffx.11668.1.S1_s_at | KRT8 | 3.3±1.6 |
| Cfa.34.1.S1_s_at | MCL1 | 2.75±1.24 |
| Cfa.16272.1.S1_at | MYLK3 | -3.13±0.4 |
| CfaAffx.24527.1.S1_s_at | NEDD4 | 3.95±2.06 |
| Cfa.3449.1.S1_s_at | PTGS2 | 5.34±2.54 |
| Cfa.17844.1.A1_at | RNASEL | -2.64±3.32 |
| Cfa.18653.1.S1_s_at | SERPINA3 | 5.9±3.39 |
| CfaAffx.22578.1.S1_at | SLC11A1 | 2.64±1.1 |
| CfaAffx.15629.1.S1_s_at | STAT1 | 4.06±2.08 |
| Cfa.9266.1.A1_s_at | SYNCRIP | 2.01±0.62 |
| CfaAffx.13822.1.S1_s_at | THBS1 | 2.14±0.55 |
| CfaAffx.24933.1.S1_at | USP18 | -2.03±1 |
| **Regulation of cell death** | | |
| CfaAffx.18955.1.S1_s_at | ACTA1 | -2.26±0.25 |
| CfaAffx.3596.1.S1_s_at | ALDH1A1 | -4.84±6.12 |
| Cfa.16211.1.S1_at | APOE | 3.6±0.69 |
| CfaAffx.12759.1.S1_at | APPL1 | 2.07±0.72 |
| Cfa.12008.1.A1_at | ASNS | 4.63±2.48 |
| Cfa.11989.1.A1_at | ATF5 | 2.02±0.45 |
| CfaAffx.3670.1.S1_at | B4GALT1 | 3.46±1.93 |
| Cfa.13280.2.S1_s_at | BCAR1 | 2.11±0.77 |
| Cfa.13686.1.A1_s_at | BCL2L11 | 2.33±0.84 |
| Cfa.16935.1.S1_at | BNIP1 | 2.06±0.62 |
| Cfa.13405.1.A1_at | C9 | 2.78±1.05 |
| Cfa.4396.1.A1_at | CAMK1D | 2.38±0.96 |
| Cfa.11563.1.A1_at | CDKN1A | 5.1±1.17 |
| Cfa.18114.1.S1_s_at | CFLAR | 3.17±1.56 |
| CfaAffx.16624.1.S1_s_at | CSTB | 4.1±1.89 |
| Cfa.20779.1.S1_at | CXCL12 | -2.4±1.2 |
| CfaAffx.15385.1.S1_s_at | CYLD | 2.12±0.9 |
| CfaAffx.572.1.S1_at | DDIT3 | 3.04±1.21 |
| CfaAffx.2242.1.S1_at | DEPTOR | -3.49±3.89 |
| CfaAffx.2782.1.S1_s_at | EGR1 | 6.6±3 |
| CfaAffx.12220.1.S1_at | FAIM | -2.02±0.54 |
| Cfa.10648.1.A1_at | GABRB3 | -2.04±1.52 |
| Cfa.2376.1.A1_at | GCLM | -2.24±0.39 |
| CfaAffx.28402.1.S1_s_at | GHR | -2.06±0.97 |
| Cfa.1976.1.A1_at | GLO1 | -2.23±0.6 |
| Cfa.7437.1.A1_at | GLS2 | -2.93±2.55 |
| CfaAffx.6326.1.S1_s_at | C5 | 2.71±1.03 |
| CfaAffx.10256.1.S1_at | HDAC3 | 2.6±0.94 |
| Cfa.808.1.S1_at | HERPUD1 | 2.27±0.92 |
| CfaAffx.10518.1.S1_s_at | HGF | 2.63±1.31 |
| Cfa.3888.1.S1_at | IGF1 | -4.25±2.08 |
| Cfa.3511.1.S1_at | IL1B | 2.02±0.16 |
| CfaAffx.21050.1.S1_s_at | KNG1 | -2.48±1.84 |
| CfaAffx.438.1.S1_s_at | KRT18 | 3.01±1.33 |
| CfaAffx.17218.1.S1_at | LGMN | 2.81±1.17 |
| Cfa.34.1.S1_s_at | MCL1 | 2.75±1.24 |
| CfaAffx.31023.1.S1_at | NQO1 | -2.77±1.98 |
| CfaAffx.26232.1.S1_s_at | OGT | 2.1±0.74 |
| CfaAffx.20252.1.S1_s_at | PAK2 | 2.01±0.69 |
| Cfa.17832.1.S1_at | PDXK | 2.08±0.77 |
| CfaAffx.11365.1.S1_s_at | PLK2 | 3.23±1.58 |
| Cfa.9738.1.S1_s_at | PRKAA1 | 3.39±1.35 |
| Cfa.882.1.A1_at | PSMA1 | -2.18±0.73 |
| Cfa.14371.1.S1_s_at | PSMB9 | -2.03±0.15 |
| Cfa.3449.1.S1_s_at | PTGS2 | 5.34±2.54 |
| CfaAffx.26852.1.S1_at | S100A8 | 9.77±2.02 |
| Cfa.18653.1.S1_s_at | SERPINA3 | 5.9±3.39 |
| Cfa.1370.1.A1_at | SLC2A3 | 2.27±0.65 |
| Cfa.75.1.S1_at | SOD1 | -2.46±0.97 |
| Cfa.1378.1.A1_at | SPHK2 | -2.11±0.14 |
| CfaAffx.15629.1.S1_s_at | STAT1 | 4.06±2.08 |
| CfaAffx.13822.1.S1_s_at | THBS1 | 2.14±0.55 |
| Cfa.118.1.S1_at | TLR4 | 2.11±0.74 |
| Cfa.20893.1.S1_at | TNFSF10 | -2.69±3.03 |
| Cfa.15702.1.S1_at | TXNDC5 | 4.85±2.4 |
| Cfa.21389.1.S1_at | VIMP | 2.62±1.31 |
| CfaAffx.22623.1.S1_s_at | WNT4 | 2.91±1.32 |
| **Lipid metabolism** | | |
| Cfa.19.2.A1_at | ABCB4 | 2.15±0.73 |
| Cfa.13983.1.S1_s_at | ABCD3 | 2.29±0.53 |
| CfaAffx.4636.1.S1_at | ABCG5 | -2.58±1.54 |
| CfaAffx.21401.1.S1_at | ACADL | -2.33±1.37 |
| CfaAffx.31225.1.S1_at | ACADM | -2.03±0.76 |
| Cfa.14365.1.A1_s_at | ACADSB | -2.3±1.01 |
| CfaAffx.22504.1.S1_s_at | ACAT1 | 2.05±0.44 |
| CfaAffx.13750.1.S1_at | ACOT12 | -3.15±4 |
| Cfa.11382.1.A1_at | ACSF2 | -3.45±4.27 |
| Cfa.14057.1.A1_at | ACSL3 | 2.06±0.06 |
| CfaAffx.27505.1.S1_s_at | ACSM3 | -2.72±1.44 |
| CfaAffx.27507.1.S1_s_at | ACSM4 | -2.9±6.54 |
| CfaAffx.27545.1.S1_at | ACSM5 | -3.15±0.65 |
| Cfa.20564.1.S1_s_at | ACSS1 | 2.5±1.01 |
| CfaAffx.12337.1.S1_s_at | ACSS2 | -2.46±0.61 |
| CfaAffx.16423.1.S1_s_at | ADH4 | -3.26±8.22 |
| CfaAffx.20142.1.S1_s_at | ADH5 | -2.03±0.75 |
| Cfa.10882.1.A1_at | ADIPOR2 | -2.34±1.89 |
| CfaAffx.3596.1.S1_s_at | ALDH1A1 | -4.84±6.12 |
| Cfa.3891.1.S1_x_at | APOC3 | -3.55±1.03 |
| Cfa.14460.1.A1_x_at | APOC4 | -2.73±4.72 |
| Cfa.16211.1.S1_at | APOE | 3.6±0.69 |
| Cfa.18524.1.S1_at | ARV1 | -2.16±1.31 |
| Cfa.20832.1.S1_at | CDS1 | -5.96±5.87 |
| CfaAffx.22010.1.S1_s_at | CEL | -3.07±1.57 |
| Cfa.2596.1.S1_at | COMT | -2.38±1.52 |
| Cfa.4354.1.S1_a_at | CRLS1 | -3.02±0.81 |
| CfaAffx.27467.1.S1_s_at | CRYL1 | -3.93±15.54 |
| CfaAffx.12221.1.S1_s_at | CYP1A2 | -4.44±0.25 |
| CfaAffx.22992.1.S1_at | CYP26A1 | -2.51±3.82 |
| CfaAffx.11967.1.S1_at | CYP27A1 | -2.78±2.86 |
| Cfa.4341.1.S1_at | CYP4V3 | -2.15±0.81 |
| CfaAffx.16305.1.S1_at | DBI | -2.08±0.94 |
| CfaAffx.20460.1.S1_s_at | DHCR7 | -2.43±1.34 |
| Cfa.10678.1.A1_at | EHHADH | 9.75±3.04 |
| Cfa.1016.1.A1_s_at | ELOVL2 | -2.88±4.65 |
| CfaAffx.13394.1.S1_s_at | ENPP2 | -2.43±1.06 |
| CfaAffx.7415.1.S1_at | EPHX2 | 2.88±1.34 |
| Cfa.1879.1.S1_s_at | EPT1 | 3.23±1.58 |
| Cfa.204.1.S1_s_at | ERLIN1 | -2.2±1.08 |
| Cfa.15493.1.A1_s_at | FABP7 | -3.09±3.6 |
| Cfa.10965.1.A1_at | FAR1 | -1.83±1.40 |
| CfaAffx.28402.1.S1_s_at | FGF21 | -2.06±0.97 |
| CfaAffx.13165.1.S1_s_at | G6PC | -5.11±1.9 |
| CfaAffx.21204.1.S1_s_at | GC | 3.23±1.08 |
| Cfa.18694.1.S1_at | GHR | -2.02±0.99 |
| CfaAffx.9805.1.S1_at | GLUD1 | -3.19±7.61 |
| CfaAffx.10256.1.S1_at | GPD1 | 2.6±0.94 |
| Cfa.14368.1.A1_at | GPT | -2.68±6.31 |
| Cfa.441.1.A2_at | GYK | -2.52±0.93 |
| CfaAffx.6781.1.S1_s_at | HADH | -2.61±2.87 |
| CfaAffx.1219.1.S1_s_at | HAO1 | 2.05±0.79 |
| CfaAffx.1191.1.S1_at | HDAC3 | 2.65±0.91 |
| Cfa.13172.1.S1_at | HMGCS2 | -7.6±3.8 |
| Cfa.11107.1.A1_at | HSD17B12 | -2.08±0.49 |
| Cfa.3511.1.S1_at | HSD17B14 | 2.02±0.16 |
| Cfa.900.1.A1_at | HSD17B4 | 2.02±0.58 |
| CfaAffx.17218.1.S1_at | HSD17B6 | 2.81±1.17 |
| CfaAffx.25334.1.S1_at | HSD3B2 | -4.49±6.84 |
| Cfa.10658.1.A1_at | IDI1 | -2.7±3.2 |
| Cfa.7827.1.A1_at | IL1B | -3.05±4.32 |
| Cfa.12422.1.A1_at | IMPAD1 | -5.99±3.31 |
| CfaAffx.30572.1.S1_at | KRT18 | 2.23±0.98 |
| CfaAffx.16508.1.S1_at | KRT8 | 2.07±0.8 |
| Cfa.2307.1.S1_at | LGMN | 2.06±0.64 |
| Cfa.16909.1.S1_s_at | LIPC | -3.89±2.28 |
| Cfa.360.1.A1_at | LRAT | -2.44±1.29 |
| Cfa.16830.1.S1_at | MGLL | -6.24±3.46 |
| CfaAffx.25193.1.S1_at | MGST2 | 2.66±1.04 |
| Cfa.4926.1.A1_s_at | MLXIPL | 2.87±1.49 |
| Cfa.14542.1.A1_s_at | MLYCD | -2.32±1.71 |
| Cfa.9738.1.S1_s_at | MTTP | 3.39±1.35 |
| Cfa.3449.1.S1_s_at | MYO5A | 5.34±2.54 |
| Cfa.3173.2.A1_at | NANS | 61.77±3.97 |
| CfaAffx.29303.1.S1_s_at | NCEH1 | -3.05±3.62 |
| Cfa.18809.2.S1_s_at | OXCT1 | -2.5±0.96 |
| CfaAffx.27000.1.S1_at | PI4K2B | -2.95±2.38 |
| Cfa.13227.1.A1_at | PLA2G16 | -2.68±2.07 |
| CfaAffx.2004.1.S1_s_at | PLIN2 | -2.31±1.25 |
| Cfa.6227.1.A1_at | PON1 | -2.84±0.92 |
| Cfa.1378.1.A1_at | PRKAA1 | -2.11±0.14 |
| Cfa.590.1.S1_at | PTGS2 | 2.37±0.74 |
| CfaAffx.21321.1.S1_at | SAA1 | -2.33±1.05 |
| Cfa.70.1.A1_s_at | SCP2 | -2.69±2.83 |
| Cfa.3501.1.S1_at | SCPEP1 | -2.35±0.75 |
| Cfa.118.1.S1_at | SERPINA6 | 2.11±0.74 |
| Cfa.6459.1.A1_s_at | SLC27A2 | -3.79±13.61 |
| CfaAffx.22623.1.S1_s_at | SLC27A6 | 2.91±1.32 |
| Cfa.5237.1.A1_at | SOAT2 | -3.39±2.17 |
| CfaAffx.2395.1.S1_s_at | SPHK2 | 2.34±0.98 |
| CfaAffx.15056.1.S1_at | SPTSSA | 2.25±0.78 |
| CfaAffx.3314.1.S1_at | STARD5 | 8.11±4.19 |
| CfaAffx.6792.1.S1_at | SULT1A1 | 3.87±1.56 |
| CfaAffx.7249.1.S1_s_at | SULT1B1 | -2.3±1 |
| Cfa.20911.1.S1_s_at | TLR4 | -2.2±0.59 |
| CfaAffx.11659.1.S1_at | TTR | 2.52±0.06 |
| Cfa.7839.1.S1_x_at | WNT4 | 3.09±1.18 |
| **Defense response** | | |
| Cfa.18689.1.S1_at | A2M | 5.78±1.41 |
| CfaAffx.13980.1.S1_s_at | ANXA3 | -2.47±0.75 |
| Cfa.3891.1.S1_x_at | APOC3 | 3.55±1.03 |
| Cfa.6259.1.A1_at | ARPC1A | 2.1±0.29 |
| CfaAffx.3670.1.S1_at | B4GALT1 | 3.46±1.93 |
| Cfa.13405.1.A1_at | C9 | 2.78±1.05 |
| Cfa.4396.1.A1_at | CAMK1D | 2.38±0.96 |
| Cfa.16612.1.S2_s_at | CAPZA2 | 2.02±0.53 |
| Cfa.19174.1.S1_s_at | CD163 | 2.3±0.89 |
| Cfa.21507.1.S1_at | CD46 | 2.49±1.19 |
| CfaAffx.11979.1.S1_at | CEBPG | 2.61±1.27 |
| Cfa.8242.1.A1_s_at | CFI | 3.05±0.86 |
| Cfa.21450.1.S1_s_at | CTSB | 2.45±0.34 |
| CfaAffx.13148.1.S1_s_at | CXADR | 2.24±0.59 |
| CfaAffx.15385.1.S1_s_at | CYLD | 2.12±0.9 |
| Cfa.14548.1.S1_at | DAK | -2.96±1.62 |
| Cfa.3884.1.S1_at | DDOST | 2.31±0.81 |
| Cfa.16320.1.A1_s_at | DNAJC3 | 4.49±2.5 |
| Cfa.15172.1.A1_at | DOCK1 | 2.1±0.88 |
| CfaAffx.13394.1.S1_s_at | EPHX2 | -2.43±1.06 |
| Cfa.173.1.A1_s_at | FCGR1A (IgG) | 3.54±2.04 |
| CfaAffx.30397.1.S1_at | FCN1 | 5.25±2.42 |
| Cfa.13273.1.A1_x_at | FGG | 2.3±0.51 |
| Cfa.14486.1.S1_s_at | HAMP | -4.12±1.98 |
| CfaAffx.6326.1.S1_s_at | C5 | 2.71±1.03 |
| CfaAffx.15349.1.S1_at | HERC6 | -2.36±0.67 |
| Cfa.3380.1.S1_at | IFI44L | -2.65±4.46 |
| Cfa.12195.14.S1_s_at | IGKC | 2.34±0.74 |
| Cfa.3136.1.A1_s_at | IL17RB | -2.41±1.34 |
| Cfa.3511.1.S1_at | IL1B | 2.02±0.16 |
| Cfa.16944.1.S1_at | ITPR3 | 2.17±0.67 |
| Cfa.15539.1.A1_s_at | KLKB1 | -2.07±1.6 |
| CfaAffx.21050.1.S1_s_at | KNG1 | -2.48±1.84 |
| CfaAffx.14090.1.S1_at | LBP | 32.93±2.07 |
| CfaAffx.17218.1.S1_at | LGMN | 2.81±1.17 |
| Cfa.5195.1.A1_s_at | LXN | 4.56±2.39 |
| Cfa.15713.1.A1_s_at | MARCO | 2.61±0.95 |
| Cfa.7827.1.A1_at | MGLL | -3.05±4.32 |
| Cfa.15049.1.S1_s_at | NMI | 2.87±1.21 |
| Cfa.21191.1.S1_a_at | OAS1A | -2.75±1.41 |
| Cfa.12527.1.A1_at | PRKCSH | 2.59±0.99 |
| Cfa.3449.1.S1_s_at | PTGS2 | 5.34±2.54 |
| Cfa.17844.1.A1_at | RNASEL | -2.64±3.32 |
| CfaAffx.26852.1.S1_at | S100A8 | 9.77±2.02 |
| Cfa.3173.2.A1_at | SAA1 | 61.77±3.97 |
| Cfa.18653.1.S1_s_at | SERPINA3 | 5.9±3.39 |
| CfaAffx.29354.1.S1_s_at | SERPINF2 | 2.26±0.76 |
| CfaAffx.24148.1.S1_s_at | SFTPD | 3.74±0.97 |
| CfaAffx.22578.1.S1_at | SLC11A1 | 2.64±1.1 |
| CfaAffx.15629.1.S1_s_at | STAT1 | 4.06±2.08 |
| Cfa.18951.1.S1_at | TFRC | 6.33±1.49 |
| CfaAffx.13822.1.S1_s_at | THBS1 | 2.14±0.55 |
| Cfa.118.1.S1_at | TLR4 | 2.11±0.74 |
| CfaAffx.471.1.S1_s_at | TUBB4B | 2.34±0.84 |
| CfaAffx.19102.1.S1_at | UBE2D1 | 2.1±0.69 |
| CfaAffx.30317.1.S1_at | VAV2 | -2.01±0.6 |
| CfaAffx.25438.1.S1_at | VSIG4 | 2.01±0.39 |
| **Cell cycle** | | |
| Cfa.11563.1.A1_at | CDKN1A | 5.1±1.17 |
| Cfa.3888.1.S1_at | IGF1 | -4.25±2.08 |
| Cfa.3511.1.S1_at | IL1B | 2.02±0.16 |
| Cfa.12184.1.A1_at | NDRG1 | 4.27±2.57 |
| CfaAffx.15569.1.S1_s_at | PEBP1 | -2.81±2.52 |
| Cfa.3365.1.A1_at | PKP4 | -2.64±1.74 |
| Cfa.882.1.A1_at | PSMA1 | -2.18±0.73 |
| Cfa.14371.1.S1_s_at | PSMB9 | -2.03±0.15 |
| CfaAffx.22623.1.S1_s_at | WNT4 | 2.91±1.32 |
| **Response to wounding** | | |
| Cfa.18689.1.S1_at | A2M | 5.78±1.41 |
| CfaAffx.18000.1.S1_at | ABHD2 | 2.05±0.46 |
| Cfa.3891.1.S1_x_at | APOC3 | -3.55±1.03 |
| Cfa.16211.1.S1_at | APOE | 3.6±0.69 |
| Cfa.9384.1.S1_s_at | ARG2 | 2.18±0.48 |
| CfaAffx.3670.1.S1_at | B4GALT1 | 3.46±1.93 |
| Cfa.4396.1.A1_at | CAMK1D | 2.38±0.96 |
| Cfa.19174.1.S1_s_at | CD163 | 2.3±0.89 |
| Cfa.14626.1.S1_at | COL3A1 | -4.29±1.61 |
| Cfa.21450.1.S1_s_at | CTSB | 2.45±0.34 |
| Cfa.3762.1.A1_s_at | DCN | -2.11±0.78 |
| Cfa.11054.1.A1_at | DPYSL3 | -2.24±1.23 |
| CfaAffx.4509.1.S1_s_at | DST | -2.26±2.41 |
| CfaAffx.13394.1.S1_s_at | EPHX2 | -2.43±1.06 |
| Cfa.173.1.A1_s_at | FCGR1 | 3.54±2.04 |
| Cfa.13273.1.A1_x_at | FGG | 2.3±0.51 |
| CfaAffx.6326.1.S1_s_at | C5 | 2.71±1.03 |
| Cfa.3511.1.S1_at | IL1B | 2.02±0.16 |
| Cfa.15539.1.A1_s_at | KLKB1 | -2.07±1.6 |
| CfaAffx.21050.1.S1_s_at | KNG1 | -2.48±1.84 |
| CfaAffx.14090.1.S1_at | LBP | 32.93±2.07 |
| Cfa.5195.1.A1_s_at | LXN | 4.56±2.39 |
| Cfa.7827.1.A1_at | MGLL | -3.05±1.32 |
| Cfa.15049.1.S1_s_at | NMI | 2.87±1.21 |
| CfaAffx.15569.1.S1_s_at | PEBP1 | -2.81±2.52 |
| Cfa.3449.1.S1_s_at | PTGS2 | 5.34±2.54 |
| CfaAffx.26852.1.S1_at | S100A8 | 9.77±7.02 |
| Cfa.3173.2.A1_at | SAA1 | 61.77±3.97 |
| Cfa.18653.1.S1_s_at | SERPINA3 | 5.9±3.39 |
| CfaAffx.24902.1.S1_at | SERPINE2 | 2.15±0.84 |
| CfaAffx.29354.1.S1_s_at | SERPINF2 | 2.26±0.76 |
| CfaAffx.22578.1.S1_at | SLC11A1 | 2.64±1.1 |
| Cfa.75.1.S1_at | SOD1 | -2.46±0.97 |
| Cfa.18951.1.S1_at | TFRC | 6.33±1.49 |
| CfaAffx.13822.1.S1_s_at | THBS1 | 2.14±0.55 |
| Cfa.118.1.S1_at | TLR4 | 2.11±0.74 |
| CfaAffx.25960.1.S1_s_at | TPM1 | -2.09±1.36 |
| Cfa.111.1.A1_s_at | VWF | 4.22±1.71 |
| CfaAffx.24413.1.S1_at | WNT5B | -3.31±2.61 |
|  |  |  |
| **Low dose_Kidney** | | |
| **Probe set ID** | **Gene symbol** | **Fold change (average)±SD** |
| **Oxidation-reduction process** | | |
| Cfa.8764.2.A1_at | ACSBG2 | 1.51±0.35 |
| CfaAffx.27833.1.S1_at | ALDH3A2 | 1.51±0.3 |
| Cfa.11091.1.A1_at | ALKBH2 | 1.54±0.21 |
| Cfa.16384.1.A1_at | AMACR | -1.58±0.31 |
| Cfa.15225.1.S1_a_at | CPT2 | 1.51±0.11 |
| CfaAffx.31237.1.S1_at | CRYZ | -1.50±0.37 |
| CfaAffx.14450.1.S1_at | DMGDH | -1.54±0.76 |
| CfaAffx.23030.1.S1_s_at | FMO1 | 2.23±0.76 |
| CfaAffx.3223.1.S1_at | FOXRED2 | 1.52±0.16 |
| CfaAffx.30687.1.S1_at | FRRS1 | 1.54±0.18 |
| Cfa.17006.1.S1_at | GMPR | 1.51±0.14 |
| CfaAffx.15407.1.S1_at | HTATIP2 | -1.54±0.2 |
| Cfa.16221.1.S1_at | LEPR | -1.64±0.2 |
| CfaAffx.12248.1.S1_at | PAM | -1.79±0.18 |
| Cfa.10925.1.S1_at | PHYHIPL | -1.65±0.37 |
| Cfa.1207.1.S1_s_at | SNAP25 | 1.54±0.15 |
| **Regulation of cell cycle** | | |
| CfaAffx.11466.1.S1_at | BUB1 | 1.55±0.19 |
| Cfa.1126.1.S1_at | CASC5 | -1.61±0.31 |
| Cfa.16245.1.S1_at | CCNB3 | 1.51±0.15 |
| Cfa.872.1.A1_at | CCNG1 | -1.59±0.53 |
| Cfa.2173.1.A1_at | CDKN2C | 1.52±0.18 |
| Cfa.844.2.S1_s_at | CEP63 | -1.58±0.48 |
| CfaAffx.11992.1.S1_s_at | CSPP1 | -1.54±0.3 |
| Cfa.86.1.S1_at | FGFR2 | -1.53±0.47 |
| CfaAffx.17535.1.S1_s_at | LEF1 | 1.59±0.22 |
| CfaAffx.15820.1.S1_at | NPM1 | 1.54±0.28 |
| CfaAffx.13793.1.S1_at | RBL1 | -1.54±0.21 |
| Cfa.3187.4.A1_at | RPS27 | -1.52±0.35 |
| CfaAffx.7099.1.S1_at | SESN3 | 1.75±0.1 |
| Cfa.13471.1.A1_at | SIN3B | -1.45±0.13 |
| CfaAffx.12794.1.S1_at | TERF1 | -1.63±1 |
| Cfa.20786.2.S1_s_at | UBE2E1 | -1.52±0.2 |
| **Lipid metabolism** | | |
| CfaAffx.5036.1.S1_at | ABCA1 | -1.52±0.25 |
| Cfa.8764.2.A1_at | ACSBG2 | 1.51±0.35 |
| CfaAffx.27507.1.S1_s_at | ACSM4 | 1.44±0.11 |
| Cfa.67.1.S1_s_at | AFP | -2.23±0.5 |
| CfaAffx.27833.1.S1_at | ALDH3A2 | 1.51±0.3 |
| Cfa.16384.1.A1_at | AMACR | -1.58±0.31 |
| CfaAffx.28713.1.S1_s_at | ANGPTL3 | -1.71±0.84 |
| Cfa.6294.1.A1_at | APOA4 | 1.45±0.06 |
| CfaAffx.15238.1.S1_s_at | CHD9 | 1.75±0.13 |
| Cfa.15225.1.S1_a_at | CPT2 | 1.51±0.11 |
| CfaAffx.12520.1.S1_at | ENPP6 | -2.08±1.59 |
| Cfa.18336.1.S1_s_at | LARGE | -1.42±0.27 |
| Cfa.16221.1.S1_at | LEPR | -1.64±0.2 |
| Cfa.15835.1.S1_at | LPL | -1.59±0.4 |
| Cfa.20804.1.S1_at | NR1H2 | -1.51±0.31 |
| CfaAffx.12248.1.S1_at | PAM | -1.79±0.18 |
| Cfa.10925.1.S1_at | PHYHIPL | -1.65±0.37 |
| CfaAffx.15010.1.S1_at | PNPLA2 | -1.43±0.29 |
| Cfa.6245.1.A1_at | RBP2 | 1.79±0.14 |
| Cfa.13471.1.A1_at | SIN3B | -1.45±0.13 |
| **Transmembrane transport** | | |
| CfaAffx.9103.1.S1_at | ABCC4 | -1.53±0.19 |
| CfaAffx.15156.1.S1_at | ABCG2 | 1.56±0.32 |
| CfaAffx.7346.1.S1_at | ATP6V0A4 | -1.51±0.5 |
| Cfa.15225.1.S1_a_at | CPT2 | 1.48±0.11 |
| Cfa.205.1.A1_at | KCNE2 | -1.63±0.19 |
| Cfa.17449.1.S1_at | NUPL1 | -1.48±0.16 |
| CfaAffx.1885.1.S1_s_at | SLC12A2 | 2.1±0.24 |
| CfaAffx.19471.1.S1_s_at | SLC12A8 | 1.49±0.16 |
| CfaAffx.7221.1.S1_s_at | SLC1A5 | -1.67±0.37 |
| Cfa.15571.1.A1_at | SLC22A1 | -1.62±0.23 |
| CfaAffx.12315.1.S1_at | SLC25A36 | 1.5±0.29 |
| Cfa.3561.1.S1_at | SLC3A1 | 1.47±0.03 |
| Cfa.3.1.S1_s_at | SLC46A2 | 1.54±0.18 |
| CfaAffx.15913.1.S1_at | SLC5A12 | -1.44±0.06 |
| Cfa.14178.1.S1_at | SLC5A6 | 1.58±0.29 |
| CfaAffx.11304.1.S1_at | SLC5A8 | -1.86±0.22 |
| Cfa.13649.1.A1_s_at | SLC6A18 | -1.44±0.35 |
| CfaAffx.15479.1.S1_at | SLC6A5 | 1.6±0.13 |
| CfaAffx.21248.1.S1_at | UNC80 | 1.48±0.11 |
|  |  |  |
| **High dose_Kidney** | | |
| **Probe set ID** | **Gene symbol** | **Fold change (average)±SD** |
| **Immune response** | | |
| CfaAffx.19151.1.S1_s_at | ADCY4 | 3.5±1.35 |
| Cfa.18376.1.S1_at | BIRC3 | 3.06±1.24 |
| Cfa.16857.1.S1_at | C1QB | 1.95±0.69 |
| Cfa.10921.1.S1_at | C1QC | 2.77±0.96 |
| Cfa.4589.1.A1_s_at | C1RA | 8.45±1.77 |
| Cfa.10821.1.A1_s_at | C1S | 7.33±2.16 |
| Cfa.12240.1.A1_at | C3 | 6.12±2.01 |
| Cfa.3834.1.S1_at | C5AR1 | 2.2±0.53 |
| Cfa.15481.1.A1_at | C8G | -2.01±0.81 |
| CfaAffx.16422.1.S1_s_at | CCL20 | 2.54±1.13 |
| Cfa.21507.1.S1_at | CD46 | 2.54±0.98 |
| Cfa.1333.3.S1_a_at | CD74 | 5.34±0.97 |
| Cfa.3596.2.S1_at | CD80 | 2.02±0.66 |
| Cfa.18476.1.S1_at | CD83 | -2.15±1.44 |
| Cfa.5944.1.A1_at | CFB | 5.56±2.08 |
| CfaAffx.17824.1.S1_s_at | CFI | 2.15±0.61 |
| CfaAffx.30912.1.S1_at | CHST4 | 3.43±1.94 |
| Cfa.1254.1.S1_s_at | CLU | 9.45±1.5 |
| Cfa.21450.1.S1_s_at | CTSB | 3.75±2.03 |
| CfaAffx.2498.1.S1_s_at | CXCL14 | 3±1.46 |
| CfaAffx.15385.1.S1_s_at | CYLD | 2.01±0.41 |
| Cfa.3524.1.S1_s_at | EGF | -3.06±0.59 |
| Cfa.173.1.A1_s_at | FCGR1 | 2.72±0.94 |
| Cfa.14872.1.A1_at | FGF9 | -2.15±0.83 |
| Cfa.9039.1.A1_at | FOS | 6.51±2.09 |
| Cfa.2958.1.A1_at | GEM | 2.17±0.76 |
| CfaAffx.2197.1.S1_s_at | HLA-DRA | 9.83±1.49 |
| CfaAffx.15349.1.S1_at | HERC6 | 2.06±0.24 |
| CfaAffx.27629.1.S1_s_at | HSP90AA1 | 2.09±0.25 |
| Cfa.4778.1.A1_at | IL10RB | 4.58±1.24 |
| CfaAffx.30861.1.S1_at | IL34 | 4.89±1.16 |
| CfaAffx.4021.1.S1_s_at | KIT | -1.95±0.55 |
| CfaAffx.15108.1.S1_at | LY75 | 3.86±1.03 |
| Cfa.12441.1.S1_s_at | NFKBIA | 2.1±0.81 |
| CfaAffx.6867.1.S1_at | PRKAR2B | -1.92±0.95 |
| Cfa.12527.1.A1_at | PRKCSH | 3.52±1.12 |
| CfaAffx.31132.1.S1_at | PSMB10 | 3.21±0.47 |
| Cfa.4923.1.A1_s_at | RAC1 | 2.27±0.71 |
| CfaAffx.12561.1.S1_s_at | SERPING1 | 2.03±0.59 |
| CfaAffx.4346.1.S1_s_at | TINAG | -1.87±0.18 |
| Cfa.3348.1.A1_at | UBA7 | 2.07±0.63 |
| **Inflammatory response** | | |
| Cfa.3891.1.S1_at | APOC3 | -2.05±1.95 |
| Cfa.12240.1.A1_at | C3 | 6.12±2.01 |
| Cfa.3834.1.S1_at | C5AR1 | 2.2±0.53 |
| CfaAffx.16422.1.S1_s_at | CCL20 | 2.54±1.13 |
| CfaAffx.30912.1.S1_at | CHST4 | 3.43±1.94 |
| CfaAffx.16581.1.S1_at | ELF3 | 2.91±1.23 |
| Cfa.173.1.A1_s_at | FCGR1 | 2.72±0.94 |
| Cfa.9039.1.A1_at | FOS | 6.51±2.09 |
| Cfa.4778.1.A1_at | IL10RB | 4.58±1.24 |
| CfaAffx.30861.1.S1_at | IL34 | 4.89±1.16 |
| CfaAffx.4021.1.S1_s_at | KIT | -1.95±0.55 |
| CfaAffx.15108.1.S1_at | CD302 | 3.86±1.03 |
| CfaAffx.7482.1.S1_s_at | NOX4 | -3.75±1.38 |
| Cfa.4923.1.A1_s_at | RAC1 | 2.27±0.71 |
| CfaAffx.15042.1.S1_s_at | SPP1 | 5.28±0.93 |
| Cfa.20307.1.S1_at | STAT3 | 2.25±0.99 |
| Cfa.18951.1.S1_at | TFRC | 2.86±0.57 |
| CfaAffx.23380.1.S1_s_at | TNFRSF1A | 2.53±0.89 |
| Cfa.1941.1.S1_at | VCAM1 | 9.24±2.05 |
| **Stress response** | | |
| Cfa.436.3.S1_a_at | ABHD2 | 1.92±0.51 |
| CfaAffx.2141.1.S1_s_at | ACSL6 | -1.95±0.44 |
| CfaAffx.19151.1.S1_s_at | ADCY4 | 3.5±1.35 |
| Cfa.2383.1.S1_s_at | ALAS1 | 2.22±0.75 |
| CfaAffx.27784.1.S1_s_at | ALDH3A1 | -2.48±0.74 |
| CfaAffx.21464.1.S1_at | APOBEC1 | 2.15±0.83 |
| Cfa.3891.1.S1_at | APOC3 | -2.05±1.95 |
| Cfa.12876.1.S1_at | AQP1 | -2.26±1.42 |
| Cfa.9384.1.S1_s_at | ARG2 | 3.39±1.44 |
| Cfa.19985.1.S1_s_at | ASNS | 2.27±0.48 |
| Cfa.9097.1.A1_at | ATF3 | 4.69±2.55 |
| Cfa.18370.1.S1_s_at | BDH1 | -2.08±1.59 |
| Cfa.18376.1.S1_at | BIRC3 | 3.06±1.24 |
| CfaAffx.14883.1.S1_at | BTG2 | 2.31±0.65 |
| CfaAffx.13161.1.S1_s_at | BTG3 | 2.32±0.7 |
| Cfa.16857.1.S1_at | C1QB | 1.95±0.69 |
| Cfa.10921.1.S1_at | C1QC | 2.77±0.96 |
| Cfa.4589.1.A1_s_at | C1RA | 8.45±1.77 |
| Cfa.10821.1.A1_s_at | C1S | 7.33±2.16 |
| Cfa.12240.1.A1_at | C3 | 6.12±2.01 |
| Cfa.3834.1.S1_at | C5AR1 | 2.2±0.53 |
| Cfa.15481.1.A1_at | C8G | -2.01±0.81 |
| CfaAffx.16422.1.S1_s_at | CCL20 | 2.54±1.13 |
| Cfa.21507.1.S1_at | CD46 | 2.54±0.98 |
| Cfa.1333.3.S1_a_at | CD74 | 5.34±0.97 |
| Cfa.3596.2.S1_at | CD80 | 2.02±0.66 |
| Cfa.18476.1.S1_at | CD83 | -2.15±1.44 |
| CfaAffx.856.1.S1_x_at | CDC42EP5 | 3.12±1.68 |
| Cfa.11563.1.A1_at | CDKN1A | 2.64±0.36 |
| Cfa.5944.1.A1_at | CFB | 5.56±2.08 |
| CfaAffx.17824.1.S1_s_at | CFI | 2.15±0.61 |
| CfaAffx.18523.1.S1_s_at | CHEK2 | -3.84±3.53 |
| CfaAffx.30912.1.S1_at | CHST4 | 3.43±1.94 |
| Cfa.3513.1.S1_s_at | CLDN3 | 1.87±0.47 |
| Cfa.1254.1.S1_s_at | CLU | 9.45±1.5 |
| CfaAffx.31268.1.S1_s_at | CTH | -2.28±2.11 |
| Cfa.21450.1.S1_s_at | CTSB | 3.75±2.03 |
| CfaAffx.13148.1.S1_at | CXADR | 2.3±0.25 |
| CfaAffx.15385.1.S1_s_at | CYLD | 2.01±0.41 |
| Cfa.8215.1.A1_at | DDIT4 | 2.1±0.73 |
| Cfa.16320.1.A1_s_at | DNAJC3 | 2.03±0.72 |
| Cfa.125.1.S1_s_at | EDN1 | 2.2±0.55 |
| Cfa.3524.1.S1_s_at | EGF | -3.06±0.59 |
| Cfa.11962.1.A1_at | EIF4EBP1 | 2.51±0.63 |
| CfaAffx.16581.1.S1_at | ELF3 | 2.91±1.23 |
| CfaAffx.11994.1.S1_at | FABP1 | -2.61±1.29 |
| CfaAffx.109.1.S1_s_at | FABP5 | 2.66±0.44 |
| CfaAffx.24504.1.S1_x_at | FADS1 | 2.68±0.82 |
| Cfa.7793.1.A1_at | FBXO31 | -2.23±1.36 |
| Cfa.173.1.A1_s_at | FCGR1 | 2.72±0.94 |
| Cfa.14872.1.A1_at | FGF9 | -2.15±0.83 |
| CfaAffx.23030.1.S1_s_at | FMO1 | -2.33±0.54 |
| Cfa.1886.1.S1_at | FNIP2 | -1.9±0.97 |
| Cfa.9039.1.A1_at | FOS | 6.51±2.09 |
| Cfa.12368.1.A1_at | GATA4 | -2.38±0.37 |
| Cfa.18710.1.S1_at | GATM | -3.37±0.76 |
| Cfa.9669.1.S1_at | GPX8 | 2.18±0.54 |
| Cfa.878.1.A1_s_at | GSTM5 | 2.34±0.25 |
| CfaAffx.15349.1.S1_at | HERC6 | 2.06±0.24 |
| Cfa.126.1.S1_s_at | HIF1A | 5.21±2.61 |
| Cfa.13203.1.S1_at | HNMT | -1.98±0.8 |
| CfaAffx.27629.1.S1_s_at | HSP90AA1 | 2.09±0.25 |
| CfaAffx.30936.1.S1_s_at | HSPA5 | 2.68±0.72 |
| Cfa.16863.1.S1_at | IER3 | 4.29±2.37 |
| Cfa.7238.1.A1_at | IGFBP2 | -4.23±1.67 |
| Cfa.4778.1.A1_at | IL10RB | 4.58±1.24 |
| CfaAffx.30861.1.S1_at | IL34 | 4.89±1.16 |
| Cfa.12533.1.A1_at | INSIG1 | -2.17±0.99 |
| CfaAffx.4021.1.S1_s_at | KIT | -1.95±0.55 |
| CfaAffx.8966.1.S1_at | KLF6 | 3.25±1.04 |
| Cfa.7839.1.S1_x_at | KRT8 | 2.62±0.99 |
| Cfa.627.1.S1_s_at | LEAP2 | -1.93±0.88 |
| CfaAffx.15108.1.S1_at | LY75 | 3.86±1.03 |
| CfaAffx.23910.1.S1_at | MAEL | -2.01±0.31 |
| Cfa.3786.1.S1_s_at | MYC | 3.68±1.89 |
| Cfa.10314.1.A1_s_at | NEK6 | 3.28±1.15 |
| Cfa.12441.1.S1_s_at | NFKBIA | 2.1±0.81 |
| CfaAffx.7482.1.S1_s_at | NOX4 | -3.75±1.38 |
| CfaAffx.31023.1.S1_at | NQO1 | -2.37±0.48 |
| Cfa.16830.1.S1_at | OXCT1 | 1.98±0.59 |
| Cfa.21517.1.S1_s_at | PECAM1 | 2.91±1.45 |
| Cfa.1416.1.A1_at | PENK | 3.15±0.53 |
| Cfa.15057.1.A1_at | PON2 | -2.18±0.1 |
| CfaAffx.6867.1.S1_at | PRKAR2B | 1.92±0.95 |
| Cfa.12527.1.A1_at | PRKCSH | 3.52±1.12 |
| CfaAffx.31132.1.S1_at | PSMB10 | 3.21±0.47 |
| Cfa.12298.1.A1_a_at | PSMB8 | 2.05±0.11 |
| Cfa.4923.1.A1_s_at | RAC1 | 2.27±0.71 |
| Cfa.20231.1.A1_s_at | RHOA | 1.9±0.36 |
| Cfa.12674.1.A1_s_at | RYR1 | -2.35±0.23 |
| CfaAffx.12561.1.S1_s_at | SERPING1 | 2.03±0.59 |
| CfaAffx.1885.1.S1_s_at | SLC12A2 | 5.09±1.05 |
| Cfa.1270.1.S1_s_at | SLC1A3 | -2.54±1.05 |
| CfaAffx.15042.1.S1_s_at | SPP1 | 5.28±0.93 |
| Cfa.20307.1.S1_at | STAT3 | 2.25±0.99 |
| Cfa.70.1.A1_s_at | SULT1A1 | -2.33±0.41 |
| Cfa.3155.1.S1_at | TAP1 | 1.91±0.07 |
| Cfa.18951.1.S1_at | TFRC | 2.86±0.57 |
| Cfa.3680.1.S1_s_at | TIMP1 | 12.05±2.55 |
| CfaAffx.23380.1.S1_s_at | TNFRSF1A | 2.53±0.89 |
| CfaAffx.24216.1.S1_at | TPM4 | 2.23±0.35 |
| CfaAffx.15837.1.S1_at | TSPAN32 | -2.01±0.16 |
| Cfa.3348.1.A1_at | UBA7 | 2.07±0.63 |
| CfaAffx.20837.1.S1_at | UCK2 | 2.05±0.41 |
| Cfa.12204.1.A1_at | UPP1 | 6.19±0.49 |
| Cfa.1941.1.S1_at | VCAM1 | 9.24±2.05 |
| Cfa.111.1.A1_s_at | VWF | 1.91±0.31 |
| Cfa.3546.2.S1_s_at | YBX3 | 2.03±0.79 |
| **Oxidation-reduction process** | | |
| CfaAffx.17336.1.S1_s_at | ACACB | -3.82±1.85 |
| Cfa.16751.1.S1_at | ACSS1 | 2.12±0.88 |
| CfaAffx.19151.1.S1_s_at | ADCY4 | 3.5±1.35 |
| CfaAffx.4490.1.S1_at | AGMO | -2.38±1.09 |
| Cfa.6471.1.A1_at | AKR1E1 | -2.6±2.99 |
| CfaAffx.4463.1.S1_at | ALDH1B1 | -2.09±1.19 |
| CfaAffx.27784.1.S1_s_at | ALDH3A1 | 2.48±0.74 |
| CfaAffx.15922.1.S1_at | BBOX1 | 1.94±0.72 |
| Cfa.18370.1.S1_s_at | BDH1 | -2.08±1.59 |
| Cfa.1286.1.A1_at | CPT1A | 2.22±0.9 |
| Cfa.10558.2.S1_at | CREG1 | -1.88±0.24 |
| Cfa.4354.1.S1_a_at | CRYL1 | -2.1±1.81 |
| CfaAffx.11088.1.S1_at | CYB5R2 | 2.27±0.6 |
| CfaAffx.11967.1.S1_at | CYP4V3 | -2.43±1.55 |
| Cfa.21109.1.S1_s_at | DCXR | -1.87±0.62 |
| CfaAffx.14436.1.S1_at | DMGDH | -2.32±0.68 |
| CfaAffx.30709.1.S1_s_at | DPYD | 3.37±1.78 |
| Cfa.299.1.A1_s_at | FABP3 | -3.71±0.89 |
| CfaAffx.24504.1.S1_x_at | FADS1 | 2.68±0.82 |
| CfaAffx.24504.1.S1_s_at | FADS3 | 2.42±0.69 |
| Cfa.17136.1.S1_at | FASN | -4.87±2.22 |
| CfaAffx.23030.1.S1_s_at | FMO1 | -2.33±0.54 |
| Cfa.14546.1.A1_s_at | FMO5 | -2.15±0.51 |
| Cfa.204.1.S1_s_at | G6PC | -2.86±1.15 |
| Cfa.12126.1.A1_at | GCDH | -2.14±1.13 |
| Cfa.17724.1.S1_at | GPD1 | -1.92±0.38 |
| Cfa.9669.1.S1_at | GPX8 | 2.18±0.54 |
| CfaAffx.16567.1.S1_s_at | GSTO1 | 2.35±0.37 |
| Cfa.15749.1.A1_at | H6PD | 1.91±0.56 |
| Cfa.3045.1.A1_s_at | HACL1 | -2.01±1.09 |
| CfaAffx.15742.1.S1_s_at | HAO2 | -2.05±0.4 |
| Cfa.14563.1.A1_s_at | HGD | -2.4±0.53 |
| Cfa.10248.1.S1_at | HSD11B1 | -3.29±2.73 |
| CfaAffx.6781.1.S1_s_at | HSD17B14 | -2±1.09 |
| CfaAffx.30577.1.S1_at | HSD17B2 | -2.15±0.75 |
| CfaAffx.1324.1.S1_at | MTFR2 | 1.89±0.11 |
| Cfa.3786.1.S1_s_at | MYC | 3.68±1.89 |
| CfaAffx.7482.1.S1_s_at | NOX4 | -3.75±1.38 |
| CfaAffx.31023.1.S1_at | NQO1 | -2.37±0.48 |
| Cfa.17166.1.S1_at | PCK2 | -2.06±0.62 |
| CfaAffx.30650.1.S1_s_at | PHYHD1 | -2.35±0.23 |
| CfaAffx.6867.1.S1_at | PRKAR2B | -1.92±0.95 |
| Cfa.11943.1.A1_at | RDH16 | 5.52±2.66 |
| Cfa.13227.1.A1_at | SLC27A2 | -3.56±2.52 |
| CfaAffx.3716.1.S1_at | STEAP1 | 3.03±1.13 |
| Cfa.14500.1.S1_at | TST | 1.88±0.66 |
| **Response to cytokine stimulus** | | |
| CfaAffx.12394.1.S1_s_at | ACSL1 | -4.23±2.01 |
| CfaAffx.27683.1.S1_s_at | ACSL4 | 6.25±2.38 |
| Cfa.9384.1.S1_s_at | ARG2 | 3.39±1.44 |
| Cfa.1333.3.S1_a_at | CD74 | 5.34±0.97 |
| Cfa.21450.1.S1_s_at | CTSB | 3.75±2.03 |
| CfaAffx.24352.1.S1_at | CXCL16 | 12.25±2.83 |
| Cfa.125.1.S1_s_at | EDN1 | 2.2±0.55 |
| Cfa.11431.1.A1_at | EIF4E2 | -1.87±0.23 |
| Cfa.17136.1.S1_at | FASN | -4.87±2.22 |
| Cfa.173.1.A1_s_at | FCGR1A | 2.72±0.94 |
| Cfa.9039.1.A1_at | FOS | 6.51±2.09 |
| Cfa.17724.1.S1_at | GPD1 | -1.92±0.38 |
| Cfa.126.1.S1_s_at | HIF1A | 5.21±2.61 |
| CfaAffx.2197.1.S1_s_at | H2-DMA | 9.83±2.49 |
| Cfa.13203.1.S1_at | HNMT | -1.98±0.80 |
| CfaAffx.30936.1.S1_s_at | HSPA5 | 2.68±0.72 |
| Cfa.10009.1.A1_at | IFNAR2 | 2.37±0.48 |
| Cfa.4778.1.A1_at | IL10RB | 4.58±1.24 |
| CfaAffx.28101.1.S1_s_at | IL13RA1 | 2.73±0.81 |
| CfaAffx.29270.1.S1_at | INPP5K | 2.14±0.7 |
| CfaAffx.4021.1.S1_s_at | KIT | -1.95±0.55 |
| CfaAffx.8966.1.S1_at | KLF6 | 3.25±1.04 |
| CfaAffx.11632.1.S1_s_at | KRT18 | 2.68±0.69 |
| Cfa.7839.1.S1_x_at | KRT8 | 2.62±0.99 |
| CfaAffx.9145.1.S1_at | KYNU | -2.75±1.81 |
| Cfa.34.1.S1_s_at | MCL1 | 2.03±0.53 |
| Cfa.13894.1.S1_s_at | MME | -3.22±0.74 |
| Cfa.3786.1.S1_s_at | MYC | 3.68±1.89 |
| Cfa.2080.1.S1_at | OSMR | 13.47±3.46 |
| Cfa.12298.1.A1_a_at | PSMB8 | 2.05±0.11 |
| Cfa.20307.1.S1_at | STAT3 | 2.25±0.99 |
| Cfa.3680.1.S1_s_at | TIMP1 | 12.05±2.55 |
| CfaAffx.23380.1.S1_s_at | TNFRSF1A | 2.53±0.89 |
| Cfa.3348.1.A1_at | UBA7 | 2.07±0.63 |
| Cfa.1941.1.S1_at | VCAM1 | 9.24±2.05 |
| Cfa.3546.2.S1_s_at | YBX3 | 2.03±0.79 |
| **Regulation of cell cycle** | | |
| CfaAffx.13729.1.S1_at | CCNG2 | -1.92±1.4 |
| Cfa.11563.1.A1_at | CDKN1A | 2.64±0.36 |
| CfaAffx.18523.1.S1_s_at | CHEK2 | -3.84±3.53 |
| Cfa.125.1.S1_s_at | EDN1 | 2.2±0.55 |
| Cfa.3524.1.S1_s_at | EGF | -3.06±0.59 |
| Cfa.7793.1.A1_at | FBXO31 | -2.23±1.36 |
| Cfa.16863.1.S1_at | IER3 | 4.29±2.37 |
| CfaAffx.12575.1.S1_at | KNTC1 | -2.18±0.56 |
| Cfa.4343.1.S1_s_at | MCM5 | -2.93±3.26 |
| Cfa.10314.1.A1_s_at | NEK6 | 3.28±1.15 |
| Cfa.16807.1.S1_at | ORC5 | -1.98±0.57 |
| CfaAffx.11365.1.S1_at | PLK2 | -2.17±1.34 |
| CfaAffx.31132.1.S1_at | PSMB10 | 3.21±0.47 |
| Cfa.12298.1.A1_a_at | PSMB8 | 2.05±0.11 |
| Cfa.20231.1.A1_s_at | RHOA | 1.9±0.36 |
| Cfa.13418.1.A1_at | WEE2 | -2.17±0.51 |
| **Cell death** | | |
| Cfa.9384.1.S1_s_at | ARG2 | 3.39±1.44 |
| Cfa.12514.1.A1_at | BAG3 | 2.13±0.54 |
| Cfa.21056.1.S1_at | BCL2A1A | 2.36±0.95 |
| Cfa.18376.1.S1_at | BIRC3 | 3.06±1.24 |
| Cfa.3834.1.S1_at | C5AR1 | 2.2±0.53 |
| Cfa.15481.1.A1_at | C8G | -2.01±0.81 |
| CfaAffx.18523.1.S1_s_at | CHEK2 | -3.84±3.53 |
| Cfa.1254.1.S1_s_at | CLU | 9.45±1.5 |
| Cfa.8215.1.A1_at | DDIT4 | 2.1±0.62 |
| Cfa.115.1.S1_at | DNASE1 | -3.71±2.34 |
| Cfa.6525.2.A1_s_at | DRAM1 | 1.92±0.25 |
| CfaAffx.18108.1.S1_at | EAF2 | -1.91±1.01 |
| Cfa.5896.1.A1_at | FGGY | -2.18±0.13 |
| CfaAffx.7369.1.S1_s_at | FGL2 | 1.91±0.18 |
| Cfa.18474.1.S1_at | HINT2 | -2.05±0.38 |
| CfaAffx.27629.1.S1_s_at | HSP90AA1 | 2.09±0.25 |
| Cfa.16863.1.S1_at | IER3 | 4.29±2.37 |
| Cfa.15687.1.A1_at | ITSN1 | 2.75±1.25 |
| CfaAffx.4021.1.S1_s_at | KIT | -1.95±0.55 |
| CfaAffx.11632.1.S1_s_at | KRT18 | 2.68±0.69 |
| Cfa.7839.1.S1_x_at | KRT8 | 2.62±0.99 |
| Cfa.6460.1.A1_at | LITAF | 2.02±0.03 |
| Cfa.5699.1.A1_at | MCF2L | -2.14±1.33 |
| Cfa.34.1.S1_s_at | MCL1 | 2.03±0.53 |
| Cfa.3786.1.S1_s_at | MYC | 3.68±1.89 |
| Cfa.10314.1.A1_s_at | NEK6 | 3.28±1.15 |
| Cfa.12441.1.S1_s_at | NFKBIA | 2.1±0.81 |
| CfaAffx.31132.1.S1_at | PSMB10 | 3.21±0.47 |
| Cfa.12298.1.A1_a_at | PSMB8 | 2.05±0.11 |
| Cfa.4923.1.A1_s_at | RAC1 | 2.27±0.71 |
| CfaAffx.11304.1.S1_at | SLC5A8 | -2.5±0.99 |
| CfaAffx.118.1.S1_s_at | TNFRSF12A | 3.02±0.78 |
| CfaAffx.23380.1.S1_s_at | TNFRSF1A | 2.53±0.89 |
| Cfa.4426.1.S1_at | YWHAH | 2.24±0.53 |
| CfaAffx.20922.1.S1_at | ZBTB16 | 3.87±2.04 |
| **Defense response** | | |
| CfaAffx.19151.1.S1_s_at | ADCY4 | 3.5±1.35 |
| CfaAffx.21464.1.S1_at | APOBEC1 | 2.15±0.83 |
| Cfa.3891.1.S1_at | APOC3 | -2.05±1.95 |
| Cfa.18376.1.S1_at | BIRC3 | 3.06±1.24 |
| Cfa.16857.1.S1_at | C1QB | 1.95±0.69 |
| Cfa.10921.1.S1_at | C1QC | 2.77±0.96 |
| Cfa.4589.1.A1_s_at | C1RA | 8.45±1.77 |
| Cfa.10821.1.A1_s_at | C1S | 7.33±2.16 |
| Cfa.12240.1.A1_at | C3 | 6.12±2.01 |
| Cfa.3834.1.S1_at | C5AR1 | 2.2±0.53 |
| Cfa.15481.1.A1_at | C8G | -2.01±0.81 |
| CfaAffx.16422.1.S1_s_at | CCL20 | 2.54±1.13 |
| Cfa.21507.1.S1_at | CD46 | 2.54±0.98 |
| Cfa.1333.3.S1_a_at | CD74 | 5.34±0.97 |
| Cfa.3596.2.S1_at | CD80 | 2.02±0.66 |
| Cfa.18476.1.S1_at | CD83 | -2.15±1.44 |
| Cfa.6458.1.A1_s_at | CFB | 5.03±2.08 |
| CfaAffx.17824.1.S1_s_at | CFI | 2.15±0.61 |
| CfaAffx.30912.1.S1_at | CHST4 | 3.43±1.94 |
| Cfa.1254.1.S1_s_at | CLU | 9.45±1.5 |
| Cfa.21450.1.S1_s_at | CTSB | 3.75±2.03 |
| CfaAffx.13148.1.S1_at | CXADR | 2.3±0.25 |
| CfaAffx.15385.1.S1_s_at | CYLD | 2.01±0.41 |
| Cfa.8215.1.A1_at | DDIT4 | 2.1±0.73 |
| Cfa.16320.1.A1_s_at | DNAJC3 | 2.03±0.72 |
| Cfa.3524.1.S1_s_at | EGF | -3.06±0.59 |
| CfaAffx.16581.1.S1_at | ELF3 | 2.91±1.23 |
| Cfa.173.1.A1_s_at | FCGR1 | 2.72±0.94 |
| Cfa.14872.1.A1_at | FGF9 | -2.15±0.83 |
| Cfa.9039.1.A1_at | FOS | 6.51±2.09 |
| CfaAffx.15349.1.S1_at | HERC6 | 2.06±0.24 |
| CfaAffx.27629.1.S1_s_at | HSP90AA1 | 2.09±0.25 |
| Cfa.4778.1.A1_at | IL10RB | 4.58±1.24 |
| CfaAffx.30861.1.S1_at | IL34 | 4.89±3.16 |
| CfaAffx.4021.1.S1_s_at | KIT | -1.95±0.55 |
| Cfa.627.1.S1_s_at | LEAP2 | -1.93±0.88 |
| CfaAffx.15108.1.S1_at | CD302 | 3.86±1.03 |
| Cfa.12441.1.S1_s_at | NFKBIA | 2.1±0.81 |
| CfaAffx.7482.1.S1_s_at | NOX4 | -3.75±1.38 |
| Cfa.1416.1.A1_at | PENK | 3.15±0.53 |
| CfaAffx.6867.1.S1_at | PRKAR2B | -1.92±0.95 |
| Cfa.12527.1.A1_at | PRKCSH | 3.52±1.12 |
| Cfa.4923.1.A1_s_at | RAC1 | 2.27±0.71 |
| CfaAffx.12561.1.S1_s_at | SERPING1 | 2.03±0.59 |
| CfaAffx.15042.1.S1_s_at | SPP1 | 5.28±0.93 |
| Cfa.20307.1.S1_at | STAT3 | 2.25±0.99 |
| Cfa.3155.1.S1_at | TAP1 | 1.91±0.07 |
| Cfa.18951.1.S1_at | TFRC | 2.86±0.57 |
| CfaAffx.23380.1.S1_s_at | TNFRSF1A | 2.53±0.89 |
| CfaAffx.15837.1.S1_at | TSPAN32 | -2.01±0.16 |
| Cfa.3348.1.A1_at | UBA7 | 2.07±0.63 |
| Cfa.1941.1.S1_at | VCAM1 | 9.24±2.05 |
| **Regulation of cell death** | | |
| CfaAffx.17119.1.S1_s_at | ACSL5 | -2.6±1 |
| Cfa.1387.1.A1_at | AGRN | 2.2±0.75 |
| Cfa.81.1.S1_at | ANXA4 | 2.21±0.41 |
| Cfa.12876.1.S1_at | AQP1 | -2.26±1.42 |
| Cfa.19985.1.S1_s_at | ASNS | 2.27±0.48 |
| Cfa.12514.1.A1_at | BAG3 | 2.13±0.54 |
| Cfa.21056.1.S1_at | BCL2A1A | 2.36±0.95 |
| Cfa.18376.1.S1_at | BIRC3 | 3.06±1.24 |
| CfaAffx.14883.1.S1_at | BTG2 | 2.31±0.65 |
| Cfa.3834.1.S1_at | C5AR1 | 2.2±0.53 |
| Cfa.1333.3.S1_a_at | CD74 | 5.34±0.97 |
| Cfa.11563.1.A1_at | CDKN1A | 2.64±0.36 |
| Cfa.1339.1.A1_at | CHGA | 2.48±0.32 |
| Cfa.1254.1.S1_s_at | CLU | 9.45±1.5 |
| CfaAffx.16624.1.S1_s_at | CSTB | 3.43±0.8 |
| CfaAffx.31268.1.S1_s_at | CTH | -2.28±2.11 |
| CfaAffx.15385.1.S1_s_at | CYLD | 2.01±0.41 |
| Cfa.8215.1.A1_at | DDIT4 | 2.1±0.73 |
| CfaAffx.18108.1.S1_at | EAF2 | -1.91±1.01 |
| Cfa.125.1.S1_s_at | EDN1 | 2.2±0.55 |
| CfaAffx.11994.1.S1_at | FABP1 | -2.61±1.29 |
| Cfa.2400.1.A1_at | FRZB | -2.7±1.89 |
| Cfa.11206.1.A1_at | GABRB3 | -1.96±0.52 |
| Cfa.126.1.S1_s_at | HIF1A | 5.21±2.61 |
| CfaAffx.30936.1.S1_s_at | HSPA5 | 2.68±0.72 |
| Cfa.16863.1.S1_at | IER3 | 4.29±2.37 |
| Cfa.15687.1.A1_at | ITSN1 | 2.75±1.25 |
| CfaAffx.4021.1.S1_s_at | KIT | -1.95±0.55 |
| CfaAffx.11632.1.S1_s_at | KRT18 | 2.68±0.69 |
| CfaAffx.23910.1.S1_at | MAEL | -2.01±0.31 |
| Cfa.34.1.S1_s_at | MCL1 | 2.03±0.53 |
| CfaAffx.28635.1.S1_at | MPV17L | -2.46±1.05 |
| Cfa.15462.1.A1_at | MT1 | 2.12±0.27 |
| Cfa.3786.1.S1_s_at | MYC | 3.68±1.89 |
| Cfa.8884.1.A1_s_at | NDNF | -2.46±1.05 |
| Cfa.12441.1.S1_s_at | NFKBIA | 2.1±0.81 |
| Cfa.15094.1.S1_a_at | NME1 | 2.8±0.69 |
| CfaAffx.7482.1.S1_s_at | NOX4 | -3.75±1.38 |
| CfaAffx.31023.1.S1_at | NQO1 | -2.37±0.48 |
| Cfa.17832.1.S1_at | PDXK | 2.43±0.96 |
| Cfa.2593.1.S1_at | PLAUR | 1.95±0.6 |
| CfaAffx.11365.1.S1_at | PLK2 | -2.17±1.34 |
| CfaAffx.31132.1.S1_at | PSMB10 | 3.21±0.47 |
| Cfa.12298.1.A1_a_at | PSMB8 | 2.05±0.11 |
| Cfa.20231.1.A1_s_at | RHOA | 1.9±0.36 |
| CfaAffx.15042.1.S1_s_at | SPP1 | 5.28±0.93 |
| Cfa.20307.1.S1_at | STAT3 | 2.25±0.99 |
| Cfa.3680.1.S1_s_at | TIMP1 | 12.05±2.55 |
| CfaAffx.118.1.S1_s_at | TNFRSF12A | 3.02±0.78 |
| CfaAffx.23380.1.S1_s_at | TNFRSF1A | 2.53±0.89 |
| CfaAffx.7845.1.S1_s_at | TSC22D1 | 4.18±1.12 |
| Cfa.3546.2.S1_s_at | YBX3 | 2.03±0.79 |
| Cfa.4426.1.S1_at | YWHAH | 2.24±0.53 |
| CfaAffx.20922.1.S1_at | ZBTB16 | 3.87±2.04 |
| **Lipid metabolism** | | |
| CfaAffx.2333.1.S1_at | A4GALT | 2.39±0.81 |
| CfaAffx.3656.1.S1_s_at | ABCB4 | 3.08±1.61 |
| CfaAffx.17336.1.S1_s_at | ACACB | -3.82±1.85 |
| CfaAffx.12394.1.S1_s_at | ACSL1 | -4.23±2.01 |
| CfaAffx.27683.1.S1_s_at | ACSL4 | 6.25±3.38 |
| CfaAffx.17119.1.S1_s_at | ACSL5 | -2.6±1 |
| CfaAffx.2141.1.S1_s_at | ACSL6 | -1.95±0.44 |
| Cfa.12182.1.A1_at | ACSM2 | -3.12±2.25 |
| CfaAffx.27505.1.S1_s_at | ACSM3 | -2.24±1.26 |
| Cfa.16751.1.S1_at | ACSS1 | 2.12±0.88 |
| CfaAffx.4490.1.S1_at | AGMO | -2.38±1.09 |
| Cfa.1387.1.A1_at | AGRN | 2.2±0.75 |
| Cfa.2383.1.S1_s_at | ALAS1 | 2.22±0.75 |
| CfaAffx.20626.1.S1_at | APOA5 | 2.07±0.31 |
| CfaAffx.21464.1.S1_at | APOBEC1 | 2.15±0.83 |
| Cfa.3891.1.S1_at | APOC3 | -2.05±1.95 |
| Cfa.18370.1.S1_s_at | BDH1 | -2.08±1.59 |
| Cfa.1333.3.S1_a_at | CD74 | 5.34±0.97 |
| Cfa.1254.1.S1_s_at | CLIC1 | 9.45±1.5 |
| Cfa.1286.1.A1_at | CLU | 2.22±0.9 |
| Cfa.2596.1.S1_at | CPT1A | -2.38±1.06 |
| Cfa.4354.1.S1_a_at | CRLS1 | -2.1±1.81 |
| CfaAffx.7812.1.S1_s_at | CRYL1 | -2.07±1.29 |
| CfaAffx.11088.1.S1_at | CUBN | 2.27±0.6 |
| CfaAffx.11967.1.S1_at | CYB5R2 | -2.43±1.55 |
| Cfa.125.1.S1_s_at | CYP4V3 | 2.2±0.55 |
| Cfa.9200.1.A1_at | EDN1 | -2.03±1.09 |
| Cfa.6996.1.A1_s_at | ELOVL2 | -2.38±2.43 |
| CfaAffx.11994.1.S1_at | ENPP6 | -2.61±1.29 |
| Cfa.299.1.A1_s_at | FABP1 | -3.71±0.89 |
| CfaAffx.109.1.S1_s_at | FABP3 | 2.66±0.44 |
| CfaAffx.24504.1.S1_x_at | FABP5 | 2.68±0.82 |
| CfaAffx.24504.1.S1_s_at | FADS1 | 2.42±0.69 |
| Cfa.17136.1.S1_at | FADS3 | -4.87±2.22 |
| Cfa.204.1.S1_s_at | FASN | -2.86±1.15 |
| Cfa.12126.1.A1_at | G6PC | -2.14±1.13 |
| Cfa.17724.1.S1_at | GCDH | -1.92±0.38 |
| Cfa.3045.1.A1_s_at | GPD1 | -2.01±1.09 |
| CfaAffx.15742.1.S1_s_at | GPT | -2.05±0.4 |
| Cfa.18474.1.S1_at | HACL1 | -2.05±0.38 |
| Cfa.10248.1.S1_at | HAO2 | -3.29±6.73 |
| CfaAffx.6781.1.S1_s_at | HINT2 | -2±1.09 |
| CfaAffx.30577.1.S1_at | HSD11B1 | -2.15±0.75 |
| Cfa.11107.1.A1_at | HSD17B14 | -4.2±0.23 |
| CfaAffx.29270.1.S1_at | HSD17B2 | 2.14±0.7 |
| Cfa.12533.1.A1_at | IDI1 | -2.17±0.99 |
| CfaAffx.4021.1.S1_s_at | INPP5K | -1.95±0.55 |
| Cfa.10658.1.A1_at | INSIG1 | -7.21±2.82 |
| Cfa.16909.1.S1_s_at | KIT | 2.16±0.74 |
| CfaAffx.13949.1.S1_s_at | KRT18 | 1.93±0.34 |
| Cfa.16830.1.S1_at | KRT8 | -1.98±0.59 |
| Cfa.12578.1.A1_s_at | LAMA1 | 1.89±0.35 |
| CfaAffx.6867.1.S1_at | LRAT | -1.92±0.95 |
| Cfa.6245.1.A1_at | NANS | -8.79±2.36 |
| Cfa.11943.1.A1_at | NR1H3 | 5.52±2.66 |
| CfaAffx.15202.1.S1_s_at | OXCT1 | 2.58±0.97 |
| Cfa.149.1.S1_at | PLCB3 | -5.96±2.74 |
| Cfa.13227.1.A1_at | PLIN2 | -3.56±2.52 |
| Cfa.13388.2.S1_at | PRKAR2B | -2.25±1.15 |
| CfaAffx.27366.1.S1_at | RBP2 | -2.3±0.91 |
| Cfa.70.1.A1_s_at | RDH16 | -2.33±0.41 |
| CfaAffx.5225.1.S1_s_at | SDC4 | -6.53±1.94 |
| Cfa.3680.1.S1_s_at | SLC10A2 | 12.05±2.55 |
| CfaAffx.23380.1.S1_s_at | SLC27A2 | 2.53±0.89 |
| CfaAffx.5454.1.S1_at | SLC44A1 | 3.69±1.48 |
| Cfa.4426.1.S1_at | STRA6 | 2.24±0.53 |
| Cfa.14195.1.A1_s_at | SULT1A1 | -3.68±2.24 |
| CfaAffx.4386.1.S1_s_at | SULT1B1 | 3.11±0.98 |
| CfaAffx.3314.1.S1_at | TIMP1 | 2.26±1.21 |
| Cfa.20911.1.S1_s_at | TNFRSF1A | -2.28±0.06 |
| CfaAffx.11659.1.S1_at | UGCG | 2.68±0.06 |
| Cfa.7839.1.S1_x_at | YWHAH | 2.54±0.81 |
| **Fatty acid metabolic process** | | |
| CfaAffx.17336.1.S1_s_at | ACACB | 3.82±1.85 |
| CfaAffx.12394.1.S1_s_at | ACSL1 | -4.23±2.01 |
| CfaAffx.27683.1.S1_s_at | ACSL4 | 6.25±3.38 |
| CfaAffx.17119.1.S1_s_at | ACSL5 | -2.6±1 |
| CfaAffx.2141.1.S1_s_at | ACSL6 | -1.95±0.44 |
| Cfa.12182.1.A1_at | ACSM2 | -3.12±2.25 |
| CfaAffx.27505.1.S1_s_at | ACSM3 | -2.24±1.26 |
| Cfa.16751.1.S1_at | ACSS1 | 2.12±0.88 |
| CfaAffx.4490.1.S1_at | AGMO | -2.38±1.09 |
| Cfa.12240.1.A1_at | C3 | 6.12±2.01 |
| Cfa.1333.3.S1_a_at | CD74 | 5.34±0.97 |
| Cfa.1286.1.A1_at | CPT1A | 2.22±0.9 |
| Cfa.4354.1.S1_a_at | CRYL1 | -2.1±1.81 |
| CfaAffx.11967.1.S1_at | CYP4V3 | -2.43±1.55 |
| Cfa.125.1.S1_s_at | EDN1 | 2.2±0.55 |
| Cfa.9200.1.A1_at | ELOVL2 | -2.03±1.09 |
| Cfa.299.1.A1_s_at | FABP3 | -3.71±0.89 |
| CfaAffx.24504.1.S1_x_at | FADS1 | 2.68±0.82 |
| CfaAffx.24504.1.S1_s_at | FADS3 | 2.42±0.69 |
| Cfa.17136.1.S1_at | FASN | -4.87±2.22 |
| Cfa.12126.1.A1_at | GCDH | -2.14±1.13 |
| Cfa.3045.1.A1_s_at | HACL1 | -2.01±1.09 |
| CfaAffx.15742.1.S1_s_at | HAO2 | -2.05±0.4 |
| CfaAffx.13949.1.S1_s_at | NR1H3 | 1.93±0.34 |
| CfaAffx.6867.1.S1_at | PRKAR2B | -1.92±0.95 |
| Cfa.13227.1.A1_at | SLC27A2 | -3.56±2.52 |
| CfaAffx.23380.1.S1_s_at | TNFRSF1A | 2.53±0.89 |
| **Steroid metabolism** | | |
| Cfa.3891.1.S1_at | APOC3 | -2.05±1.95 |
| CfaAffx.7812.1.S1_s_at | CUBN | -2.07±1.29 |
| CfaAffx.11088.1.S1_at | CYB5R2 | 2.27±0.6 |
| Cfa.204.1.S1_s_at | G6PC | -2.86±1.15 |
| Cfa.18474.1.S1_at | HINT2 | -2.05±0.38 |
| Cfa.10248.1.S1_at | HSD11B1 | -3.29±6.73 |
| CfaAffx.6781.1.S1_s_at | HSD17B14 | -2±1.09 |
| CfaAffx.30577.1.S1_at | HSD17B2 | -2.15±0.75 |
| Cfa.11107.1.A1_at | IDI1 | -4.2±0.23 |
| Cfa.12533.1.A1_at | INSIG1 | -2.17±0.99 |
| Cfa.11943.1.A1_at | RDH16 | 5.52±2.66 |
| Cfa.149.1.S1_at | SLC10A2 | -5.96±2.74 |
| Cfa.13227.1.A1_at | SLC27A2 | -3.56±2.52 |
| Cfa.70.1.A1_s_at | SULT1A1 | -2.33±0.41 |
| CfaAffx.5225.1.S1_s_at | SULT1B1 | -6.53±1.94 |
| Cfa.3680.1.S1_s_at | TIMP1 | 12.05±2.55 |
| Cfa.4426.1.S1_at | YWHAH | 2.24±0.53 |
| **Protein metabolism** | | |
| CfaAffx.2333.1.S1_at | A4GALT | 2.39±0.81 |
| CfaAffx.15587.1.S1_at | ACY1 | -2.64±1.03 |
| CfaAffx.13582.1.S1_at | ADAMTS1 | 4.39±1.54 |
| CfaAffx.20626.1.S1_at | APOA5 | 2.07±0.31 |
| CfaAffx.21464.1.S1_at | APOBEC1 | 2.15±0.83 |
| Cfa.3891.1.S1_at | APOC3 | -2.05±1.95 |
| Cfa.19985.1.S1_s_at | ASNS | 2.27±0.48 |
| Cfa.9097.1.A1_at | ATF3 | 4.69±2.55 |
| Cfa.12514.1.A1_at | BAG3 | 2.13±0.54 |
| Cfa.18376.1.S1_at | BIRC3 | 3.06±1.24 |
| CfaAffx.14883.1.S1_at | BTG2 | 2.31±0.65 |
| Cfa.16857.1.S1_at | C1QB | 1.95±0.69 |
| Cfa.10921.1.S1_at | C1QC | 2.77±0.96 |
| Cfa.10821.1.A1_s_at | C1RA | 7.33±1.77 |
| Cfa.12240.1.A1_at | C1S | 6.12±2.16 |
| Cfa.15481.1.A1_at | C3 | 6.12±2.01 |
| CfaAffx.8124.1.S1_s_at | C8G | -1.92±0.81 |
| Cfa.21507.1.S1_at | CAD | 2.54±0.14 |
| Cfa.1333.3.S1_a_at | CD46 | 5.34±0.98 |
| Cfa.5944.1.A1_at | CD74 | 5.56±0.97 |
| Cfa.6458.1.A1_s_at | CFB | 5.03±2.08 |
| CfaAffx.17824.1.S1_s_at | CFI | 2.15±0.61 |
| CfaAffx.18523.1.S1_s_at | CHEK2 | -3.84±3.53 |
| CfaAffx.30912.1.S1_at | CHST4 | 3.43±1.94 |
| Cfa.17292.1.S1_at | CLK1 | -1.9±0.33 |
| Cfa.1254.1.S1_s_at | CLU | 9.45±1.5 |
| CfaAffx.1014.1.S1_s_at | CNDP1 | -2.02±0.92 |
| CfaAffx.31268.1.S1_s_at | CTH | -2.28±2.11 |
| Cfa.21450.1.S1_s_at | CTSB | 3.75±2.03 |
| CfaAffx.7812.1.S1_s_at | CUBN | -2.07±1.29 |
| CfaAffx.15385.1.S1_s_at | CYLD | 2.01±0.41 |
| Cfa.16320.1.A1_s_at | DNAJC3 | 2.03±0.72 |
| Cfa.15083.1.S1_at | EFEMP1 | -3.08±1.34 |
| Cfa.3524.1.S1_s_at | EGF | -3.06±0.59 |
| CfaAffx.28520.1.S1_at | EGFLAM | -2.76±2.81 |
| Cfa.11431.1.A1_at | EIF4E2 | -1.87±0.23 |
| Cfa.11962.1.A1_at | EIF4EBP1 | 2.51±0.63 |
| CfaAffx.23648.1.S1_s_at | ETF1 | 2±0.59 |
| Cfa.7793.1.A1_at | FBXO31 | -2.23±1.36 |
| Cfa.12375.1.A1_at | FKBP5 | 9.47±3.8 |
| Cfa.1886.1.S1_at | FNIP2 | -1.9±0.97 |
| Cfa.204.1.S1_s_at | G6PC | -2.86±1.15 |
| CfaAffx.8335.1.S1_at | GALNT11 | -2.62±0.57 |
| Cfa.8296.1.A1_at | GLYCTK | -1.88±0.69 |
| CfaAffx.15349.1.S1_at | HERC6 | 2.06±0.24 |
| Cfa.126.1.S1_s_at | HIF1A | 5.21±2.61 |
| CfaAffx.27629.1.S1_s_at | HSP90AA1 | 2.09±0.25 |
| CfaAffx.30936.1.S1_s_at | HSPA5 | 2.68±0.72 |
| Cfa.7238.1.A1_at | IGFBP2 | 4.23±1.67 |
| CfaAffx.4021.1.S1_s_at | KIT | -1.95±0.55 |
| Cfa.15669.1.A1_s_at | KLHL36 | -1.98±0.42 |
| Cfa.12242.1.A1_at | KLK10 | -2.35±1.17 |
| CfaAffx.12001.1.S1_s_at | MAN2A1 | 2.46±1.01 |
| Cfa.13894.1.S1_s_at | MME | -3.22±0.74 |
| Cfa.5875.3.A1_x_at | MRPL22 | 2.06±0.72 |
| Cfa.20295.1.S1_at | MVP | 2.65±0.41 |
| Cfa.3786.1.S1_s_at | MYC | 3.68±1.89 |
| Cfa.8884.1.A1_s_at | NDNF | -2.46±1.05 |
| Cfa.10314.1.A1_s_at | NEK6 | 3.28±1.15 |
| Cfa.2700.1.A1_at | PCSK6 | -2.62±1.04 |
| Cfa.2593.1.S1_at | PLAUR | 1.95±0.6 |
| CfaAffx.11365.1.S1_at | PLK2 | -2.17±1.34 |
| Cfa.15679.1.A1_at | PM20D1 | -1.89±0.74 |
| CfaAffx.19910.1.S1_s_at | PPEF1 | -3.44±2.46 |
| CfaAffx.6867.1.S1_at | PRKAR2B | -1.92±0.95 |
| Cfa.12527.1.A1_at | PRKCSH | 3.52±1.12 |
| CfaAffx.31132.1.S1_at | PSMB10 | 3.21±0.47 |
| Cfa.12298.1.A1_a_at | PSMB8 | 2.05±0.11 |
| CfaAffx.19455.1.S1_s_at | PTPN14 | 1.95±0.44 |
| CfaAffx.13149.1.S1_s_at | PTPRJ | 2.14±0.29 |
| CfaAffx.10213.1.S1_at | QPCT | 2.03±0.13 |
| CfaAffx.5734.1.S1_at | RHBDL2 | 1.9±0.33 |
| CfaAffx.23004.1.S1_s_at | RPL22L1 | 3.27±1.6 |
| CfaAffx.20491.1.S1_at | SCRN3 | -2.11±0.53 |
| CfaAffx.19191.1.S1_at | SEC24D | 2.61±1.06 |
| CfaAffx.12561.1.S1_s_at | SERPING1 | 2.03±0.59 |
| CfaAffx.25142.1.S1_s_at | SLC34A1 | -2.29±2.07 |
| Cfa.16398.1.S1_at | SULF2 | 1.87±0.56 |
| Cfa.18951.1.S1_at | TFRC | 2.86±0.57 |
| CfaAffx.4346.1.S1_s_at | TINAG | -1.87±0.18 |
| Cfa.18701.1.S1_s_at | TMEM27 | -2.25±2.45 |
| CfaAffx.12600.1.S1_s_at | TRAM1 | 1.92±0.65 |
| Cfa.14859.1.A1_at | TUBB2B | 2.06±0.36 |
| Cfa.3348.1.A1_at | UBA7 | 2.07±0.63 |
| CfaAffx.5594.1.S1_at | UBE2J1 | 2.29±0.64 |
| CfaAffx.20837.1.S1_at | UCK2 | 2.05±0.41 |
| Cfa.111.1.A1_s_at | VWF | 1.91±0.31 |
| Cfa.13418.1.A1_at | WEE2 | -2.17±0.51 |
| CfaAffx.15227.1.S1_s_at | WFDC2 | 10.27±2.12 |
| Cfa.13201.1.S1_at | WNK4 | -1.92±0.64 |
| CfaAffx.20922.1.S1_at | ZBTB16 | 3.87±2.04 |
| **Transmembrane transport** | | |
| CfaAffx.3656.1.S1_s_at | ABCB4 | 3.08±1.61 |
| CfaAffx.17336.1.S1_s_at | ACACB | -3.82±1.85 |
| CfaAffx.19151.1.S1_s_at | ADCY4 | 3.5±1.35 |
| Cfa.19044.1.S1_at | ANO6 | 2.18±0.91 |
| Cfa.12876.1.S1_at | AQP1 | -2.26±1.42 |
| CfaAffx.8288.1.S1_s_at | AQP11 | -2.57±2.07 |
| Cfa.13671.1.A1_at | AQP6 | -2.09±1.27 |
| Cfa.3753.1.S1_at | CNGA1 | -2.39±0.65 |
| Cfa.1286.1.A1_at | CPT1A | 2.22±0.9 |
| Cfa.11362.1.A1_at | FOLR2 | 1.89±0.63 |
| Cfa.204.1.S1_s_at | G6PC | -2.86±1.15 |
| Cfa.11206.1.A1_at | GABRB3 | -1.96±0.52 |
| CfaAffx.27629.1.S1_s_at | HSP90AA1 | 2.09±0.25 |
| Cfa.3977.1.S1_at | LASP1 | 2.08±0.71 |
| Cfa.3786.1.S1_s_at | MYC | 3.68±1.89 |
| Cfa.2147.1.A1_at | NIPAL1 | -2.64±0.72 |
| CfaAffx.6867.1.S1_at | PRKAR2B | -1.92±0.95 |
| Cfa.35.1.A1_s_at | RHBG | -6.87±3.48 |
| Cfa.13599.1.A1_at | RHCE | 4.71±2.53 |
| Cfa.12674.1.A1_s_at | RYR1 | -2.35±0.23 |
| Cfa.11215.1.A1_at | SFXN1 | -2.35±0.95 |
| CfaAffx.1885.1.S1_s_at | SLC12A2 | 5.09±1.05 |
| Cfa.16325.1.S1_at | SLC12A4 | 1.94±0.19 |
| CfaAffx.28579.1.S1_at | SLC13A2 | -1.92±0.08 |
| Cfa.9574.1.S1_at | SLC15A2 | -2.46±1.19 |
| Cfa.14399.2.S1_s_at | SLC16A4 | -2.21±1.08 |
| CfaAffx.16647.1.S1_s_at | SLC17A3 | -2.17±1.02 |
| Cfa.1270.1.S1_s_at | SLC1A3 | -2.54±1.05 |
| Cfa.19866.1.S1_at | SLC1A4 | 1.94±0.66 |
| Cfa.14404.1.A1_s_at | SLC22A18 | -2.07±0.4 |
| CfaAffx.23727.1.S1_at | SLC22A8 | -2.13±0.47 |
| CfaAffx.14394.1.S1_at | SLC25A37 | 1.97±0.68 |
| CfaAffx.27927.1.S1_at | SLC26A2 | -6.81±3.54 |
| CfaAffx.6789.1.S1_at | SLC26A4 | 3.07±1.07 |
| CfaAffx.23994.1.S1_s_at | SLC2A9 | -1.89±0.58 |
| Cfa.5561.1.A1_at | SLC30A2 | -2.06±1.13 |
| CfaAffx.25142.1.S1_s_at | SLC34A1 | -2.29±2.07 |
| Cfa.13388.2.S1_at | SLC44A1 | -2.25±1.15 |
| CfaAffx.10961.1.S1_at | SLC46A3 | -2.6±0.52 |
| CfaAffx.27791.1.S1_s_at | SLC47A2 | -2.05±0.43 |
| CfaAffx.15913.1.S1_at | SLC5A12 | -2.69±3.29 |
| CfaAffx.11304.1.S1_at | SLC5A8 | -2.5±0.99 |
| Cfa.15653.1.A1_at | SLC6A19 | -4.36±2.62 |
| CfaAffx.21538.1.S1_at | SLC6A20 | -2.34±0.83 |
| CfaAffx.27366.1.S1_at | STRA6 | -2.3±0.91 |
| Cfa.3155.1.S1_at | TAP1 | 1.91±0.07 |
| Cfa.18951.1.S1_at | TFRC | 2.86±0.57 |

The functional properties of DEGs were analyzed using the GeneXplain platform and the p-value threshold was set as <0.05.
