# Supplementary material for: The pathogenesis of diclofenac induced immunoallergic hepatitis in a canine model of liver injury
Source: Oncotarget. 2017 Sep 23;8(64):107763–824. doi: 10.18632/oncotarget.21201 (PMC5746105; doi:10.18632/oncotarget.21201)
Supplement: Supplementary file 3 [file oncotarget-08-107763-s003.doc]

**Supplementary Table 4: Enhanced signaling pathways in liver and kidney after high dose diclofenac treatment**

| **Enriched pathways in liver** | | | |
| --- | --- | --- | --- |
| **Probeset ID** | **Gene symbol** | **Gene description** | **Fold change (average)±SD** |
| **Fc receptor signaling** | | | |
| Cfa.6259.1.A1_at | ARPC1A | Actin Related Protein 2/3 Complex Subunit 1A | 2.1±0.29 |
| Cfa.15172.1.A1_at | DOCK1 | Dedicator Of Cytokinesis 1 | 2.1±0.88 |
| Cfa.173.1.A1_s_at | FCGR1A (IgG) | Fc Fragment Of IgG Receptor Ia | 3.54±2.04 |
| Cfa.12195.14.S1_s_at | IGKC | Immunoglobulin Kappa Constant | 2.34±0.74 |
| Cfa.16944.1.S1_at | ITPR3 | Inositol 1,4,5-Trisphosphate Receptor Type 3 | 2.17±0.67 |
| CfaAffx.30317.1.S1_at | VAV2 | Vav Guanine Nucleotide Exchange Factor 2 | -2.01±0.6 |
| **PPAR signaling pathway** | | | |
| CfaAffx.21401.1.S1_at | ACADL | Acyl-CoA dehydrogenase, long chain | -2.33±1.37 |
| CfaAffx.31225.1.S1_at | ACADM | Acyl-CoA Dehydrogenase, C-4 To C-12 Straight Chain | -2.03±0.76 |
| Cfa.14057.1.A1_at | ACSL3 | Acyl-CoA synthetase long-chain family member 3 | 2.06±0.06 |
| Cfa.3891.1.S1_x_at | APOC3 | Apolipoprotein C3 | -3.55±1.03 |
| CfaAffx.22992.1.S1_at | CYP27A1 | Cytochrome P450 family 27 subfamily A member 1 | -2.51±3.82 |
| Cfa.4341.1.S1_at | DBI | Diazepam binding inhibitor, acyl-CoA binding protein | -2.15±0.81 |
| CfaAffx.20460.1.S1_s_at | EHHADH | Enoyl-CoA hydratase and 3-hydroxyacyl CoA dehydrogenase | -2.43±1.34 |
| CfaAffx.2395.1.S1_s_at | FABP7 | Fatty acid binding protein 7 | 2.34±0.98 |
| CfaAffx.21204.1.S1_s_at | GK | Glycerol kinase | 3.23±1.08 |
| Cfa.14368.1.A1_at | HMGCS2 | 3-hydroxy-3-methylglutaryl-CoA synthase 2 | -2.68±6.31 |
| CfaAffx.29303.1.S1_s_at | SCP2 | Sterol carrier protein 2 | -3.05±3.62 |
| Cfa.13227.1.A1_at | SLC27A2 | Solute carrier family 27 member 2 | -2.68±2.07 |
| CfaAffx.2004.1.S1_s_at | SLC27A6 | Solute carrier family 27 member 6 | -2.31±1.25 |
| **PI3K-Akt signaling pathway** | | | |
| Cfa.13686.1.A1_s_at | BCL2L11 | BCL2 like 11 | 2.33±0.84 |
| Cfa.11563.1.A1_at | CDKN1A | Cyclin dependent kinase inhibitor 1A | 5.1±1.17 |
| Cfa.14626.1.S1_at | COL3A1 | Collagen type III alpha 1 chain | -4.29±1.61 |
| CfaAffx.6792.1.S1_at | FGF21 | Fibroblast growth factor 21 | 3.87±1.56 |
| Cfa.204.1.S1_s_at | G6PC | Glucose-6-phosphatase catalytic subunit | -2.2±1.08 |
| CfaAffx.28402.1.S1_s_at | GHR | Growth hormone receptor | -2.06±0.97 |
| CfaAffx.10518.1.S1_s_at | HGF | Hepatocyte growth factor | 2.63±1.31 |
| Cfa.3888.1.S1_at | IGF1 | Insulin like growth factor 1 | -4.25±2.08 |
| CfaAffx.17252.1.S1_at | IL3RA | Interleukin 3 receptor subunit alpha | 2.06±0.78 |
| Cfa.34.1.S1_s_at | MCL1 | BCL2 family apoptosis regulator | 2.75±1.24 |
| CfaAffx.20679.1.S1_s_at | PPP2R3C | Protein phosphatase 2 regulatory subunit B''gamma | 2.09±0.85 |
| Cfa.9738.1.S1_s_at | PRKAA1 | Protein kinase AMP-activated catalytic subunit alpha 1 | 3.39±1.35 |
| CfaAffx.13822.1.S1_s_at | THBS1 | Thrombospondin 1 | 2.14±0.55 |
| Cfa.118.1.S1_at | TLR4 | Toll like receptor 4 | 2.11±0.74 |
| Cfa.111.1.A1_s_at | VWF | Von Willebrand factor | 4.22±1.71 |
| **MAPK cascade** | | | |
| CfaAffx.572.1.S1_at | DDIT3 | DNA damage inducible transcript 3 | 3.04±1.21 |
| CfaAffx.21064.1.S1_at | DUSP10 | dual specificity phosphatase 10 | -2.58±1.28 |
| Cfa.10153.1.S1_at | HSPA8 | heat shock protein family A | -8.24±1.57 |
| Cfa.3511.1.S1_at | IL1B | interleukin 1 beta | 2.02±0.16 |
| CfaAffx.4117.1.S1_at | IL1R1 | interleukin 1 receptor type 1 | 8.23±1.92 |
| Cfa.5221.1.A1_s_at | IL1R2 | interleukin 1 receptor type 2 | 4.96±2.1 |
| CfaAffx.20252.1.S1_s_at | PAK2 | p21 (RAC1) activated kinase 2 | 2.01±0.69 |
|  |  |  |  |
| **Enriched pathways in kidney** | | | |
| **Probeset ID** | **Gene symbol** | **Gene description** | **Fold change (average)±SD** |
| **PPAR signaling pathway** | | | |
| CfaAffx.12394.1.S1_s_at | ACSL1 | Acyl-CoA synthetase long-chain family member 1 | -4.23±2.01 |
| CfaAffx.27683.1.S1_s_at | ACSL4 | Acyl-CoA synthetase long-chain family member 4 | 6.25±3.38 |
| CfaAffx.17119.1.S1_s_at | ACSL5 | Acyl-CoA synthetase long-chain family member 5 | -2.6±1 |
| CfaAffx.2141.1.S1_s_at | ACSL6 | Acyl-CoA synthetase long-chain family member 6 | -1.95±0.44 |
| CfaAffx.20626.1.S1_at | APOA5 | Apolipoprotein A5 | 2.07±0.31 |
| Cfa.3891.1.S1_at | APOC3 | ApolipoproteinC3 | -2.05±1.95 |
| Cfa.1286.1.A1_at | CPT1A | Carnitine palmitoyltransferase 1A | 2.22±0.9 |
| CfaAffx.11994.1.S1_at | FABP1 | Fatty acid binding protein 1 | -2.61±1.29 |
| Cfa.299.1.A1_s_at | FABP3 | Fatty acid binding protein 3 | -3.71±0.89 |
| CfaAffx.109.1.S1_s_at | FABP5 | Fatty acid binding protein 5 | 2.66±0.44 |
| CfaAffx.13949.1.S1_s_at | NR1H3 | Nuclear receptor subfamily 1 group H member 3 | 1.93±0.34 |
| Cfa.17166.1.S1_at | PCK2 | Phosphoenolpyruvate carboxykinase 2, mitochondrial | -2.06±0.62 |
| Cfa.13227.1.A1_at | SLC27A2 | Solute carrier family 27 member 2 | -3.56±2.52 |
| **HIF-1 signaling** | | | |
| Cfa.11563.1.A1_at | CDKN1A | Cyclin dependent kinase inhibitor 1A | 2.64±0.36 |
| Cfa.125.1.S1_s_at | EDN1 | Endothelin 1 | 2.2±0.55 |
| Cfa.3524.1.S1_s_at | EGF | Epidermal growth factor | -3.06±0.59 |
| Cfa.11431.1.A1_at | EIF4E2 | Eukaryotic translation initiation factor 4E family member 2 | -1.87±0.23 |
| Cfa.11962.1.A1_at | EIF4EBP1 | Eukaryotic translation initiation factor 4E binding protein 1 | 2.51±0.63 |
| Cfa.126.1.S1_s_at | HIF1A | Hypoxia inducible factor 1 alpha subunit | 5.21±2.61 |
| Cfa.20307.1.S1_at | STAT3 | Signal transducer and activator of transcription 3 | 2.25±0.99 |
| Cfa.18951.1.S1_at | TFRC | Transferrin receptor | 2.86±0.57 |
| Cfa.3680.1.S1_s_at | TIMP1 | TIMP metallopeptidase inhibitor 1 | 12.05±2.55 |
| **PI3K-Akt signaling pathway** | | | |
| Cfa.11563.1.A1_at | CDKN1A | Cyclin dependent kinase inhibitor 1A | 2.64±0.36 |
| Cfa.8215.1.A1_at | DDIT4 | DNA damage inducible transcript 4 | 2.1±0.73 |
| Cfa.3524.1.S1_s_at | EGF | Epidermal growth factor | -3.06±0.59 |
| Cfa.11431.1.A1_at | EIF4E2 | Eukaryotic translation initiation factor 4E family member 2 | -1.87±0.23 |
| Cfa.11962.1.A1_at | EIF4EBP1 | Eukaryotic translation initiation factor 4E binding protein 1 | 2.51±0.63 |
| Cfa.14872.1.A1_at | FGF9 | Fibroblast growth factor 9 | -2.15±0.83 |
| Cfa.204.1.S1_s_at | G6PC | Glucose-6-phosphatase catalytic subunit | -2.86±1.15 |
| CfaAffx.27629.1.S1_s_at | HSP90AA1 | Heat shock protein 90 alpha family class A member 1 | 2.09±0.25 |
| Cfa.10009.1.A1_at | IFNAR2 | Interferon alpha and beta receptor subunit 2 | 2.37±0.48 |
| CfaAffx.4021.1.S1_s_at | KIT | KIT proto-oncogene receptor tyrosine kinase | -1.95±0.55 |
| Cfa.14195.1.A1_s_at | LAMA1 | Laminin subunit alpha 1 | -3.68±2.24 |
| Cfa.34.1.S1_s_at | MCL1 | BCL2 family apoptosis regulator | 2.03±0.53 |
| Cfa.3786.1.S1_s_at | MYC | v-myc avian myelocytomatosis viral oncogene homolog | 3.68±1.89 |
| Cfa.2080.1.S1_at | OSMR | Oncostatin M receptor | 13.47±3.46 |
| Cfa.17166.1.S1_at | PCK2 | Phosphoenolpyruvate carboxykinase 2, mitochondrial | -2.06±0.62 |
| Cfa.4923.1.A1_s_at | RAC1 | Ribonuclease A family member 1, pancreatic | 2.27±0.71 |
| CfaAffx.15042.1.S1_s_at | SPP1 | Secreted phosphoprotein 1 | 5.28±0.93 |
| Cfa.111.1.A1_s_at | VWF | Von Willebrand factor | 1.91±0.31 |
| Cfa.4426.1.S1_at | YWHAH | Tyrosine 3-monooxygenase/tryptophan 5-monooxygenase activation protein eta | 2.24±0.53 |
| **Insulin signaling pathway** | | | |
| CfaAffx.17336.1.S1_s_at | ACACB | Acetyl-CoA carboxylase beta | -3.82±1.85 |
| Cfa.11431.1.A1_at | EIF4E2 | Eukaryotic translation initiation factor 4E family member 2 | -1.87±0.23 |
| Cfa.11962.1.A1_at | EIF4EBP1 | Eukaryotic translation initiation factor 4E binding protein 1 | 2.51±0.63 |
| Cfa.17136.1.S1_at | FASN | Fatty acid synthase | -4.87±2.22 |
| Cfa.17541.1.S1_s_at | FBP1 | Fructose-bisphosphatase 1 | -1.9±1.08 |
| Cfa.204.1.S1_s_at | G6PC | Glucose-6-phosphatase catalytic subunit | -2.86±1.15 |
| CfaAffx.29270.1.S1_at | INPP5K | Inositol polyphosphate-5-phosphatase K | 2.14±0.7 |
| Cfa.17166.1.S1_at | PCK2 | Phosphoenolpyruvate carboxykinase 2, mitochondrial | -2.06±0.62 |
| CfaAffx.6867.1.S1_at | PRKAR2B | Protein kinase cAMP-dependent type II regulatory subunit beta | -1.92±0.95 |
| **Adipocytokine signaling** | | | |
| CfaAffx.17336.1.S1_s_at | ACACB | Acetyl-CoA carboxylase beta | -3.82±1.85 |
| CfaAffx.12394.1.S1_s_at | ACSL1 | Acyl-CoA synthetase long-chain family member 1 | -4.23±2.01 |
| CfaAffx.27683.1.S1_s_at | ACSL4 | Acyl-CoA synthetase long-chain family member 4 | 6.25±3.38 |
| CfaAffx.17119.1.S1_s_at | ACSL5 | Acyl-CoA synthetase long-chain family member 5 | -2.6±1 |
| CfaAffx.2141.1.S1_s_at | ACSL6 | Acyl-CoA synthetase long-chain family member 6 | -1.95±0.44 |
| Cfa.1286.1.A1_at | CPT1A | Carnitine palmitoyltransferase 1A | 2.22±0.9 |
| Cfa.204.1.S1_s_at | G6PC | Glucose-6-phosphatase catalytic subunit | -2.86±1.15 |
| Cfa.12441.1.S1_s_at | NFKBIA | NFKB inhibitor alpha | 2.1±0.81 |
| Cfa.17166.1.S1_at | PCK2 | Phosphoenolpyruvate carboxykinase 2, mitochondrial | -2.06±0.62 |
| Cfa.20307.1.S1_at | STAT3 | Signal transducer and activator of transcription 3 | 2.25±0.99 |
| CfaAffx.23380.1.S1_s_at | TNFRSF1A | TNF receptor superfamily member 1A | 2.53±0.89 |
